# Development of a live attenuated trivalent porcine rotavirus A vaccine against disease caused by recent strains most prevalent in South Korea

**DOI:** 10.1186/s13567-018-0619-6

**Published:** 2019-01-07

**Authors:** Jun-Gyu Park, Mia Madel Alfajaro, Eun-Hyo Cho, Ji-Yun Kim, Mahmoud Soliman, Yeong-Bin Baek, Chul-Ho Park, Ju-Hwan Lee, Kyu-Yeol Son, Kyoung-Oh Cho, Mun-Il Kang

**Affiliations:** 1 Laboratory of Veterinary Pathology, College of Veterinary Medicine, Chonnam National University, Gwangju, Republic of Korea; 2 Chonnam National University Veterinary Teaching Hospital, Gwangju, 500-757 Republic of Korea; 3 Choong Ang Vaccine Laboratory, Daejeon, Republic of Korea

## Abstract

Porcine rotaviruses cause severe economic losses in the Korean swine industry due to G- and P-genotype mismatches between the predominant field and vaccine strains. Here, we developed a live attenuated trivalent porcine group A rotavirus vaccine using 80 cell culture passages of the representative Korean predominant strains G8P[7] 174-1, G9P[23] PRG942, and G5P[7] K71. Vaccination with the trivalent vaccine or its individual components induced no diarrhea during the first 2 weeks post-vaccination, i.e., the vaccines were attenuated. Challenge of trivalent-vaccinated or component-vaccinated piglets with homologous virulent strain(s) did not induce diarrhea for 2 weeks post-challenge. Immunization with the trivalent vaccine or its individual components also alleviated the histopathological lesions in the small intestines caused by challenge with the corresponding original virulent strain(s). Fecal secretory IgAs specific for each of vaccine strains were detected starting at 14 days post-vaccination (dpv), and IgA levels gradually increased up to 28 dpv. Oral immunization with the trivalent vaccine or its individual components induced high levels of serum virus-neutralizing antibody by 7 dpv. No diarrhea was observed in any experimental piglets during five consecutive passages of each vaccine strain. Our data indicated that the live attenuated trivalent vaccine was safe and effective at protecting piglets from diarrhea induced by challenge exposure of homologous virulent strains. This trivalent vaccine will potentially contribute toward controlling porcine rotavirus disease in South Korea and other countries where rotavirus infections with similar G and P genotypes are problematic.

**Electronic supplementary material:**

The online version of this article (10.1186/s13567-018-0619-6) contains supplementary material, which is available to authorized users.

## Introduction

Group A rotaviruses (RVAs), members of the *Reoviridae* family, are among the most important enteric pathogens causing severe dehydrating diarrhea in young children and a wide variety of young animals [1–3]. RVA is a non-enveloped, triple-layered capsid virus possessing eleven double-stranded (ds) RNA segments that encode six structural proteins (VP1–V4, VP6, and VP7) and six nonstructural proteins (NSP1–NSP6) [1, 4, 5]. Due to the nature of a segmented genome, RVAs reassert in cells infected with different RVAs, potentially leading to the emergence of novel progeny viruses [1].

Each genomic segment of RVA is classified into independent genotypes based on nucleotide percentage identity cut-off values [6, 7]. The combination of VP7-G genotypes (G stands for glycoprotein) and VP4-P genotypes (P stands for protease-sensitive) is frequently used for RVA classifications, as the encoded proteins are highly involved in immune protection and commonly used in vaccine development [1, 8]. Currently, 35 G and 50 P genotypes have been identified from various countries and animal species and resulting in various combinations of G and P genotypes [9].

Porcine RVAs are identified as a common pathogen causing gastroenteritis in neonatal piglets worldwide [10]. In pigs, twelve G genotypes (G1 to G6, G8 to G12, and G26) in combination with sixteen P genotypes (P[1] to P[8], P[11], P[13], P[19], P[23], P[26], P[27], P[32], and P[34]) have been identified to date [10]. Among these, the most common genotype combinations detected in pigs are G5P[7], G4P[6], G4P[6], and G4P[7] [10–13]. Given this, pig RVA vaccines worldwide contain porcine RVA strains exhibiting these prevalent G and P genotypes, including the bivalent live ProSystem RCE vaccine manufactured by Intervet/Merck Animal Health, which contains strains G5P[7] OSU, G4P[6] Gottfried, and G7P[7] A2 [10, 14]. Depending on the geographical location and the particular time, however, prevalent G and P genotypes differ and change [10, 12]. For example, the G and P genotype combination of Canadian porcine RVAs changed from G5 to P[7] during 1982–1984 to G4, G5, G9, and G2 in combination with P[6], P[27], and P[13] during 2005–2006 [10].

In South Korea, many pig farms have used porcine RVA vaccines that are sold worldwide or domestically developed. Despite the use of these vaccines, porcine RVA infections and associated disease remain widespread and highly prevalent at 38.3% in South Korea [15]. Over time, molecular genotyping of Korean porcine RVA strains has revealed that G- and P-genotype combinations detected in Korean porcine RVA strains include G5P[7], G8P[7], G9P[7], G9P[23], and G8P[1] [15]. It must be noted that the domestic bivalent vaccine (containing G5P[7]-A1 and G9P[7]-10 strains) or imported vaccines such as the ProSystem RCE vaccine may not protect well against infections caused by G8P[7], G9P[23], and G8P[1] RVA strains. This may explain why porcine RVA infections are endemic on Korean pig farms, even though vaccines have been used. Therefore Korean swine farmers and veterinarians have strongly demanded that updated porcine RVA vaccines be produced, and this demand prompted us to develop a live attenuated trivalent porcine RVA vaccine using the prevalent South Korean strains. In this study, three representative strains, G8P[7] strain 174-1, G9P[23] strain PRG942, and G5P[7] strain K71, were chosen based on findings from a previous molecular epidemiological study [15]. The strains were passaged up to 80 times in MA104 cells and then evaluated for efficacy and safety.

## Materials and methods

### Viruses, cells, and serial passages

African green monkey kidney epithelial MA104 cells obtained from the American Type Culture Collection (ATCC, Manassas, VA, USA) were grown in alpha minimal essential medium supplemented with 10% fetal bovine serum (FBS), 100 U/mL penicillin, and 100 μg/mL streptomycin. Based on previously reported molecular epidemiological data [15], three porcine RVA strains, G8P[7] 174-1, G5P[7] K71, and G9P[23] PR942, which are representative of the most prevalent G and P genotype combination, were chosen from archived porcine RVA strains in Laboratory of Veterinary Pathology, College of Veterinary Medicine, Chonnam National University. These strains were isolated from fecal samples of diarrheic piglets in South Korea during 2006–2007 [15]. Genotype constellations of these strains are G8-P[7]-I5-R1-C1-M2-A1-N1-T1-E1-H1 for strain 174-1, G5-P[7]-I5-R1-C1-M1-A1-N1-T1-E1-H1 for strain K71, and G9-P[23]-I5-R1-C1-M1-A8-N1-T1-E1-H1 for strain PRG942 (Additional file 1) [16, 17]. The three strains were passaged eighty times in confluent MA104 cells including initial adaptation and triple plaque purification prior to the attenuation of each strain as previously described [15–18].

### Cell culture immunofluorescence (CCIF) assay

Virus titers were determined using CCIF assays as previously described [19, 20]. Briefly, serial dilutions of virus supernatants were incubated with 10 μg/mL of crystalized trypsin (Cat. No. 27250-018, Gibco, Fort Worth, Texas, USA) for 1 h and inoculated into confluent MA104 cells grown on 96-well plates. After incubation for 16 h, the cells were fixed with 100% cold acetone for 10 min and then washed twice with phosphate buffered saline (PBS, pH 7.2). The plates were incubated with a 1:100 dilution of a monoclonal antibody against the VP6 protein of porcine RVA strain OSU [19] for 1 h at room temperature. After washing three times with PBS, the plates were incubated with a 1:200 dilution of a fluorescein isothiocyanate (FITC)-conjugated goat anti-mouse IgG antibody in PBS (pH 7.8). Virus titers were calculated as fluorescence focus units per milliliter (FFU/mL).

### RNA extraction, reverse transcription-polymerase chain reaction (RT-PCR), and DNA sequencing

Total RNA from the lysates of RVA-infected MA104 cells or supernatant of tenfold PBS-diluted fecal samples were extracted using an Accuprep^®^ Viral RNA Extraction kit (Bioneer, Daejeon, South Korea) according to manufacturer instructions [16]. To detect the presence of viral RNA in the fecal samples from experimental animals, RT-PCR assays were performed using primer pairs specific for a partial region of the RVA VP6 gene (Additional file 2). The PCR products were electrophoretically separated and detected on 1.2% agarose gels stained with RedSafeTM (iNtRON Biotechnology, Gyeonggi-do, South Korea). To determine the full-genomic sequences of the 11 genomic segments, RT-PCR and 5′ and 3′ RACE PCR assays using primer pairs specific for each of the genomic segments (Additional file 2) were performed as described previously [16]. Purification of the PCR products, cloning, and DNA sequencing were all performed as previously described [16].

### Quantification of viral RNA by real-time RT-PCR using SYBR Green chemistry

A one step real-time RT-PCR assay using a primer pair specific to the VP6 gene of RVAs was performed to quantify the RNA in samples as described previously [21–23]. Briefly, total RNA was extracted from clarified supernatants of each passage of the porcine vaccine candidates using an Accuprep^®^ Viral RNA Extraction kit (Bioneer) as indicated in the manufacturer’s instructions. The extracted RNA was immediately processed or stored at −80 °C until use. Real-time RT-PCR was performed using a Corbett Research Rotor-Gene Real-Time Amplification system (Corbett Research, Mortlake, Australia) and SensiFast™ SYBR^®^ Lo-ROX One-Step Kit (Bioline, London, UK). A final volume of 20 µL containing 5 µL of RNA template, 10 µL SensiFast™ mix, 1 µL each of 0.5 M forward and reverse primers (final concentration of each primer: 20 nM), 0.25 µL Reverse transcriptase, 0.5 µL RNase inhibitor, and 2.25 µL RNase-free water was prepared for the real-time RT-PCR assay. Reverse transcription was carried out at 50 °C for 30 min, followed by the activation of the hot-start DNA polymerase at 95 °C for 15 min. Forty-three-step cycles were performed as follows: 95 °C for 15 s, 51 °C for 30 s, and 72 °C for 20 s. Quantification was carried out using a standard curve generated from serial tenfold dilutions of an in vitro transcribed complementary RNA (cRNA) amplified in separate PCR tubes. Rotor-Gene 6000^®^ software was used to calculate the amount of RVA RNA in the samples. The threshold was defined automatically in the start of the exponential phase, reflecting the highest amplification rate. The Rotor-Gene 6000^®^ software created a standard curve that allowed the determination of the amount of RVA RNA present in the samples by linear regression analysis.

### Polyacrylamide gel electrophoresis (PAGE)

To analyze genotypic patterns of the 11 genomic double-strand RNAs of the RVA strains, PAGE was performed for the original virulent porcine strains 174-1, PRG942, and K71 and for every 20^th^ passages of the strains as described previously [24, 25]. Briefly, total RNA was extracted from the lysate of MA104 cells infected with each virulent or attenuated RVA strain, and RNA was then analyzed using a 7.5% resolving and 5% stacking RNA-PAGE gel. After 20 h of electrophoresis, the gel was silver stained as previously described to visualize the bands [24].

### Molecular characterization of virulent and attenuated viruses

The entire nucleotide and deduced amino acid sequences of the full-length open-reading frames (ORFs) of each genomic segment (nucleotide sequence regions based on 174-1, PRG942, and K71 strains: VP7: 49 to 1029; VP4: 10 to 2295~2340; VP6: 24 to 1217; VP1: 19 to 3285; VP2: 16~17 to 2688~2689; VP3: 50~59 to 2557~2566; NSP1: 32 to 1492; NSP2: 47 to 1000; NSP3: 26 to 967~979; NSP4: 42 to 569; NSP5: 22 to 615) of the original virulent and every 20^th^ passage of the three porcine strains (porcine 174-1, PRG942, and K71) and those of the other known RVA strains were multi-aligned and trimmed using MEGA 6 software [26]. Pairwise distance between the study strains and the reference strains was calculated at the nucleotide level using the Proportional (*p*)-distance model. Phylogenetic trees were constructed using the Maximum Likelihood method based on the General Time Reversible (GTR) (VP1 to 4, VP6 to 7, NSP1 to 3 and NSP5) or the Neighbor-joining method based on the Kimura-2 model (NSP4) with gamma distributed substitution rates. The robustness of branching patterns was tested by 500 bootstrap replicates. Pairwise distance and phylogenetic trees were both constructed using Mega 6 software [26].

### GenBank accession numbers

The GenBank accession numbers of the RVA strains used in this study are listed in Additional file 3.

### Experimental animals

To evaluate the safety of the trivalent and its individual components, 72 7-week-old BALB/c mice (15~20 g), 36 7-week-old Dunkin-Hartley guinea pigs (300–350 g), and 18 4-week-old piglets (10 kg) were used. A total of 91 colostrum-deprived piglets aseptically obtained from sows by hysterectomy were maintained in gnotobiotic isolator units with automatically controlled temperatures. Piglets for each treatment group were raised in separated isolator units. All piglets were seronegative for RVA antibodies prior to exposure to RVA virulent or vaccine strains. These animals were fed autoclaved commercial piglet formula in liquid form four times a day. These piglets were used to evaluate the median diarrheic dose (DD_50_), virulence reversion, and the efficacy of each vaccine strain. The number of animals used was based on the animal welfare guidelines. All procedures were approved by the Institutional Animal Care and Use Committee of Chonnam National University (CNU IACUC-YB-R-2016-66) and the Choong Ang Vaccine Corporation (160129-04).

### Median diarrheic dose (DD_50_) of the virulent strains

At three days of age, 36 colostrum-deprived piglets were randomly divided into twelve groups. Each group of three piglets was orally inoculated with diluent (mock inoculated), 1 × 10^1^, 1 × 10^2^, or 1 × 10^3^ FFU/1 mL of each of the virulent strains (174-1, K71, or PRG942). Determination of diarrhea was evaluated at 3 days post-inoculation (dpi) based on fecal consistency scored using a 5-point rating system of 0 (normal), 1 (pasty), 2 (semi-mucoid), 3 (liquid), and 4 (profuse diarrhea) [16]. The DD_50_ was calculated as previously described and expressed as FFU/mL [27].

### Efficacy testing

A total of 25 three-day-old colostrum-deprived piglets obtained from sows by hysterectomy were used to evaluate the efficacy of the live attenuated trivalent vaccine and its individual components (strains 174-1V-80, K71V-80, and PRG942V-80). Each group of five piglets was orally immunized with 1 mL of each of three live monovalent vaccine strains 174-1V80 (5.6 × 10^3^ FFU/mL), PRG942V-80 (3.1 × 10^4^ FFU/mL), K71V-80 (3.1 × 10^3^ FFU/mL) or trivalent vaccine containing the three monovalent vaccine strains at their respective titers above (Table 1). The virus titer of each monovalent vaccine was equivalent to 1 × 10^2^ DD_50_ of its original virulent strain. As a negative control, five three-day-old colostrum-deprived piglets were inoculated with 1 mL supernatant from the mock-infected MA104 cell culture supernatant (Table 1).

**Table 1:** **Efficacy testing of porcine monovalent and trivalent vaccine candidates**

Vaccine strain/s	No. of animals	Route of administration	Amount given (mL)	Titer
174-1V-80 (G8P[7])	5	Oral	1	5.6 × 10^3^ (FFU^b^/mL)
K71V-80 (G9P[23])	5	Oral	1	3.1 × 10^3^ (FFU/mL)
PRG942V-80 (G5P[7])	5	Oral	1	3.1 × 10^4^ (FFU/mL)
Trivalent (mixture of 174-1V-80, K71V-80, PRG942V-80)	5	Oral	1	1 × 10^2^ DD_50_^c^
Mock-inoculated^a^	5	Oral	1	–

^a^Inoculated with serum-free α-MEM.

^b^FFU: Fluorescence focus unit.

^c^The virus titer of individual vaccine strain equivalent to diarrhea dose _50_ (DD_50_) of its virulent strain.

At 2 weeks post-vaccination, piglets were challenged with equivalent titers of their respective immunizing doses and observed for an additional 2 weeks (Table 1). Beginning prior to vaccination and continuing throughout the experiment, all the piglets were observed daily and evaluated for clinical signs such as diarrhea. All animals were necropsied at 28 days post-vaccination (dpv), and intestinal and extra-intestinal organs were collected and immediately fixed in 10% neutral formalin for histopathological examination (Additional file 4). Fecal samples were collected daily for the designated periods, and their consistency was evaluated as described above [17]. Fecal samples were diluted 1:10 in PBS, and the supernatants were collected following centrifugation at 12 000 × *g* for 10 min at 4 °C. Blood samples were collected at weekly intervals from 0 to 28 dpv. Serum was collected following centrifugation at 1000 × *g* for 10 min at 4 °C. The serum samples were heat inactivated at 56 °C for 30 min. The fecal supernatants and serum samples were analyzed by RT-PCR assays to assess viral genome copy numbers, by virus neutralization (VN) assays, and by enzyme-linked immunosorbent assays (ELISA) for determination of fecal and serum IgM, IgG, and IgA levels [16, 28].

### Virus neutralization (VN) assays

Serum samples obtained from the experimental piglets were used for determining VN titers against porcine RVA strains 174-1, K71, and PR942 as described previously [28]. Briefly, serially two-fold diluted sera were mixed with 500 FFU of each trypsin pre-activated RVA strain and incubated for 1 h at 37 °C. Each mixture was transferred into each well of 96-well plates confluent with MA104 cells and allowed to incubate for 16 h at 37 °C. The plates were then fixed with cold acetone, and their titers were measured using an indirect CCIF test. The VN titers were calculated as the reciprocal of the highest dilution that reduced more than 70% of RVA positive cells compared to that of rotavirus-infected controls.

### Enzyme-linked immunosorbent assays (ELISAs)

Sera and fecal samples obtained from the experimental animals were analyzed for RVA-specific antibodies by ELISAs as described previously [28]. Briefly, 96-well plates were coated with 1 × 10^6^ FFU of each porcine RVAs strain (174-1, K71, and PRG942) in PBS containing 5% bovine serum albumin (BSA) and then incubated at 4 °C overnight. Aliquots of two-fold serially-diluted serum or fecal supernatant in 5% BSA buffer were transferred into each corresponding 96-well plate and then incubated at 37 °C for 1 h. The plates were then incubated with biotin-conjugated goat anti-pig IgM, IgA, or IgG antibodies. Plates were further incubated with streptavidin and tetramethyl benzidine (KOMA Biotechnology, Seoul, South Korea), followed by the addition of a stop solution of 1 N HCl. Optical density (OD) was measured at 450 nm using an ELISA plate reader (Thermo Fisher scientific, MA, USA). The serum antibody titer was calculated as the reciprocal of the highest dilution where the mean OD was higher than the cutoff value (= mean OD of the negative control well + three standard deviations above).

### Histopathological examination

Formalin-fixed paraffin-embedded 3 μm sections of each small intestinal segment (duodenum, jejunum, and ileum) were stained with Meyer’s hematoxylin and eosin and microscopically examined. Histopathological changes in the small intestinal mucosa were scored using criteria including the average villi/crypt (V/C) ratio plus the grade of epithelial cell desquamation as described previously [29].

### Safety test

The pig derived RVAs could be naturally attenuated in heterologous experimental animals such as guinea pigs or BALB/c mice. If the vaccine is contaminated with other virulent pathogens, harmful chemicals, or toxins, it can cause disease in laboratory animals. Therefore, the Animal and Plant Quarantine Agency, Republic of Korea, requires that attenuated porcine RVA vaccines should be safe to ensure that they do not cause any clinical signs or pathology in BALB/c mice, guinea pigs, and piglets. In accordance with Agency guidelines, the safety of each live attenuated monovalent vaccine was analyzed using 7-week-old BALB/c mice, 7-week-old Dunkin-Hartley guinea pigs, and 4-week-old piglets (Additional file 5). Each vaccine strain was inoculated by intraperitoneal (IP) inoculation into 24 mice, by intramuscular (IM) inoculation into 6 piglets, and by either IM or IP inoculation into six guinea pigs (Additional file 5). All animals were evaluated daily for clinical signs and fecal consistency for 7 days post-inoculation and then euthanized and necropsied.

### Virulence reversion

Pairs of 3-day-old colostrum-deprived piglets were orally inoculated with 1 mL (1 × 10^6^ FFU/mL) of each 80^th^-passage of vaccine strains (174-1V-80, K71V-80, or PRG942V-80 strains). All piglets were monitored daily for clinical signs and fecal consistency and euthanized at 5 dpv. Duodenum specimens and their fecal content were aseptically collected from the piglets at necropsy, homogenized in PBS (10% w/v) containing 1% gentamicin, and then centrifuged at 1000 ×* g* at 4 °C for 10 min. The supernatants were filtered through 0.2 μm membranes, and the virus titers were measured by CCIF assays as described above. Filtered supernatants at a dose of 1 × 10^6^ FFU/mL were inoculated into pairs of 3-day-old colostrum-deprived piglets for each group. Serial passages were conducted a total of five times.

## Results

### Virus attenuation

**Figure 1:**
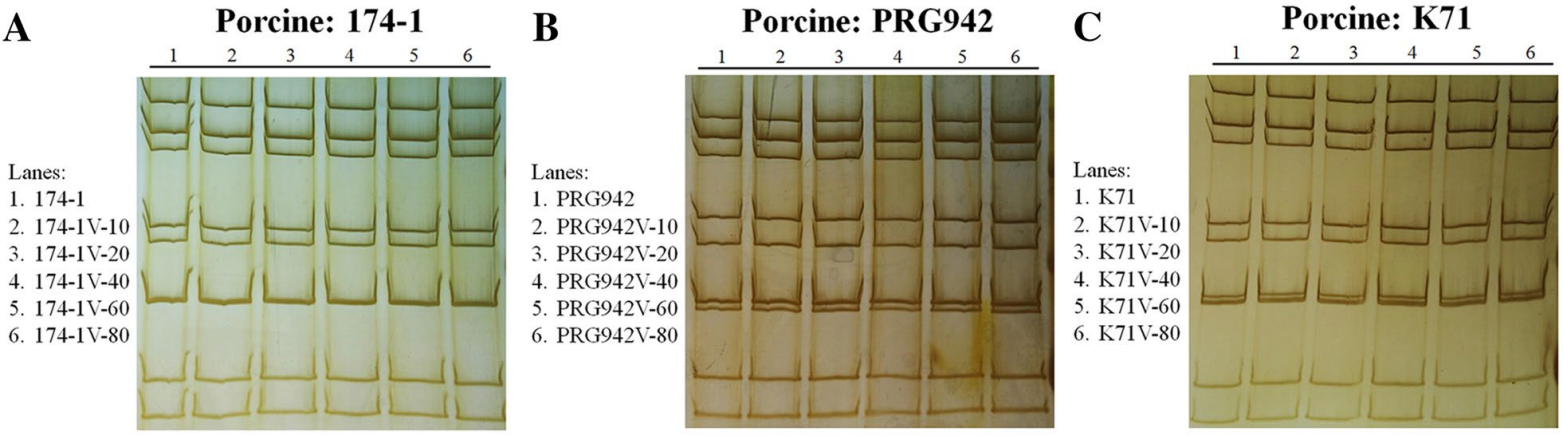
**Electropherogram (PAGE) of selected passages in MA104 cells of three porcine RVA strains.** The RNA genomic segments of three vaccine strains from different passage numbers were separated by PAGE and visualized using silver staining. The original virulent strains and each passage of 174-1 (**A**), K71 (**B**), and PRG942 (**C**) demonstrated typical RVA’s RNA segment patterns of 4-2-3-2 and maintained their own patterns throughout the serial passages. Lane 1, original virulent strain; lane 2, 10^th^ passage; lane 3, 20^th^ passage; lane 4, 40^th^ passage; lane 5, 60^th^ passage; lane 6, 80^th^ passage.

Serial passage of porcine RVA strains 174-1, PRG942, and K71 was performed in confluent MA104 cells through the 80^th^ passage. Virulent and attenuated porcine RVA vaccine strains were titered at different passage numbers by CCIF assay and real-time RT-PCR assay. The virus titer of each strain increased with passaging and the genome copy numbers for each passage were amplified to 10–100 times greater than virus titers (Additional file 6). Based on PAGE analysis, there were no distinctive changes in the PAGE patterns between the original virulent strains and their passaged strains that were evaluated at intervals of every 20^th^ passage (Figure 1). These data indicated that the three vaccine strains were well adapted to MA104 cells and that genome rearrangements caused by serial passage at high multiplicity of infection had not occurred during serial passage in the MA104 cells [30].

### Phylogenetic and amino acid mutation analyses

Phylogenetic and homology analyses were performed on the full-length ORF nucleotide sequences of the 11 genomic segments obtained from the original strains and the 20^th^, 40^th^, 60^th^, and 80^th^ passaged attenuated strains. Results showed that the 11 genomic segments of the original strain and of each 20-passage-interval strains clustered tightly and shared high nucleotide identity (Additional files 7, 8, 9, 10). These results revealed that the 80 serial passages of each of the original virulent strains did not influence any genotypic changes due to mutations of large regions.

To determine if amino acid mutation(s) within some of the genes were possibly involved in the loss of virulence, the full-length amino acid sequences of the 11 genomic segments of the 80^th^-passage attenuated vaccine strains were compared with those of the 20^th^-, 40^th^-, and 60^th^-passage attenuated strains and the original virulent strains (Additional files 11, 12, 13). Comparison of the 174-1V-80 vaccine strain with its original 174-1 strain and its less attenuated strains (174-1V-20, 174-1V-40, and 174-1V-60) revealed 26 amino acid substitutions in 10 of the genomic segments (Additional file 11). Among the substitutions at the 80^th^ passage, four out of twenty-six were reversions to the original amino acid sequences. Thus, 22 amino acid substitutions remained (Additional file 11). In strain PRG942V-80, 109 amino acid substitutions were found with 45 of these reverting to their original sequences at the 80^th^ passage (Additional file 12). In strain K71V-80, a total of 69 amino acid substitutions were observed with 19 amino acids reverting to original amino acid sequences at the 80^th^ passage (Additional file 13). These results suggested that some or all of these amino acid substitutions may have been involved in the loss of virulence. It is of interest that the number of amino acid changes acquired during the passaging of RVAs was highly variable among the different strains.

### Rotavirus vaccine prevents diarrhea caused by virulent strains

**Figure 2:**
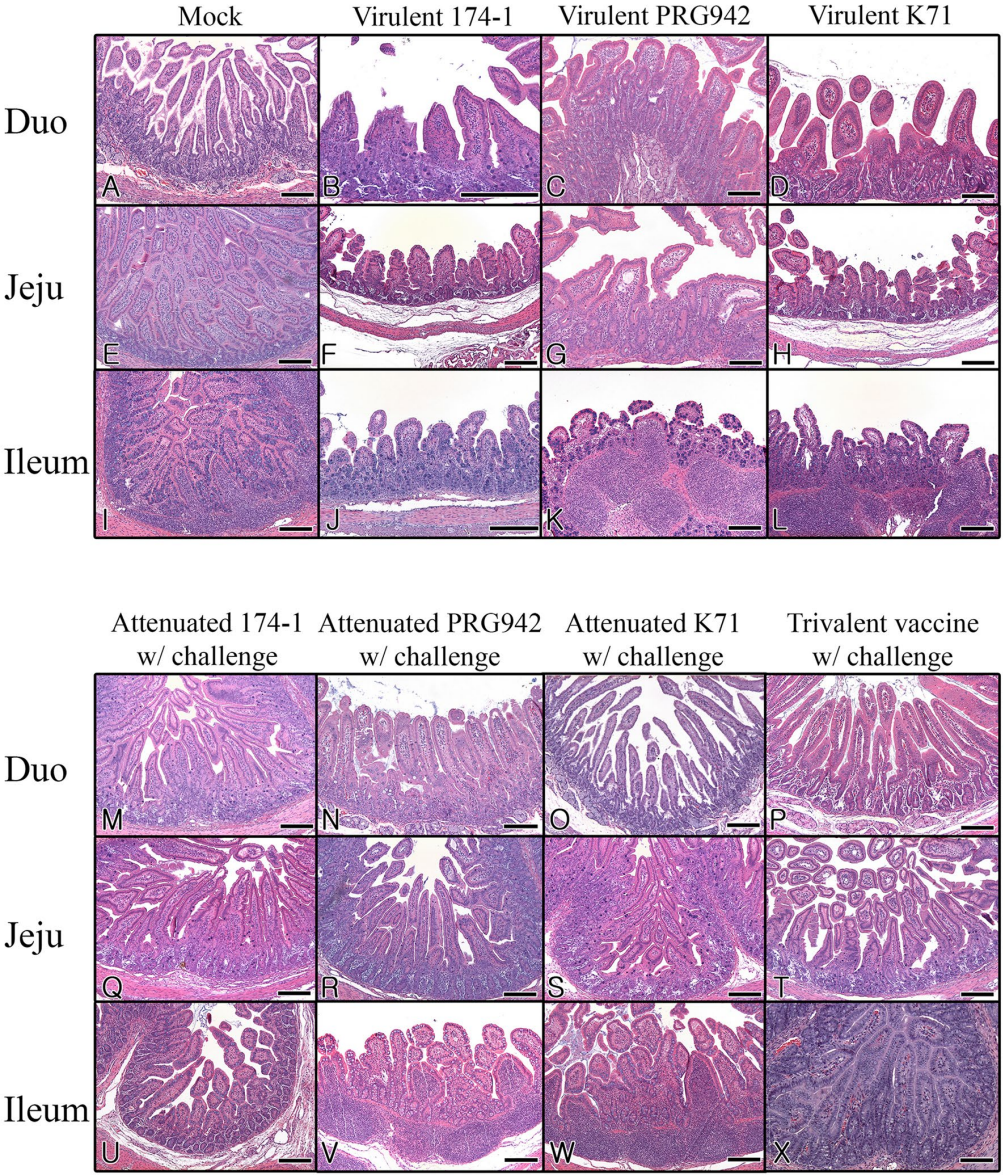
**Histopathological changes in piglets infected with each virulent RVA strain or after challenge following immunization.** (**A**, **E**, **I**) Duodenum, jejunum, and ileum of mock-inoculated piglets showed villi of normal length and no crypt hyperplasia. (**B**–**D**, **F**–**H**, and **J**–**L**) Duodenum (**B**–**D**), jejunum (**F**–**H**), and ileum (**J**–**L**) sampled at 7 dpi from piglets inoculated with virulent strains 174-1, PRG942, or K71 showed severe villous atrophy, moderate crypt hyperplasia, and severe lymphoid cell infiltrations in the lamina propria. (**M**–**X**) Duodenum (**M**–**P**), jejunum (**Q**–**T**), and ileum (**U**–**X**) sampled at 28 dpv from piglets first immunized with one of the live attenuated monovalent or trivalent vaccines and then challenged with the corresponding original virulent strain(s) 174-1, K71 or PRG942 showed villi of normal length, mild crypt hyperplasia, and mild infiltrations of lymphoid cells in the lamina propria in a manner similar to that of the mock-inoculated group. Hematoxylin and eosin stain. Bars = 200 μm.

Before determining the vaccine efficacy, DD_50_ values of the original virulent strains 174-1, PRG942, and K71 were measured using three-day-old colostrum-deprived piglets. Results showed that the DD_50_ was 5.6 × 10^1^ FFU for strain 174-1, 3.1 × 10^2^ FFU for strain PRG942, and 3.1 × 10^1^ FFU for strain K71, indicating that these strains were highly virulent in piglets. Histopathological observation showed that strains 174-1, PRG942, and K71 induced severe villous atrophy and fusions, in association with severe crypt hyperplasia, throughout segments of the small intestine in virus-inoculated piglets (Figure 2).

Each of the three live attenuated monovalent vaccines (174-1V-80, PRG942V-80, and K71V-80) and the live attenuated trivalent vaccine (a mixture of strains 174-1V-80, PRG942V-80, and K71V-80) were evaluated at 2 weeks post-vaccination for efficacy in protecting animals from diarrhea induced by challenge with each of the homologous virulent strains or a mixture of three homologous virulent strains. Vaccination with the trivalent vaccine or its individual strains did not induce diarrhea during the first 2 weeks post-vaccination, indicating that these vaccines were safe for use in piglets. Additionally, challenge exposures with each of the homologous virulent strains in its corresponding monovalent vaccine-immunized piglets or with a mixture of the three original virulent strains in trivalent vaccine-immunized piglets did not induce diarrhea for 2 weeks after challenge exposure, indicating that these vaccines were effect in protecting from diarrhea following subsequent exposure to their homologous virulent strain(s) (Table 2).

**Table 2:** **Summary of clinical signs and incidence of fecal virus shedding in colostrum-deprived neonatal piglets immunized with each live attenuated monovalent or trivalent vaccines and then challenged with each corresponding original virulent strain(s) (174-1, PRG942, or K71 strains)**

Vaccine strain	Piglet no.	Vaccination (administrated at 3-day-old)		Challenge (inoculated at 14 days post-vaccination)	
		Occurrence of diarrhea	RT-PCR onset (duration)	Occurrence of diarrhea	RT-PCR onset (duration)
174-1V-80 (G8P[7])	1	None	1 (5)	None	14 (2)
	2	None	1 (4)	None	14 (3)
	3	None	1 (5)	None	14 (3)
	4	None	1 (5)	None	14 (5)
	5	None	1 (6)	None	14 (3)
PRG942V-80 (G9P[23])	6	None	1 (6)	None	14 (3)
	7	None	1 (5)	None	14 (3)
	8	None	1 (5)	None	14 (4)
	9	None	1 (7)	None	14 (5)
	10	None	1 (5)	None	14 (5)
K71V-80 (G5P[7])	11	None	1 (7)	None	14 (5)
	12	None	1 (5)	None	14 (3)
	13	None	1 (5)	None	14 (3)
	14	None	1 (3)	None	14 (4)
	15	None	1 (5)	None	14 (5)
Trivalent vaccine (174-1V-80, PRG942V-80, and K71V-80)	16	None	1 (5)	None	14 (5)
	17	None	1 (6)	None	14 (5)
	18	None	1 (5)	None	14 (7)
	19	None	1 (7)	None	14 (5)
	20	None	1 (7)	None	14 (5)
Mock-control	21	None	None	None	None
	22	None	None	None	None
	23	None	None	None	None
	24	None	None	None	None
	25	None	None	None	None

### Fecal virus shedding

Although vaccinated piglets had no diarrhea for 2 weeks following vaccination, all vaccinated piglets, regardless of monovalent and trivalent vaccine types, exhibited fecal virus shedding for a short period of time. Specifically, 174-1V-80 vaccinated piglets shed viruses for 4–6 days from the 1 day after vaccination, PRG942V-80 vaccinated piglets excreted viruses for 5–7 days from the 1 day after vaccination, K71V-80 vaccinated piglets shed viruses for 3–7 days from the 1 day after vaccination, and trivalent vaccine immunized piglets shed viruses for 5–7 days from the 1 day after vaccination (Table 2). Moreover, all piglets examined after challenge exposure also showed fecal virus shedding for a short period of time, where challenge of 174-1V-80 vaccinated piglets with the original virulent strain 174-1 caused virus shedding for 2–5 days from the first day after challenge exposure, challenge of PRG942V-80 vaccinated piglets with the original virulent strain PRG942 resulted in excreted viruses for 3–5 days from the first day after challenge exposure, challenge of K71V-80 vaccinated piglets with the original virulent strain K71 resulted in virus shedding for 3–5 days from the first day after challenge exposure, and challenge of trivalent vaccine immunized piglets with the three original virulent strains (174-1, PRG942, and K71) caused viral excretion for 5–7 days from the first day after challenge exposure (Table 2). These data imply that the live attenuated monovalent vaccine and the live attenuated trivalent vaccine protected the piglets from diarrhea caused by challenge exposure but did not completely prevent the replication of their corresponding virulent strain(s).

### Rotavirus vaccination decreases histopathologic lesions in the small intestine caused by the virulent RVA strains

Lesions in the small intestines of piglets vaccinated and then challenged with the corresponding original virulent strains or a mixture of the three virulent strains were compared with those in the small intestines of mock-vaccinated, virus-inoculated piglets. Compared to mock-vaccinated, virulent-strain inoculated piglets, the trivalent or the individual-component vaccinated piglets showed a marked decrease in epithelial desquamation, villous atrophy and fusion, and crypt hyperplasia after challenge with corresponding virulent strain(s) (Figure 2). Data derived from histopathological lesion–scores also revealed significant decreases in the vaccinated groups compared with that of the non-vaccinated group (Additional file 4). These data demonstrate that the trivalent and its individual component (monovalent) vaccines alleviated histopathological lesions caused by challenge exposure of each of the corresponding original virulent strain(s).

### VN and ELISA analysis of serum or fecal samples

**Figure 3:**
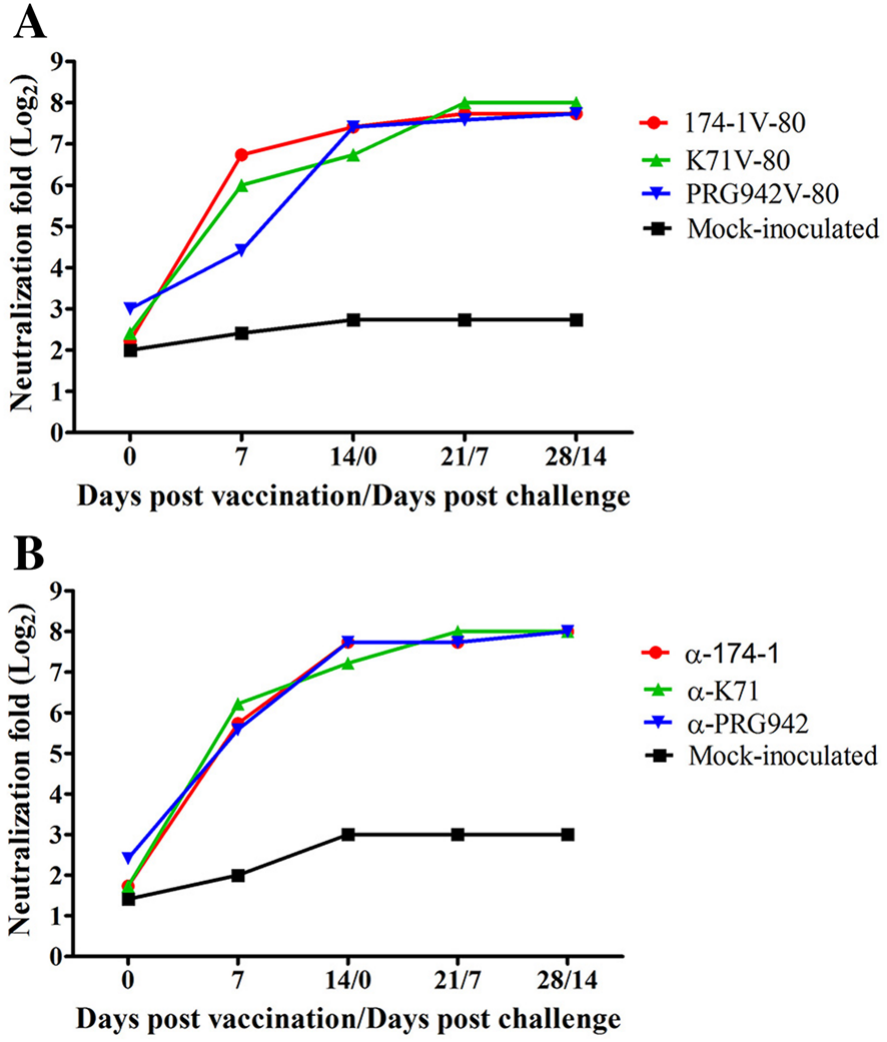
**Serum neutralizing antibody titers in response to the monovalent or trivalent porcine rotavirus vaccination. A** Serum samples obtained weekly from piglets immunized with monovalent 174-1V-80, PRG942V-80, or K71V80 vaccines and challenged by exposure with the corresponding virulent strain at 2 weeks post-vaccination. **B** Serum samples obtained weekly from piglets immunized with a trivalent porcine live attenuated rotavirus vaccine and challenged by exposure with its virulent strain 174-1 at 2 weeks post-vaccination. Serum neutralizing antibody titers were calculated as the geometric mean titers for each group (*n* = 5 per group).

VN antibody titers were sequentially evaluated using the serum samples obtained from the experimental animals. All 3 groups of 5 piglets which were vaccinated with each monovalent vaccine and then challenged with each original strain produced a VN antibody titer increase ofat least 24-folds at 7 dpv, which then reached over 27-folds at 14 dpv, and sustained high VN antibody levels until the end of experiments (Figure 3A). Additionally, piglets immunized with a trivalent vaccine and then challenge-exposed with a mixture of three virulent strains showed a similar pattern of VN antibody titers to each original virulent strain (Figure 3B). These results indicated that oral immunization with each monovalent vaccine or a trivalent vaccine induces serum VN antibody levels from 1-week post-vaccination, and these levels remain high after challenge exposure with virulent strain(s).

**Figure 4:**
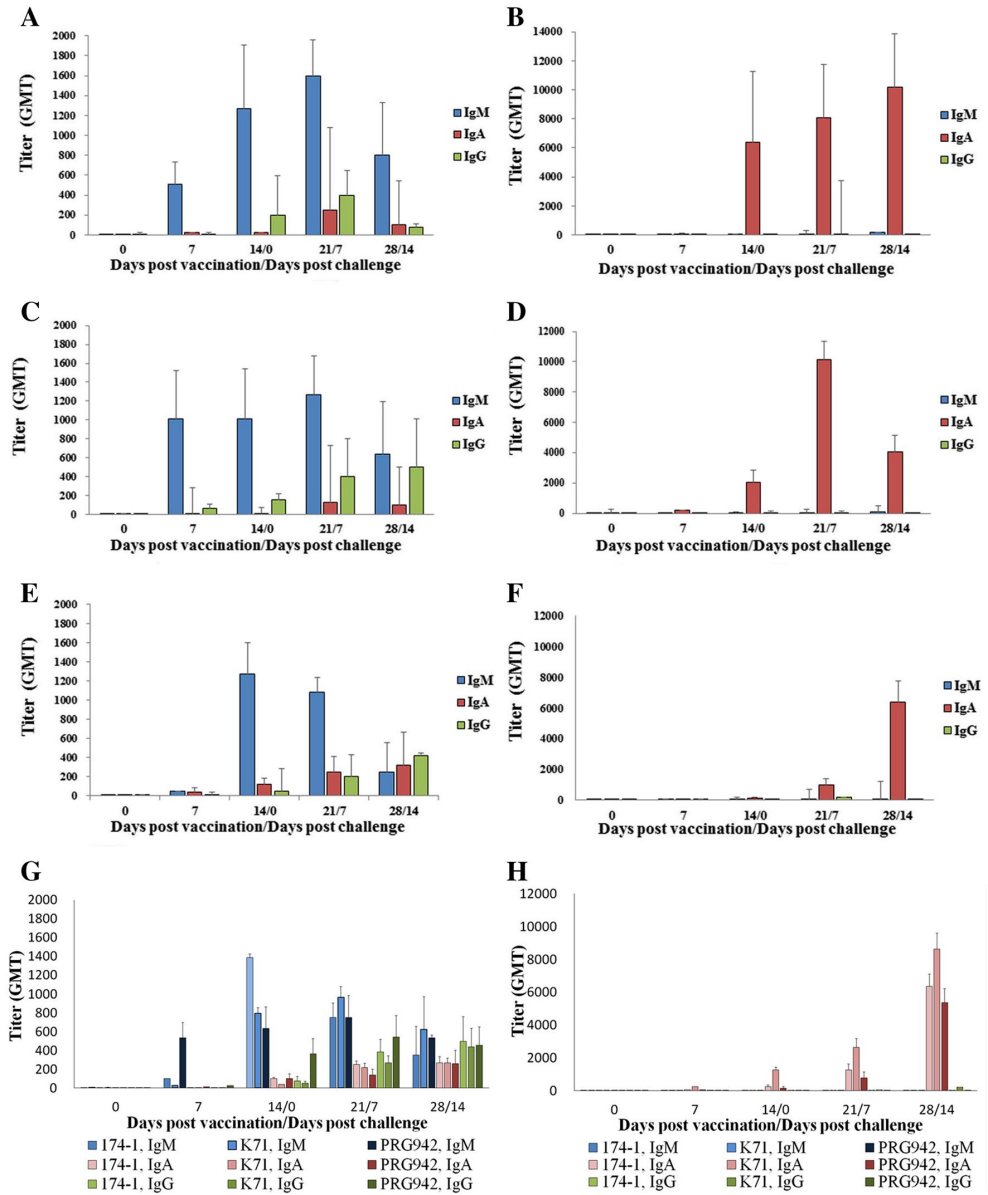
**Serum and fecal RV-specific IgM, IgG, and IgA antibody titers determined by ELISA. A**, **B** Serum and fecal samples obtained weekly from piglets immunized with a live attenuated strain 174-1V-80 and challenged by exposure with its virulent strain 174-1 at 2 weeks post-vaccination. **C**, **D** Serum and fecal samples obtained weekly from piglets immunized with a live attenuated strain PRG942V-80 and challenged by exposure with its virulent strain PRG942 at 2 weeks post-vaccination. **E**, **F** Serum and fecal samples obtained weekly from piglets immunized with a live attenuated strain K71V-80 and challenged by exposure with its virulent strain K71 at 2 weeks post-vaccination. **G**, **H** Serum and fecal samples obtained weekly from piglets immunized with a live attenuated trivalent vaccine containing live attenuated strains 174-1V-80, PRG942V-80, and K71V-80 and challenged by exposure with its virulent strains 174-1, PRG942, and K71 at 2 weeks post-vaccination. Antibody titers are expressed as the geometric mean titers (GMTs) for each group (*n* = 5 piglets per each group).

To detect RVA-specific IgM, IgG, and IgA antibodies in the serum samples, ELISAs were performed using serum samples collected weekly from the experimental groups. Results showed that RVA-specific IgM antibodies were detectable beginning at 7 dpv, peaked at 14 dpv (PRG942V-80) or 21 dpv (174-1V-80 and K71V-80) with titers up to 10^3.1^, and then gradually decreased (Figure 4). Serum IgG levels were induced from 14 dpv, and they continuously increased until the end of the experiments, where they reached titers of 10^1.9^ (174-1V-80), 10^2.6^ (PRG942V-80), and 10^2.7^ (K71V-80). The trivalent vaccine induced serum IgG levels of 10^2.72^ against strain 174-1, 10^2.4^ against strain PRG942, and 10^2.66^ against strain K71 at 28 dpv. In contrast to IgM or IgG antibodies, serum RVA–specific IgA antibody levels increased slightly from 14 dpv until the end of the experiments, reaching titers up to 10^2.0–2.5^ (Figure 4). All three vaccine strains induced a seroconversion in serum samples from IgM to IgG at 14 dpv following challenge exposures.

The RVA-specific IgM, IgG, and IgA antibodies found in the fecal samples from the experimental groups were analyzed by ELISA. Secretory fecal IgA antibodies specific for each vaccine strain were detected from 14 dpv or 21 dpv, regardless of monovalent or trivalent vaccine, and these levels gradually increased through 28 dpv. The levels of IgM and IgG antibodies in the fecal samples from the experimental groups were very low (Figure 4). These data indicate that each of the monovalent vaccines and the trivalent vaccine induced secretory IgA antibodies at levels capable of protecting the piglets from diarrhea induced by the homologous virulent strain(s).

### Safety test

The safety of each of the three live attenuated porcine RVA vaccine strains was examined according to the guidelines of the Animal and Plant Quarantine Agency, Republic of Korea. Each vaccine strain was inoculated into mice by IP injection, guinea pigs by both IM or IP injections, and pigs by IM injection. None of the three strains induced any clinical signs of infection, including diarrhea or mortality (Additional file 5). These results demonstrate that each of the vaccine strains is safe at least for use in pigs.

### Virulence reversion

To determine whether consecutive oral inoculations of each vaccine strain in piglets would restore virulence, a virulence reversion test was performed using three-day-old colostrum-deprived piglets as previously described [31]. No abnormal clinical signs, including diarrhea or mortality, were observed in any of the experimental piglets during five consecutive passages of each of the live attenuated vaccine strains (Table 3). Histopathologic evaluation of experimental animals inoculated with each of the vaccine strains or filtered supernatant of a mixture of homogenized small intestine and its fecal content from each round of passage revealed that none of the animals showed any histopathologic changes (Additional files 14, 15, 16, 17, 18, 19). These data demonstrate that each of the vaccine strains was safe for use in neonatal piglets.

**Table 3:** **Clinical signs of experimental piglets administrated with each vaccine strain (174-1V-80, PRG942V-80, and K71V-80) or filtered supernatant of a mixture of homogenized small intestine and fecal contents sampled from each passage**

Vaccine strain	Route of administration	No. of animals in each passage	Clinical signs (No. of diarrheic animals/No. of animals used)				
			1^st^ passage	2^nd^ passage	3^rd^ passage	4^th^ passage	5^th^ passage
174-1V-80	Oral route	2	0/2	0/2	0/2	0/2	0/2
PRG942V-80	Oral route	2	0/2	0/2	0/2	0/2	0/2
K71V-80	Oral route	2	0/2	0/2	0/2	0/2	0/2

## Discussion

As the list of G and P genotypes for RVA expands, the diversity of RVAs infecting domestic animals also rapidly increases [10]. Moreover, prevalent G and P genotypes of RVAs, including porcine and bovine RVAs, can change depending on the time or geographical location [10, 12]. Therefore, G- and P-genotypes in RVA vaccines should match the currently circulating filed strains. In South Korea, discrepancies between the predominant field G- and P-genotypes of porcine RVAs and those of the vaccine strains have resulted in huge economic losses in the swine industry [15]. Our previous study showed that G- and P-genotype combinations detected in Korean porcine RVA strains included G5P[7], G8P[7], G9P[7], G9P[23], and G8P[1] [15]. These data indicate that Korean porcine RVA strains of G8P[7], G9P[7], G9P[23], and G8P[1] genotypes could not be protected against by imported and domestic porcine RVA vaccines [15]. In response to strong requests from Korean pig farmers and veterinarians, an updated live attenuated porcine RVA vaccine was developed in the current study to protect against not only the globally most prevalent G5P[7] strain but also the unique Korean strains G8P[7] and G9P[23]. The trivalent RVA vaccine described here should provide improved control of porcine RVA infections and disease in South Korea and other countries where RVA infections with similar G- and P-genotypes included in this vaccine are problematic.

RVA vaccines should be able to prevent diarrhea, the major RVA-induced symptom [10, 31]. In the current study, piglets immunized with each of the live attenuated monovalent vaccines or with the live attenuated trivalent vaccine were protected from diarrhea upon challenge exposure with the homologous virulent strain(s). Moreover, immunization of the piglets with each of the monovalent or trivalent vaccines markedly reduced the development of histopathological lesions in the small intestines after challenge exposure to the homologous original virulent strain(s). These data demonstrate that each of the live attenuated monovalent vaccines and the trivalent vaccine was highly effective at protecting the piglets from diarrhea and intestinal lesions typically induced by the corresponding virulent strain(s).

As against other enteric viruses, secretory IgA (SIgA) acts as the first line of defense in protecting the intestinal epithelium from enteric pathogens [14, 32, 33]. Therefore, immunization of piglets with RVA vaccines should induce the production and secretion of SIgA into the lumen of the small intestine to protect the host from incoming virulent RVAs. The results from the current study suggest that as each of the live attenuated monovalent vaccines and the trivalent vaccine protected the piglets from diarrhea and development of lesions in small intestine by challenge exposure to the homologous virulent strain(s), the SIgA detected in the fecal samples specific for each of the vaccine strains was induced and secreted into the intestinal lumen at amounts sufficient to provide protection [32, 33]. Effectiveness of RVA vaccines may be influenced by other factors such as malnutrition, intestinal microbiome, co-infection of the gut, immunological immaturity, maternal RVA-specific antibodies, and genetic factors [34–44]. These characteristics may be evident in low-income settings compared to that in high-income settings, i.e., high levels of vaccine efficacy have been reported for both human monovalent Rotarix and pentavalent Rotateq vaccines in high-income settings of Hong Kong, Singapore, and Japan, while lower and more variable levels of protection have been reported in low-income settings in sub-Saharan Africa and Asia [34, 36, 40]. As these factors have not been determined in livestock farms, future studies should address this.

Beyond the general belief that RVAs are able to only infect the small intestinal mucosa to induce pathology, there is accumulating evidence that RVAs may spread to extra-intestinal organs and tissues in infected humans and animals via a viremia, resulting in systemic symptoms and nongastroenteric clinical diseases including respiratory illness and neurological syndromes [45–49]. In the present study, piglets orally immunized with each of the live attenuated monovalent vaccines or the trivalent vaccine generated strong serum VN antibody titers and increased IgM and IgG levels to each of the corresponding RVA strains. These data suggest that oral immunization of piglets with the live attenuated monovalent vaccines or the trivalent vaccine may protect the piglets from viremia and the extra-intestinal lesions caused by virulent RVA infection. Indeed, the virulent RVA strains G5P[7] K71, G8P[7] 174-1, and G9P[23] are reported to induce both intestinal and extra-intestinal lesions in experimentally infected piglets [16, 22, 29]. Therefore, an ongoing study is investigating whether immunization of piglets with these vaccines will protect not only from diarrhea and intestinal lesions, but also from viremia and extra-intestinal lesions as well.

It has been observed or speculated that the RVA genome segments VP4, VP7, VP3, NSP1, NSP2, and NSP4 may be involved in RVA virulence and/or host range restriction [16, 17, 23, 29, 50–55]. Comparing the 80^th^ passage of each vaccine strain with their original virulent strains, there were several substitutions of deduced amino acids within these suspected genomic segments and others. Based on these data, it is difficult to specifically identify which amino acid(s) in the particular genomic segment(s) are related to RVA virulence. Therefore, the molecular and biological impact of each amino acid change on RVA virulence should be addressed in future studies. This could be achieved by using several sophisticated methods such as recently developed reverse genetics to swap individual genes of these virulent and attenuated strains or to introduce particular amino acid substitutions [56, 57].

The interspecies transmission of RVAs, either as whole virions or as reassortant virions, appears to occur in nature [58–63]. In South Korea, many porcine and bovine RVAs have been reported as reassortant viruses, possibly due to interspecies transmission [15, 18]. For example, bovine reassortant strains carrying the bovine VP7-G8 and porcine VP4-P [7] genotype are ranked as the most frequently isolated strains from the fecal samples of diarrheic calves [16–18, 20, 23, 29, 57], whereas porcine reassortant G8P [7] strains are ranked as the second most frequently detected G and P genotypes of the Korean porcine RVA strains isolated from the fecal samples of diarrheic piglets [15]. In order to protect from the G8-bearing RVA strains on pig farms and to prevent its spread back to cow farms, strain 174-1V-80 was chosen as one of the current vaccine strains since it contains two bovine genotypes (VP7-G8 and VP3-M2) with the remaining nine genotypes being of porcine origin [15, 29]. In addition, G5P[7] strain K71V-80 may prevent spread of its corresponding virulent strain to cow farms, as G5P[7] carrying bovine RVAs are ranked the second most prevalent in calf diarrhea in South Korea [16–18, 20, 23, 29, 57]. Porcine G9 RVA strains are ranked as the third most prevalent cause of diarrhea in pigs [15]. Additionally, human G9 RVA strains have been identified as the fifth most important RVA genotype globally [62], and these were the most important genotype in South Korea from 2007 to 2009 [63]. As pigs and humans are the only species from which G9 RVA strains have been detected, pigs are suspected to be potential host reservoirs for human RVAs [15, 17, 62]. The current G9P [23] PRG942 vaccine strain may be protective in preventing the occurrence of G9P [23] strains in pigs on farms and may prevent interspecies transmission between pigs and humans.

While efficacy is a critical concern, safety is equally important for vaccines. In particular, live attenuated RVA vaccines should not cause any clinical signs such as vaccine-induced diarrhea. Therefore, the Animal and Plant Quarantine Agency, Republic of Korea, requires data regarding the safety of porcine live attenuated RVA vaccines in mice, guinea pigs, and pigs. In the current study, none of the live attenuated monovalent vaccines nor the live attenuate trivalent vaccine induced any clinical signs including diarrhea in the immunized neonatal piglets, indicating that these vaccine strains were safe for use in the neonatal piglets. Moreover, these vaccines did not induce any clinical signs in immunized mice, guinea pigs, or pigs, and none of the live attenuated vaccine strains caused diarrhea in any experimental piglets during five consecutive passages, further fulfilling the requirements of the Animal and Plant Quarantine Agency, Republic of Korea.

In conclusion, each of the live attenuated monovalent vaccines and the live attenuated trivalent vaccine protected against diarrhea and alleviated small intestinal histopathological lesions in immunized piglets challenged with exposure by homologous virulent strains. The protection appeared to be provided through the induction of SIgA into the small intestine. Moreover, these live attenuated vaccines activated RVA-specific serum VN antibody titers and induced increased levels of serum IgM and IgG, possibly protecting the vaccinated animals from viremia and extra-intestinal lesions. In addition, these live attenuated vaccines were safe for use in piglets and demonstrated no reversion in virulence during five consecutive passages in piglets. Further studies are required to demonstrate whether each of the monovalent vaccines and the trivalent vaccine may also provide protective effects against RVA strains carrying other G and P genotypes. This new trivalent vaccine will likely contribute toward controlling porcine RVA infections in South Korea and other countries in which RVA infections involving these G-and P-genotype are problematic.

## Additional files

**Additional file 1.** **Comparison of genotype constellation of porcine 174-1 (G8P[7]), PRG942 (G9P[23]), and K71 (G5P[7]) strains with other known reference genotypes.**

**Additional file 2.** **Oligonucleotide primers for sequencing or for 5′ and 3′ RACE PCRs of all eleven genomic segments of the porcine 174-1, PRG942, and K71 strains and their passages.** Listed in the table are the primer pairs used to generate the full-length sequence of Korean porcine 174-1, PRG942, and K71 rotavirus strains. Also indicated are the gene-specific primers used for 5′ and 3′ RACE PCR.

**Additional file 3.** **Full-length ORF nucleotide sequence identities (%) of ORF**^**a**^ **in 11 genomic segments between three Korean porcine 174-1, PRG942, and K71 strains and other known strains representative of corresponding or neighbor genotypes.** The nucleotide sequences of open reading frame of the virulent and attenuated porcine 174-1, PRG942, and K71 rotavirus strains were compared with known RVA strains. The values represent the nucleotide similarity of porcine 174-1, PRG942, and K71with the reference strains.

**Additional file 4.** **Summary of the histopathological findings in the small intestine of the colostrums-deprived neonatal piglets inoculated with each virulent strains (174-1, PRG942, and K71), or immunized with each live attenuated monovalent or trivalent vaccines and then challenged with each corresponding original virulent strain(s).**

**Additional file 5.** **Summary of safety test results for a live attenuated porcine rotavirus monovalent vaccine strains 174-1V-80, PRG942V-80, and K71V-80 in mice, guinea pigs, and pigs.**

**Additional file 6.** **Virus titers of each strain in different passages.**

**Additional file 7.** **Phylogenetic trees based on full-length ORF nucleotide sequences of the VP7, VP4, and VP6 gene segments of RVA strains 174-1, K71, and PRG942.** Phylogenetic trees were constructed using the maximum likelihood method based on General Time Reversible (GTR) with gamma distributed substitution model with 500 bootstrap replicates by MEGA 6 software [26]. The GenBank accession numbers for each of the reference genes are listed in Additional file 3. The following data are provided to explain each strain: Serotype of rotavirus/species of origin-virus type/country/strain name/isolation year/G and P genotype is indicated. The serial passage of the porcine vaccine strains is represented by closed circles.

**Additional file 8.** **Phylogenetic trees based on full-length ORF nucleotide sequences of the VP1, VP2, and VP3 gene segments of RVA strains 174-1, K71, and PRG942.** Phylogenetic trees were constructed using the maximum likelihood method based on General Time Reversible (GTR) with gamma distributed substitution model with 500 bootstrap replicates by MEGA 6 software [26]. The GenBank accession numbers for each of the reference genes are listed in Additional file 3. The following data are provided to explain each strain: Serotype of rotavirus/species of origin-virus type/country/strain name/isolation year/G- and P-genotype is indicated. The serial passage of the porcine vaccine strains is represented by closed circles.

**Additional file 9.** **Phylogenetic trees based on full-length ORF nucleotide sequences of the NSP1, NSP2 and NSP3 gene segments of RVA strains 174-1, K71, and PRG942.** Phylogenetic trees were constructed using the maximum likelihood method based on General Time Reversible (GTR) with gamma distributed substitution model with 500 bootstrap replicates by MEGA 6 software [26]. The GenBank accession number for each of the reference genes are listed in Additional file 3. The following data are provided to explain each strain: Serotype of rotavirus/species of origin-virus type/country/strain name/isolation year/G- and P-genotype is indicated. The serial passage of the porcine vaccine strains is represented by closed circles.

**Additional file 10.** **Phylogenetic trees based on full-length ORF nucleotide sequences of the NSP4 and NSP5 gene segments of RVAA strains 174-1, K71, and PRG942.** Phylogenetic trees were constructed using the neighbor-joining method based on Kimura-2 (NSP4) or maximum likelihood method based General Time Reversible (GTR) (NSP5) with gamma distributed substitution model with 500 bootstrap replicates by MEGA 6 software [26]. The GenBank accession number for each of the reference genes are listed in Additional file 3. The following data are provided to explain each strain: Serotype of rotavirus/species of origin-virus type/country/strain name/isolation year/G- and P-genotype is indicated. The serial passage of the porcine vaccine strains is represented by closed circles.

**Additional file 11.** **Comparison of full-length amino acid sequences of 11 genomic segments of 174-1V-80 (G8P[7]) vaccine strain with its different passages.** The full-length amino acid sequences of the 11 genomic segments of the 80^th^-passage attenuated 174-1V-80 vaccine strain was compared with those of the 20^th^-, 40^th^-, and 60^th^-passage attenuated strains and the original virulent strain.

**Additional file 12.** **Comparison of full-length amino acid sequences of 11 genomic segments of PRG942V-80 (G9P[23]) vaccine strain with its different passages.** The full-length amino acid sequences of the 11 genomic segments of the 80^th^-passage attenuated PRG942V-80 vaccine strain was compared with those of the 20^th^-, 40^th^-, and 60^th^-passage attenuated strains and the original virulent strain.

**Additional file 13.** **Comparison of full-length amino acid sequences of 11 genomic segments of K71V-80 (G9P[23]) vaccine strain with its different passages.** The full-length amino acid sequences of the 11 genomic segments of the 80^th^-passage attenuated K71V-80 vaccine strain was compared with those of the 20^th^-, 40^th^-, and 60^th^-passage attenuated strains and the original virulent strain.

**Additional file 14.** **Summary of the histopathological findings in the small intestine of the colostrums-deprived neonatal piglets vaccinated with a live attenuated monovalent vaccine strain (174-1V-80) or filtered supernatant of a mixture of homogenized small intestine and feces sampled from each passage.**

**Additional file 15.** **Summary of the histopathological findings in the small intestine of the colostrums-deprived neonatal piglets vaccinated with a live attenuated monovalent vaccine strain (PRG942V-80) or filtered supernatant of a mixture of homogenized small intestine and feces sampled from each passage.**

**Additional file 16.** **Summary of the histopathological findings in the small intestine of the colostrums-deprived neonatal piglets vaccinated with a live attenuated monovalent vaccine strain (K71V-80) or filtered supernatant of a mixture of homogenized small intestine and feces sampled from each passage.**

**Additional file 17.** **Histopathological changes in the small intestines of piglets inoculated with a porcine live attenuated monovalent rotavirus strain (174-1V-80) and its serial passages.** (A–E) Duodenum sampled from piglets inoculated with each of the serial passaged viruses demonstrated normal long slender villi and short crypts in the mucosal membrane. (F–J) Jejunum sampled from piglets inoculated with each of the serial passaged viruses demonstrated normal long slender villi and short crypts in the mucosal membrane. (K–O) Ileum sampled from piglets inoculated with each of the serial passaged viruses demonstrated normal long slender villi and short crypts in the mucosal membrane. Bar = 200 μm.

**Additional file 18.** **Histopathological changes in the small intestine of piglets inoculated with a porcine live attenuated monovalent rotavirus strain (PRG942V-80) and its serial passages.** (A–E) Duodenum sampled from piglets inoculated with each of the serial passaged viruses demonstrated normal long slender villi and short crypts in the mucosal membrane. (F–J) Jejunum sampled from piglets inoculated with each of the serial passaged viruses demonstrated normal long slender villi and short crypts in the mucosal membrane. (K–O) Ileum sampled from piglets inoculated with each of the serial passaged viruses demonstrated normal long slender villi and short crypts in the mucosal membrane. Bar = 200 μm.

**Additional file 19.** **Histopathological changes in the small intestine of piglets inoculated with a porcine live attenuated monovalent rotavirus strain (K71V-80) and its serial passages.** (A–E) Duodenum sampled from piglets inoculated with each of the serial passaged viruses demonstrated normal long slender villi and short crypts in the mucosal membrane. (F–J) Jejunum sampled from piglets inoculated with each of the serial passaged viruses demonstrated normal long slender villi and short crypts in the mucosal membrane. (K–O) Ileum sampled from piglets inoculated with each of the serial passaged viruses demonstrated normal long slender villi and short crypts in the mucosal membrane. Bar = 200 μm.

## Abbreviations

BSA: bovine serum albumin
CCIF: cell culture immunofluorescence
DD_50_: median diarrheic dose
dpi: days post-inoculation
dpv: days post-vaccination
ds: double-stranded
ELISA: enzyme-linked immunosorbent assays
FBS: fetal bovine serum
FFU: fluorescence focus units
FITC: fluorescein isothiocyanate
IM: intramuscular
IP: intraperitoneal
OD: optical density
ORF: open-reading frames
PAGE: polyacrylamide gel electrophoresis
PBS: phosphate buffered saline
RT-PCR: reverse transcription-polymerase chain reaction
RVA: group A rotavirus
SIgA: secretory IgA
VN: virus neutralization
