# Supplementary material for: Development of a live attenuated trivalent porcine rotavirus A vaccine against disease caused by recent strains most prevalent in South Korea
Source: Vet Res. 2019 Jan 7;50:2. doi: 10.1186/s13567-018-0619-6 (PMC6323864; doi:10.1186/s13567-018-0619-6)
Supplement: Supplementary file 1 — Additional file 1. Comparison of genotype constellation of porcine 174-1 (G8P[7]), PRG942 (G9P[23]), and K71 (G5P[7]) strains with other known reference genotypes. [file 13567_2018_619_MOESM1_ESM.docx]

**Additional file 1 Comparison of genotype constellation of porcine 174-1 (G8P[7]), PRG942 (G9P[23]), and K71 (G5P[7]) strains with other known reference genotypes**

| Strain^a^ | Genotype constellation^b^ | | | | | | | | | | |
| --- | --- | --- | --- | --- | --- | --- | --- | --- | --- | --- | --- |
|  | VP7 | VP4 | VP6 | VP1 | VP2 | VP3 | NSP1 | NSP2 | NSP3 | NSP4 | NSP5 |
| **RVA/Pig-tc/KOR/174-1/2006/G8P[7]** | **G8** | **P[7]** | **I5** | **R1** | **C1** | **M2** | **A1** | **N1** | **T1** | **E1** | **H1** |
| **RVA/Pig-tc/KOR/K71/2006/G5P[7]** | **G5** | **P[7]** | **I5** | **R1** | **C1** | **M1** | **A1** | **N1** | **T1** | **E1** | **H1** |
| **RVA/Pig-tc/KOR/PRG942/2006/G9P[23]** | **G9** | **P[23]** | **I5** | **R1** | **C1** | **M1** | **A8** | **N1** | **T1** | **E1** | **H1** |
| RVA/Pig-tc/KOR/156-1/2006/G8P[7] | G8 | P[7] | I5 | R1 | C1 | M2 | A1 | N1 | T1 | E1 | H1 |
| RVA/Pig-tc/KOR/C-1/2006/G8P[7] | G8 | P[7] | I5 | R1 | C1 | M2 | A8 | N1 | T1 | E1 | H1 |
| RVA/Cow-tc/KOR/KJ56-1/2004/G8P[7] | G8 | P[7] | I5 | R1 | C1 | M2 | A1 | N1 | T1 | E1 | H1 |
| RVA/Pig-tc/KOR/PRG9121/2006/G9P[7] | G9 | P[7] | I5 | R1 | C1 | M1 | A8 | N1 | T1 | E1 | H1 |
| RVA/Pig-tc/MEX/YM/1983/G11P9[7] | G11 | P[7] | I5 | R1 | C1 | M1 | A8 | N1 | T1 | E1 | H1 |
| RVA/Panda-xx/CHN/CH-1/2008/G1P[7] | G1 | P[7] | I5 | R1 | C1 | M1 | A1 | N1 | T1 | E1 | H1 |
| RVA/Pig-tc/USA/OSU/1977/G5P9[7] | G5 | P[7] | I5 | R1 | C1 | M1 | A1 | N1 | T1 | E1 | H1 |
| RVA/Pig-tc/VEN/A131/1988/G3P9[7] | G3 | P[7] | I5 | R1 | C2 | M1 | A1 | N1 | T1 | E1 | H1 |
| RVA/Pig-tc/VEN/A253/1988/G11P9[7] | G11 | P[7] | I5 | R1 | C2 | M1 | A1 | N1 | T1 | E1 | H1 |
| RVA/Human-tc/USA/Wa/1974/G1P1A[8] | G1 | P[8] | I1 | R1 | C1 | M1 | A1 | N1 | T1 | E1 | H1 |
| RVA/Pig-tc/USA/Gottfried/1983/G4P[6] | G4 | P[6] | I1 | R1 | C1 | M1 | A8 | N1 | T1 | E1 | H1 |
| RVA/Cow-tc/Japan /Niigata9801/1998/G8P[14] | G8 | P[14] | Ix | Rx | Cx | Mx | Ax | Nx | Tx | Ex | Hx |
| RVA/Human-tc/COD /DRC86/2003/G8P[6] | G8 | P[6] | I2 | R2 | C2 | M2 | A2 | N2 | T2 | E2 | H2 |
| RVA/Human-tc/USA/DS-1/1976/G2P1B[4] | G2 | P[4] | I2 | R2 | C2 | M2 | A2 | N2 | T2 | E2 | H2 |

^a^ Strains used in this study are written in bold letters.

^b^ Purple and blue represent typical bovine (VP7) and porcine (VP7, VP4, VP6 and NSP1) genotypes, respectively. Bright green represents the Wa-like (human/porcine) genotypes, whereas dark green represents different clusters inside the Wa-like genotypes. Red is for the DS-1 like (human/bovine genotypes) genotypes. X indicates the unknown genotype.
