# Supplementary material for: Development of a live attenuated trivalent porcine rotavirus A vaccine against disease caused by recent strains most prevalent in South Korea
Source: Vet Res. 2019 Jan 7;50:2. doi: 10.1186/s13567-018-0619-6 (PMC6323864; doi:10.1186/s13567-018-0619-6)
Supplement: Supplementary file 2 — Additional file 2. Oligonucleotide primers for sequencing or for 5′ and 3′ RACE PCRs of all eleven genomic segments of the porcine 174-1, PRG942, and K71 strains and their passages. Listed in the table are the primer pairs used to generate the full-length sequence of Korean porcine 174-1, PRG942, and K71 rotavirus strains. Also indicated are the gene-specific primers used for 5′ and 3′ RACE PCR. [file 13567_2018_619_MOESM2_ESM.docx]

**Additional file 2 Oligonucleotide primers for sequencing or for 5’ and 3’ RACE PCRs of all eleven genomic segments of the porcine 174-1, PRG942, and K71 strains and their passages.**

| Target  gene^a^ | Primer name | Sequence (5’-3’) ^b^ | Region  (nt) | Size  (bp) | Ref |
| --- | --- | --- | --- | --- | --- |
| VP1 | GEN-VP1F | F:GGCTATTAAAGCTRTACAATGGGGAAG | 1–27 | 1592 | [6] |
|  |  | R:TCC CAC TGG GAM ACG TCT GTA TAT | 1569–1592 |  | [18] |
|  |  | F:GAATTCTAC TCACAGTCAA AT | 1492–1512 | 1811 | [18] |
|  |  | R:GGTCACATCTAAGCGCTCTAATCTTS | 3281–3302 |  | [18] |
|  | VP1-5’ ^b^ | R:CGAGTTCATTCGCCGTCAAATCTGCTTC | 340-313 | 340 | [16] |
|  | VP1-3’ ^c^ | F: TTTCACTAGGAGTCCCACCAGTTGATGC | 2906-2933 | 396 | [16] |
| VP2 |  | F:GGCTATTRAAGGYTCAATGGCGTACAG | 1–27 | 1393 | [18] |
|  |  | R:TTTCTATAATGCATTCKTTGCATT | 1375–1393 |  | [18] |
|  |  | F:ATAAAYTCACA AGCAGCAAAT GA | 1160–1182 | 1558 | [18] |
|  | GEN-VP2_Rbc | R:GTCATATCTCCACARTGGGGTTGG | 2666–2689 |  | [6] |
|  | VP2-5’ ^b^ | R: CATAATCTCCATCTGGCAGCGTGTCTCT | 521-494 | 521 | [16] |
|  | VP2-3’ ^c^ | F:AGTGTACAAGCAGGTACCGCAACCCTTT | 2490-2517 | 200 | [29] |
| VP3 |  | F:GGCTTTTTAAAGCAATATTAGTA | 1–23 | 970 | [18] |
|  |  | R:ATGGTGTGTCCAATGGATCC | 970–989 |  | [18] |
|  |  | F:GGATCCATTGGACACACCAT | 970–989 | 1603 | [18] |
|  |  | R:GGTCACGACCTGACCATGGTG | 2576–2596 |  | [18] |
|  | VP3-5’ ^b^ | R:GCCCTCTTGTGTCATTCCCGTTATATCC | 459-432 | 459 | [29] |
|  | VP3-3’ ^c^ | F:CACGCTCCGATAGAGTTGATTTACGC | 2036-2061 | 595 | [29] |
| VP4 | GEN-VP4F | F:GGCTATAAAATGGCTTCGCTCA | 1–22 | 868 | [6] |
|  |  | R:ATTTCGGACCATTTATAA CC | 868–887 |  | [64] |
|  |  | F:GGT TAT AAA TGG TCC GAA AT | 868–887 | 1477 | [18] |
|  |  | R:GGYCWCAACCTCTAGACACT | 2343–2362 |  | [18] |
|  | VP4-5’ ^b^ | R:CCCTCTACAGTTGGCGCAAGTAGTACCC- | 278-251 | 278 | [16] |
|  | VP4-3’ ^c^ | F:CAATAGGATCATCAGCATCCGCTTGGAC | 1757-1784 | 605 | [16] |
| VP6 | GEN-VP6F | F:GGCTTTWAAACGAAGTCTTC | 1–20 | 1356 | [6] |
|  | GEN-VP6R | R:GGTCACATCCTCTCACT | 1340–1356 |  | [6] |
|  | VP6-5’ ^b^ | R:GAGCTATTCCGTCTCGTTGCGACTCTCT | 354-327 | 354 | [29] |
|  | VP6-3’ ^c^ | F:GTGTTCCCACCAGGTATGAATTGGACAG | 1101-1128 | 255 | [16] |
| VP7 |  | F:GCCTTTAAAAGCGAGAATTT | 1–20 | 1062 | [65] |
|  |  | R:GGTCACATCATACAACTCTA | 1043–1062 |  | [65] |
|  | VP7-5’ ^b^ | R:GACAGAACCAGTTGGCCATCCTTTTG | 396-371 | 396 | [29] |
|  | VP7-3’ ^c^ | F:GGCACCACAGACAGAAAGGATGATGAG | 879-905 | 183 | [29] |
| NSP1 | LAP-NSP1-F | F:GGGCTTTTTTTTGAAAAGTC | 1–20 | 1567 | [60] |
|  | VF5R | R:GGTCACATTTTATGCTGCCTA | 1547–1567 |  | [66] |
|  | NSP1-5’ ^b^ | R:CATGATACATGGTACAGCCTCGACAG | 215-190 | 215 | [16] |
|  | NSP1-3’ ^c^ | F:GCCACTGAGGTACACAACTGCAAATGG | 974-1000 | 592 | [16] |
| NSP2 | VF3F | F:GGCTTTTAAAGCGTCTCAGTC | 1–21 | 1058 | [66] |
|  | VF3R | R:GGTCACATAAGCGCTTTCTATTC | 1036–1058 |  | [66] |
|  | NSP2-5’ ^b^ | R:CTTCAGCAGTGGCAGTGGTTTCAATTTC | 506-479 | 506 | [16] |
|  | NSP2-3’ ^c^ | F:CACGCAGACAGAGTATTCGCTACA | 755-778 | 304 | [16] |
| NSP3 | VF2F | F:ATGCTCAAGATGGAGTCTACT | 1–21 | 1050 | [66] |
|  | VF2R | R:GGTCACATAACGCCCCTATAG | 1030–1050 |  | [66] |
|  | NSP3-5’ ^b^ | R:AGTGCCTGATCAATAGTCGCAGCTTTGC | 246-219 | 246 | [16] |
|  | NSP3-3’ ^c^ | F:GAGGTCCATGGAATTGTCAGATGATGTC | 772-799 | 303 | [16] |
| NSP4 | 10Beg16 | F:TGTTCCGAGAGAGCGCGTG | 16–34 | 725 | [67] |
|  | 10End722c | R:GACCATTCCTTCCATTAAC | 722–740 |  | [67] |
|  | NSP4-5’ ^b^ | R:CGAACACTTCGACGTTCTCAACGCTATT | 233-206 | 233 | [29] |
|  | NSP4-3’ ^c^ | F:CTATGTGAGAGGTTGAGTTGCCGTCGTC | 562-589 | 189 | [29] |
| NSP5 | VF1F | F:GGCTTTTAAAGCGCTACAGTG | 1–21 | 664 | [66] |
|  | VF1R | R:GGTCACAAAACGGGAGTGGG | 645–664 |  | [66] |
|  | NSP5-5’ ^b^ | R:GATCGCACCCAACGTTACTTGAAGGTC | 340-314 | 340 | [29] |
|  | NSP5-3’ ^c^ | F:GTGCGATCAAGTGGATTTCTCCCTGACT | 333-360 | 331 | [29] |
|  | **3’-CDS Primer A** | AAGCAGTGGTATCAACGCAGAGTAC(T)30 V N |  |  | Clontech |

^a^ F, forward; R, reverse.

^b^ Primer for 5’ RACE PCR.

^c^ Primer for 3’ RACE PCR.

**Additional references**

1. Gentsch JR, Glass RI, Woods P, Gouvea V, Gorziglia M, Flores J, Das BK, Bhan MK (1992) Identification of group A rotavirus gene 4 types by polymerase chain reaction. J Clin Microbiol 30:1365-1373
2. Kang SY, Jeon SJ, Chang KO, Park YH, Kim WY (1997) Cloning and nucleotide sequence analysis of VP7 genes of bovine rotaviruses insolated Korea. Korean J Vet Res 37:367–374
3. Varghese V, Das S, Singh NB, Kojima K, Bhattacharya SK, Krishnan T, Kobayashi N, Naik TN (2004) Molecular characterization of a human rotavirus reveals porcine characteristics in most of the genes including VP6 and NSP4. Arch Virol 149:155–172
4. Lee CN, Wang YL, Kao CL, Zao CL, Lee CY, Chen HN (2000) NSP4 gene analysis of rotaviruses recovered from infected children with and without diarrhea. J Clin Microbiol 38:4471–4477
