# Supplementary material for: Development of a live attenuated trivalent porcine rotavirus A vaccine against disease caused by recent strains most prevalent in South Korea
Source: Vet Res. 2019 Jan 7;50:2. doi: 10.1186/s13567-018-0619-6 (PMC6323864; doi:10.1186/s13567-018-0619-6)
Supplement: Supplementary file 3 — Additional file 3. Full-length ORF nucleotide sequence identities (%) of ORFa in 11 genomic segments between three Korean porcine 174-1, PRG942, and K71 strains and other known strains representative of corresponding or neighbor genotypes. The nucleotide sequences of open reading frame of the virulent and attenuated porcine 174-1, PRG942, and K71 rotavirus strains were compared with known RVA strains. The values represent the nucleotide similarity of porcine 174-1, PRG942, and K71with the reference strains. [file 13567_2018_619_MOESM3_ESM.doc]

**Additional file 3 Full-length ORF nucleotide sequence identities (%) of ORF^a^ in 11 genomic segments between three Korean porcine 174-1, PRG942, and K71 strains and other known strains representative of corresponding or neighbor genotypes.**

| Segments | Genotype | Strain^b^ | Accession Number | nt % identity with strains^c^ | | |
| --- | --- | --- | --- | --- | --- | --- |
|  |  |  |  | 174-1 | PRG942 | K71 |
| VP7 | G1 | RVA/Human-tc/USA/Wa/1974/G1P1A[8] | M21843 | 71.7 | 74.7 | 75.5 |
|  | G1 | RVA/Human-wt/BEL/BE00038/2008/G1P[8] | HQ392337 | 72.0 | 75.1 | 75.0 |
|  | G1 | RVA/Panda-tc/CHN/CH-1/2008/G1P[7] | GU188284 | 73.1 | 75.3 | 75.8 |
|  | G2 | RVA/Human-tc/USA/DS-1/1976/G2P1B[4] | EF672581 | 71.4 | 73.5 | 71.8 |
|  | G3 | RVA/Pig-tc/VEN/A131/1988/G3P9[7] | L35055 | 76.1 | 76.8 | 77.8 |
|  | G3 | RVA/Human-wt/USA/DC1497-Bethesda/1976/G3P[8] | FJ947348 | 75.9 | 77.6 | 78.2 |
|  | G3 | RVA/Human-wt/USA/DC2106-Bethesda/1976/G3P[8] | FJ947845 | 75.8 | 77.4 | 78.2 |
|  | G4 | RVA/Pig-tc/USA/Gottfried/1983/G4P[6] | X06759 | 71.0 | 74.4 | 73.6 |
|  | G4 | RVA/Human-wt/USA/DC1208-Bethesda/1980/G4P[8] | HM773862 | 71.2 | 74.5 | 75.5 |
|  | G4 | RVA/Human-wt/USA/DC4608-Bethesda/1980/G4P[8] | HM773906 | 71.2 | 74.5 | 75.5 |
|  | ***G5*** | ***RVA/Pig-tc/KOR/K71/2006/G5P[7]*** | ***MF940443*** | 76.6 | 75.6 | 100.0 |
|  | ***G5*** | ***RVA/Pig-tc/KOR/K71V-20/2006/G5P[7]*** | ***MF940444*** | 76.8 | 76.0 | **99.7** |
|  | ***G5*** | ***RVA/Pig-tc/KOR/K71V-40/2006/G5P[7]*** | ***MF940445*** | 76.8 | 76.0 | **99.7** |
|  | ***G5*** | ***RVA/Pig-tc/KOR/K71V-60/2006/G5P[7]*** | ***MF940446*** | 76.8 | 76.0 | **99.5** |
|  | ***G5*** | ***RVA/Pig-tc/KOR/K71-80/2006/G5P[7]*** | ***MF940447*** | 76.8 | 76.0 | **99.5** |
|  | **G5** | **RVA/Pig-tc/USA/OSU/1977/G5P9[7]** | **X04613** | 76.9 | 75.9 | **99.6** |
|  | **G5** | **RVA/Horse-tc/GBR/H-1/1975/G5P[7]** | **AF242393** | 76.9 | 75.8 | **96.7** |
|  | **G5** | **RVA/Pig-xx/CHN/JL94/XXXX/G5P[7]** | **AY538665** | 76.9 | 75.9 | **99.6** |
|  | **G5** | **RVA/Cow-tc/KOR/KV0407/2004/G5P[7]** | **EU873013** | 76.8 | 76.0 | **99.7** |
|  | **G5** | **RVA/Cow-tc/KOR/KJ44/2004/G5P[1]** | **DQ494393** | 75.9 | 75.1 | **98.6** |
|  | **G5** | **RVA/Cow-tc/KOR/KJ75/2004/G5P[5]** | **DQ494394** | 76.3 | 75.6 | **99.0** |
|  | G6 | RVA/Cow-tc/KOR/KJ9-1/2006/G6P[7] | FJ206034 | 75.2 | 73.8 | 74.0 |
|  | G6 | RVA/Human-tc/USA/Se584/1998/G6P[9] | AF532202 | 75.2 | 75.1 | 76.4 |
|  | G6 | RVA/Cow-tc/VEN/BRV033/1990/G6P6[1] | U62154 | 73.3 | 73.3 | 73.0 |
|  | G6 | RVA/Cow-tc/FRA/RF/1982/G6P[1] | X65940 | 74.4 | 73.5 | 75.2 |
|  | G6 | RVA/Cow-tc/GBR/UK/1973/G6P7[5] | JF693056 | 74.6 | 73.8 | 75.3 |
|  | G6 | RVA/Cow-tc/KOR/KJ19-2/2004/G8P[7] | FJ206095 | 74.7 | 73.4 | 73.4 |
|  | G7 | RVA/Turkey-tc/IRL/Ty-3/1979/G7P[17] | AB080737 | 62.8 | 61.8 | 64.2 |
|  | ***G8*** | ***RVA/Pig-tc/KOR/174-1/2006/G8P[7]*** | ***MF940553*** | ***100.0*** | 75.2 | 76.6 |
|  | ***G8*** | ***RVA/Pig-tc/KOR/174-1V-20/2006/G8P[7]*** | ***MF940554*** | ***100.0*** | *75.2* | *76.6* |
|  | ***G8*** | ***RVA/Pig-tc/KOR/174-1V-40/2006/G8P[7]*** | ***MF940555*** | ***99.9*** | *75.2* | *76.5* |
|  | ***G8*** | ***RVA/Pig-tc/KOR/174-1V-60/2006/G8P[7]*** | ***MF940556*** | ***99.9*** | *75.2* | *76.5* |
|  | ***G8*** | ***RVA/Pig-tc/KOR/174-1V-80/2006/G8P[7]*** | ***MF940557*** | ***100.0*** | *75.2* | *76.6* |
|  | ***G8*** | ***RVA/Pig-tc/KOR/156-1/2006/G8P[7]*** | ***KF500201*** | **99.8** | 75.2 | 76.4 |
|  | ***G8*** | ***RVA/Pig-tc/KOR/C-1/2006/G8P[7]*** | ***KF500223*** | **99.9** | 75.2 | 76.5 |
|  | **G8** | **RVA/Pig-tc/KOR/06-46-2/2006/G8P[7]** | **FJ807854** | **99.9** | 75.2 | 76.5 |
|  | **G8** | **RVA/Pig-tc/KOR/208-1/2006/G8P[7]** | **FJ807866** | **99.8** | 75.1 | 76.4 |
|  | **G8** | **RVA/Pig-tc/KOR/06-261-4/2006/G8P[8]** | **FJ807856** | **99.9** | 75.2 | 76.5 |
|  | **G8** | **RVA/Pig-tc/KOR/07-109-8/2006/G8P[8]** | **FJ807858** | **99.9** | 75.2 | 76.5 |
|  | **G8** | **RVA/Pig-tc/KOR/141-1/2006/G8P[7]** | **FJ807862** | **99.4** | 74.9 | 76.5 |
|  | **G8** | **RVA/Cow-tc/KOR/KJ56-1/2004/G8P[7]** | **KF500179** | **99.8** | 75.1 | 76.4 |
|  | **G8** | **RVA/Cow-tc/KOR/KJ25-1/2006/G8P[7]** | **FJ206044** | **99.5** | 75.1 | 76.5 |
|  | **G8** | **RVA/Cow-tc/JPN/Niigata9801/1998/G8P[X]** | **AB044294** | **98.3** | 75.1 | 76.3 |
|  | **G8** | **RVA/Cow-tc/JPN/BRV16/XXXX/G8P[1]** | **AB077058** | **97.4** | 74.9 | 75.9 |
|  | **G8** | **RVA/Cow-tc/JPN/Sun9/XXXX/G8P[X]** | **AB158431** | **96.4** | 75.3 | 76.4 |
|  | **G8** | **RVA/Cow-tc/JPN/KAG74/XXXX/G8P[X]** | **AB077053** | **95.5** | 73.4 | 74.0 |
|  | **G8** | **RVA/Cow-tc/USA/C-8008/XXXX/G8P[X]** | **U14998** | **94.3** | 74.5 | 75.1 |
|  | **G8** | **RVA/Cow-tc/GBR/678/XXXX/G8P[5]** | **L20883** | **88.1** | 75.8 | 74.4 |
|  | **G8** | **RVA/Cow-tc/IND/UP/2010/G8P[X]** | **JX442786** | **85.5** | 75.2 | 75.4 |
|  | **G8** | **RVA/Human-tc/KEN/B12/1987/G8P[1]** | **HM627547** | **96.9** | 73.9 | 75.1 |
|  | **G8** | **RVA/Human-wt/GHA/GH019-08/2008/G8P[6]** | **KJ748481** | **85.1** | 75.5 | 75.2 |
|  | **G8** | **RVA/Human-xx/NGA/HMG035/XXXX/G8P[1]** | **AF359359** | **84.4** | 74.9 | 75.4 |
|  | **G8** | **RVA/Human-wt/COD/DRC88/2003/G8P[8]** | **DQ005109** | **83.9** | 75.2 | 75.4 |
|  | **G8** | **RVA/Human-tc/JPN/AU109/1994/G8P[4]** | **AB272753** | **84.2** | 75.1 | 75.0 |
|  | **G8** | **RVA/Human-wt/TZA/MRC-DPRU4570/2011/G8P[4]** | **KP752576** | **83.9** | 74.9 | 75.4 |
|  | **G8** | **RVA/Human-wt/HRV/CR2006/2006/G8P[8]** | **JQ988904** | **83.7** | 75.0 | 74.8 |
|  | **G8** | **RVA/Human-wt/ZAF/MRC-DPRU2131/2005/G8P[8]** | **KJ753406** | **84.1** | 74.9 | 74.8 |
|  | **G8** | **RVA/Human-wt/TZA/MRC-DPRU4568/2011/G8P[4]** | **KJ751618** | **83.9** | 74.9 | 75.4 |
|  | **G8** | **RVA/Human-wt/EGYEGY2295/1999/G8P[14]** | **AF104104** | **83.6** | 75.9 | 75.4 |
|  | **G8** | **RVA/Human-wt/UGA/MRC-DPRU1922/2008/G8P[4]** | **KJ752667** | **83.4** | 75.0 | 75.1 |
|  | **G8** | **RVA/Rhesus-tc/USA/PTRV/1990/G8P[1]** | **FJ422138** | **97.6** | 75.4 | 76.4 |
|  | **G8** | **RVA/Sheep-tc/ESP/OVR762/2002/G8P[14]** | **EF554153** | **95.5** | 75.4 | 75.3 |
|  | ***G9*** | ***RVA/Pig-tc/KOR/PRG942/2006/G9P[23]*** | ***MF940498*** | 75.2 | **100.0** | 75.6 |
|  | ***G9*** | ***RVA/Pig-tc/KOR/PRG942V-20/2006/G9P[23]*** | ***MF940499*** | 74.8 | **99.2** | 75.5 |
|  | ***G9*** | ***RVA/Pig-tc/KOR/PRG942V-40/2006/G9P[23]*** | ***MF940500*** | 75.0 | **99.5** | 75.8 |
|  | ***G9*** | ***RVA/Pig-tc/KOR/PRG942V-60/2006/G9P[23]*** | ***MF940501*** | 75.2 | **99.6** | 75.9 |
|  | ***G9*** | ***RVA/Pig-tc/KOR/PRG942V-80/2006/G9P[23]*** | ***MF940502*** | 75.1 | **99.5** | 75.8 |
|  | **G9** | **RVA/Human-tc/USA/WI61/1983/G9P1A[8]** | **EF672623** | 75.9 | **89.5** | 78.6 |
|  | **G9** | **RVA/Human-wt/IND/mcs-13-07/2007/G9P[6]** | **EU753963** | 77.0 | **92.2** | 76.1 |
|  | **G9** | **RVA/Human-wt/BEL/B3458/2003/G9P[8]** | **EF990708** | 77.5 | **92.8** | 76.3 |
|  | **G9** | **RVA/Human-xx/IND/mcs-10/2007/G9P[6]** | **EU753971** | 77.0 | **91.6** | 76.1 |
|  | **G9** | **RVA/Human-xx/IND/mani-97/2006/G9P[19]** | **GQ229050** | 76.1 | **92.3** | 75.8 |
|  | **G9** | **RVA/Human-wt/BEL/BE2001/2008/G9P[6]** | **JQ993318** | 75.6 | **92.1** | 76.6 |
|  | **G9** | **RVA/Pig-tc/KOR/PRG9121/2006/G9P[7]** | **JF796739** | 74.8 | **96.8** | 76.1 |
|  | **G9** | **RVA/Pig-tc/KOR/PRG921/2006/G9P[23]** | **JF796717** | 75.2 | **99.7** | 76.0 |
|  | **G9** | **RVA/Pig-tc/KOR/PRG9235/2006/G9P[23]** | **JF796706** | 75.2 | **99.7** | 76.0 |
|  | G10 | RVA/Human-wt/IND/N155/2003/G10P[11] | EU200798 | 75.0 | 73.9 | 74.8 |
|  | G11 | RVA/Human-wt/ECU/EC2184/200x/G11P[6] | GQ149096 | 75.8 | 75.2 | 82.9 |
|  | G11 | RVA/Pig-tc/VEN/A253/1988/G11P[7] | L24163 | 77.0 | 76.0 | 84.2 |
|  | G12 | RVA/Pig-wt/IND/RU172/2002/G12P[7] | DQ204743 | 73.2 | 76.7 | 74.0 |
|  | G13 | RVA/Horse-tc/GBR/L338/1991/G13P[18] | D13549 | 73.4 | 75.1 | 74.7 |
|  | G14 | RVA/Horse-wt/ARG/E403/2006/G14P[12] | JF712582 | 75.0 | 79.1 | 76.4 |
|  | G15 | RVA/Cow-xx/ARG/B383/1998/G15P[11] | FJ347116 | 70.2 | 73.4 | 74.3 |
|  | G16 | RVA/Mouse-tc/XXX/EHP/1981/G16P[20] | U08425 | 73.0 | 74.4 | 72.4 |
|  | G17 | RVA/Turkey-tc/IRL/Ty-1/1979/G17P[17] | S58166 | 64.8 | 64.7 | 64.7 |
|  | G18 | RVA/Pigeion-tc/JPN/PO-13/1983/G18P[17] | D82979 | 64.5 | 64.8 | 64.6 |
|  | G19 | RVA/Chicken-tc/DEU/02V0002G3/2002/G19P[30] | FJ169861 | 63.9 | 62.8 | 63.8 |
|  | G20 | RVA/Human-wt/ECU/Ecu534/2006/G20P[28] | EU805775 | 73.2 | 73.7 | 75.2 |
|  | G21 | RVA/Cow-wt/JPN/Azuk-1/2006/G21P[29] | AB454421 | 70.9 | 72.3 | 72.9 |
|  | G22 | RVA/Turkey-tc/DEU/03V0002E10/2003/G22P[35] | EU486973 | 63.4 | 63.2 | 64.0 |
|  | G23 | RVA/Pheasant-wt/HUN/Phea14246/2008/G23P[X] | FN393054 | 62.0 | 64.5 | 64.2 |
|  | G24 | RVA/Cow-tc/JPN/Dai-10/2007/G24P[33] | AB513837 | 69.9 | 69.5 | 72.3 |
|  | G25 | RVA/Bat-wt/KEN/KE4852/07/2007/G25P[6] | GU983676 | 73.1 | 73.6 | 73.3 |
|  | G26 | RVA/Pig-wt/JPN/TJ4-1/2010/G26P[X] | AB605258 | 74.4 | 76.2 | 76.7 |
|  | G27 | RVA/SugarGlider-wt/JPN/SG33/2010/G27P[X] | AB621363 | 76.3 | 77.5 | 75.4 |
|  | G28 | RVA/Common_Gull-wt/JPN/Ho374/2013/G28P[39] | LC088223 | 60.2 | 58.8 | 59.3 |
|  | G29 | RVA/Human-wt/BEL/BEF06018/2014/G29P[41] | -^d^ | 74.5 | 74.2 | 73.2 |
| VP4 | P[1] | RVA/Human-tc/KEN/B12/1987/G8P[1] | HM627545 | 75.4 | 72.3 | 75.3 |
|  | P[1] | RVA/Cow-tc/FRA/RF/1982/G6P[1] | U65924 | 74.7 | 73.3 | 74.4 |
|  | P[2] | RVA/Human-tc/KEN/B10/1987/G3P[2] | HM627556 | 76.0 | 74.0 | 75.8 |
|  | P[3] | RVA/Dog-tc/USA/CU-1/1982/G3P[3] | EU708915 | 75.6 | 73.3 | 75.4 |
|  | P[4] | RVA/Human-tc/USA/DS-1/1976/G2P1B[4] | EF672577 | 69.5 | 69.0 | 69.3 |
|  | P[5] | RVA/Cow-tc/GBR/UK/1973/G6P7[5] | JF693051 | 68.3 | 69.4 | 68.1 |
|  | P[6] | RVA/Pig-tc/USA/Gottfried/1983/G4P[6] | M33516 | 69.5 | 70.2 | 69.3 |
|  | ***P[7]*** | ***RVA/Pig-tc/KOR/174-1/2006/G8P[7]*** | ***MF940543*** | **100.0** | 73.3 | **99.7** |
|  | ***P[7]*** | ***RVA/Pig-tc/KOR/174-1V-20/2006/G8P[7]*** | ***MF940544*** | **100.0** | 73.3 | **99.7** |
|  | ***P[7]*** | ***RVA/Pig-tc/KOR/174-1V-40/2006/G8P[7]*** | ***MF940545*** | **100.0** | 73.3 | **99.7** |
|  | ***P[7]*** | ***RVA/Pig-tc/KOR/174-1V-60/2006/G8P[7]*** | ***MF940546*** | **99.9** | 73.3 | **99.7** |
|  | ***P[7]*** | ***RVA/Pig-tc/KOR/174-1V-80/2006/G8P[7]*** | ***MF940547*** | **99.9** | 73.2 | **99.6** |
|  | ***P[7]*** | ***RVA/Pig-tc/KOR/K71/2006/G5P[7]*** | ***MF940433*** | **99.7** | 73.0 | **100.0** |
|  | ***P[7]*** | ***RVA/Pig-tc/KOR/K71V-20/2006/G5P[7]*** | ***MF940434*** | **99.7** | 72.9 | **99.7** |
|  | ***P[7]*** | ***RVA/Pig-tc/KOR/K71V-40/2006/G5P[7]*** | ***MF940435*** | **99.9** | 73.2 | **99.8** |
|  | ***P[7]*** | ***RVA/Pig-tc/KOR/K71V-60/2006/G5P[7]*** | ***MF940436*** | **99.8** | 73.1 | **99.8** |
|  | ***P[7]*** | ***RVA/Pig-tc/KOR/K71-80/2006/G5P[7]*** | ***MF940437*** | **99.7** | 73.0 | **99.7** |
|  | **P[7]** | **RVA/Pig-tc/KOR/156-1/2006/G8P[7]** | **KF500199** | **99.4** | 73.0 | **99.2** |
|  | **P[7]** | **RVA/Pig-tc/KOR/C-1/2006/G8P[7]** | **KF500221** | **100.0** | 73.2 | **99.8** |
|  | **P[7]** | **RVA/Pig-tc/USA/OSU/1977/G5P[7]** | **X13190** | **99.4** | 73.1 | **99.4** |
|  | **P[7]** | **RVA/Pig-xx/CHN/JL94/XXXX/G5P[7]** | **AY523636** | **99.4** | 73.0 | **99.3** |
|  | **P[7]** | **RVA/Pig-tc/IND/RU172/XXXX/G12P[7]** | **DQ204742** | **92.1** | 68.0 | **92.1** |
|  | **P[7]** | **RVA/Pig-tc/MEX/YM/1983/G11P[7]** | **M63231** | **92.6** | 73.1 | **92.4** |
|  | **P[7]** | **RVA/Pig-tc/VEN/A131/1988/G3P[7]** | **-^d^** | **93.2** | 71.5 | **93.1** |
|  | **P[7]** | **RVA/Pig-tc/VEN/A253/1988/G11P[7]** | **-^d^** | **93.4** | 71.5 | **93.3** |
|  | **P[7]** | **RVA/Pig-tc/KOR/PRG9121/2006/G9P[7]** | **JF796737** | **92.8** | 73.1 | **92.6** |
|  | **P[7]** | **RVA/Pig-tc/BEL/RV277/1977/G1P[7]** | **KM820722** | **95.4** | 73.2 | **95.1** |
|  | **P[7]** | **RVA/Pig-tc/AUS/CRW-8/1987/G3P[7]** | **L07888** | **94.2** | 73.1 | **94.0** |
|  | **P[7]** | **RVA/Pig-wt/JPN/BU2/2014/G5P[7]** | **AB924087** | **91.9** | 73.2 | **91.6** |
|  | **P[7]** | **RVA/Panda-tc/CHN/CH-1/2008/G1P[7]** | **HQ641296** | **99.4** | 73.0 | **99.3** |
|  | **P[7]** | **RVA/Horse-tc/GBR/H-1/1975/G5P[7]** | **FJ870377** | **94.3** | 72.1 | **94.1** |
|  | **P[7]** | **RVA/Cow-tc/KOR/KV0407/2004/G5P[7]** | **EU873009** | **99.6** | 73.0 | **99.5** |
|  | **P[7]** | **RVA/Cow-tc/KOR/KJ19-2/2006/G6P[7]** | **HM988969** | **99.4** | 73.1 | **99.3** |
|  | **P[7]** | **RVA/Cow-tc/KOR/KJ25-1/2006/G8P[7]** | **HM988968** | **99.5** | 73.0 | **99.4** |
|  | **P[7]** | **RVA/Cow-tc/KOR/K5/2004/G5P[7]** | **JX971572** | **99.9** | 73.1 | 99.8 |
|  | P[8] | RVA/Human-tc/USA/Wa/1974/G1P1A[8] | L34161 | 68.6 | 68.8 | 68.4 |
|  | P[8] | RVA/Human-wt/BEL/B3458/2003/G9P[8] | EF990707 | 68.7 | 69.2 | 68.5 |
|  | P[8] | RVA/Human-wt/COD/DRC88/2003/G8P[8] | DQ005111 | 68.6 | 69.1 | 68.4 |
|  | P[8] | RVA/Human-wt/BEL/BE00038/2008/G1P[8] | HQ392340 | 68.6 | 69.1 | 68.4 |
|  | P[8] | RVA/Human-tc/USA/WI61/1983/G9P1A[8] | EF672619 | 68.6 | 69.5 | 68.3 |
|  | P[8] | RVA/Human-wt/USA/DC1208-Bethesda/1980/G4P[8] | HM773857 | 68.6 | 69.4 | 68.3 |
|  | P[8] | RVA/Human-wt/USA/DC4608-Bethesda/1980/G4P[8] | HM773901 | 68.6 | 69.3 | 68.3 |
|  | P[8] | RVA/Human-wt/USA/DC1497-Bethesda/1976/G3P[8] | FJ947343 | 68.5 | 69.2 | 68.3 |
|  | P[8] | RVA/Human-wt/USA/DC2106-Bethesda/1976/G3P[8] | FJ947840 | 68.6 | 69.3 | 68.4 |
|  | P[9] | RVA/Human-tc/USA/Se584/1998/G6P[9] | EF672605 | 67.4 | 66.2 | 67.4 |
|  | P[10] | RVA/Human-tc/IND/69M/1980/G8P4[10] | M60600 | 73.5 | 72.8 | 73.2 |
|  | P[11] | RVA/Human-wt/IND/N155/2003/G10P[11] | EU200796 | 60.1 | 60.2 | 59.9 |
|  | P[12] | RVA/Horse-wt/ARG/E403/2006/G14P[12] | JF712580 | 73.8 | 73.8 | 73.7 |
|  | P[13] | RVA/Human-wt/IND/HP140/1987/G6P[13] | DQ003291 | 73.7 | 71.7 | 73.5 |
|  | P[14] | RVA/Human-wt/HUN/Hun5/1997/G6P[14] | EF554107 | 68.5 | 66.0 | 68.4 |
|  | P[15] | RVA/Sheep-tc/CHN/Lamb-NT/XXXX/G10P[15] | FJ031027 | 73.0 | 73.9 | 72.9 |
|  | P[16] | RVA/Mouse-tc/USA/ETD_822/XXXX/G16P[16] | GQ479950 | 68.6 | 69.2 | 68.5 |
|  | P[17] | RVA/Pigeion-tc/JPN/PO-13/1983/G18P[17] | AB009632 | 63.2 | 61.4 | 63.0 |
|  | P[18] | RVA/Horse-tc/GBR/L338/1991/G13P[18] | JF712558 | 75.1 | 71.9 | 74.9 |
|  | P[19] | RVA/Human-wt/IND/RMC321/1990/G9P[19] | AF523677 | 71.9 | 71.7 | 71.7 |
|  | P[20] | RVA/Mouse-tc/XXX/EHP/1981/G16P[20] | U08424 | 69.8 | 69.2 | 69.5 |
|  | P[21] | RVA/Cow-tc/IND/Hg18/XXXX/G15P[21] | AF237665 | 71.2 | 70.6 | 71.1 |
|  | ***P[23]*** | ***RVA/Pig-tc/KOR/PRG942/2006/G9P[23]*** | ***MF940488*** | 73.3 | **100.0** | 73.0 |
|  | ***P[23]*** | ***RVA/Pig-tc/KOR/PRG942V-20/2006/G9P[23]*** | ***MF940489*** | 73.3 | **99.3** | 73.1 |
|  | ***P[23]*** | ***RVA/Pig-tc/KOR/PRG942V-40/2006/G9P[23]*** | ***MF940490*** | 72.9 | **99.4** | 72.7 |
|  | ***P[23]*** | ***RVA/Pig-tc/KOR/PRG942V-60/2006/G9P[23]*** | ***MF940491*** | 72.8 | **99.3** | 72.6 |
|  | ***P[23]*** | ***RVA/Pig-tc/KOR/PRG942V-80/2006/G9P[23]*** | ***MF940492*** | 73.1 | **99.7** | 72.9 |
|  | **P[23]** | **RVA/Pig-tc/KOR/PRG921/2006/G9P[23]** | **JF796715** | 73.9 | **98.7** | 73.7 |
|  | **P[23]** | **RVA/Pig-tc/KOR/PRG9235/2006/G9P[23]** | **JF796704** | 73.9 | **98.7** | 73.7 |
|  | **P[23]** | **RVA/Pig-wt/JPN/GUB71/2006/G4P[23]** | **AB573648** | 73.7 | **89.1** | 73.5 |
|  | **P[23]** | **RVA/Pig-wt/JPN/GUB46/2006/G9P[23]/** | **AB573646** | 73.8 | **88.2** | 73.6 |
|  | **P[23]** | **RVA/Pig-xx/THA/CMP48/08/2008/G3P[23]/** | **HQ268847** | 73.5 | **83.3** | 73.3 |
|  | P[24] | RVA/Rhesus-tc/USA/TUCH/2002/G3P[24] | AY596189 | 74.0 | 75.3 | 73.7 |
|  | P[25] | RVA/Human-wt/NPL/KTM368/2004/G11P[25] | GU199495 | 66.6 | 66.5 | 66.6 |
|  | P[26] | RVA/Pig-wt/ITA/134/04-15/2003/G5P[26] | DQ061053 | 74.8 | 72.1 | 74.6 |
|  | P[27] | RVA/Pig-wt/SVN/P21-5/2004/G1P[27] | DQ629926 | 69.9 | 70.7 | 69.8 |
|  | P[28] | RVA/Human-wt/ECU/Ecu534/2006/G20P[28] | EU805773 | 71.2 | 69.8 | 71.0 |
|  | P[29] | RVA/Cow-wt/JPN/Azuk-1/2006/G21P[29] | AB454420 | 65.5 | 64.2 | 65.2 |
|  | P[30] | RVA/Chicken-tc/DEU/02V0002G3/2002/G19P[30] | FJ169856 | 63.6 | 61.9 | 63.5 |
|  | P[31] | RVA/Chicken-tc/DEU/06V0661/2006/G19P[31] | EU486962 | 63.0 | 63.0 | 63.0 |
|  | P[32] | RVA/Pig-wt/IRL/61/07-ire/2007/G2P[32] | FJ492835 | 75.0 | 70.2 | 74.8 |
|  | P[33] | RVA/Cow-tc/JPN/Dai-10/2007/G24P[33] | AB513836 | 71.2 | 71.1 | 71.0 |
|  | P[34] | RVA/Pig-wt/JPN/FGP51/2009/G4P[34] | AB571047 | 69.2 | 70.9 | 69.1 |
|  | P[35] | RVA/Turkey-tc/DEU/03V0002E10/2003/G22P[35] | EU486958 | 63.5 | 62.2 | 63.3 |
|  | P[36] | RVA/SugarGlider-wt/JPN/SG385/2012/G27P[36] | AB823215 | 72.4 | 72.8 | 72.2 |
|  | P[37] | RVA/Pheasant-tc/GER/10V0112H5/2010/G23P[37] | JX204814 | 65.4 | 65.8 | 65.3 |
|  | P[38] | RVA/Turkey-tc/IRL/Ty-1/1979/G17P[38] | LC088110 | 62.9 | 63.0 | 62.7 |
|  | P[39] | RVA/Common_Gull-wt/JPN/Ho374/2013/G28P[39] | LC088221 | 56.5 | 56.9 | 56.4 |
|  | P[40] | RVA/Alpaca-tc/PER/SA44/2014/G3P[40] | KT935478 | 72.3 | 74.6 | 72.1 |
|  | P[41] | RVA/Human-wt/BEL/BEF06018/2014/G29P[41] | -^d^ | 73.8 | 70.8 | 73.6 |
| VP6 | I1 | RVA/Human-tc/USA/Wa/1974/G1P1A[8] | K02086 | 82.1 | 83.4 | 82.0 |
|  | I1 | RVA/Pig-tc/USA/Gottfried/1983/G4P[6] | D00326 | 82.4 | 83.2 | 82.3 |
|  | I1 | RVA/Human-wt/BEL/B3458/2003/G9P[8] | DQ870504 | 82.5 | 83.6 | 82.4 |
|  | I1 | RVA/Human-xx/IND/mcs-10/2007/G9P[6] | EU753972 | 82.2 | 83.3 | 82.2 |
|  | I1 | RVA/Human-wt/BEL/BE00038/2008/G1P[8] | HQ392338 | 82.7 | 83.8 | 82.7 |
|  | I1 | RVA/Human-tc/USA/WI61/1983/G9P1A[8] | EF583052 | 82.6 | 83.8 | 82.5 |
|  | I1 | RVA/Human-xx/IND/mani-253/2007/G3P[8] | HM348745 | 83.6 | 83.8 | 83.4 |
|  | I1 | RVA/Human-xx/IND/mani-362/2007/G4P[6] | HM348747 | 83.6 | 83.4 | 83.4 |
|  | I1 | RVA/Human-wt/IND/mcs-13-07/2007/G9P[6] | EU753964 | 82.0 | 82.7 | 81.9 |
|  | I1 | RVA/Human-wt/ECU/EC2184/200x/G11P[6] | GQ149095 | 83.7 | 84.6 | 83.6 |
|  | I1 | RVA/Human-wt/USA/DC1208-Bethesda/1980/G4P[8] | HM773859 | 83.9 | 84.0 | 83.8 |
|  | I1 | RVA/Human-wt/USA/DC4608-Bethesda/1980/G4P[8] | HM773903 | 83.9 | 84.0 | 83.8 |
|  | I1 | RVA/Human-wt/USA/DC1497-Bethesda/1976/G3P[8] | FJ947345 | 83.3 | 84.3 | 83.2 |
|  | I1 | RVA/Human-wt/USA/DC2106-Bethesda/1976/G3P[8] | FJ947842 | 83.0 | 84.2 | 82.9 |
|  | I2 | RVA/Human-tc/USA/DS-1/1976/G2P1B[4] | HQ650121 | 79.1 | 80.6 | 79.1 |
|  | I3 | RVA/Dog-tc/USA/CU-1/1982/G3P[3] | EU708916 | 79.1 | 78.6 | 78.9 |
|  | I4 | RVA/Turkey-tc/IRL/Ty-1/1979/G17P[17] | D82980 | 69.4 | 70.2 | 69.3 |
|  | ***I5*** | ***RVA/Pig-tc/KOR/174-1/2006/G8P[7]*** | ***MF940548*** | **100.0** | **90.0** | **99.7** |
|  | ***I5*** | ***RVA/Pig-tc/KOR/174-1V-20/2006/G8P[7]*** | ***MF940549*** | **100.0** | **90.0** | **99.7** |
|  | ***I5*** | ***RVA/Pig-tc/KOR/174-1V-40/2006/G8P[7]*** | ***MF940550*** | **100.0** | **90.0** | **99.7** |
|  | ***I5*** | ***RVA/Pig-tc/KOR/174-1V-60/2006/G8P[7]*** | ***MF940551*** | **100.0** | **90.0** | **99.7** |
|  | ***I5*** | ***RVA/Pig-tc/KOR/174-1V-80/2006/G8P[7]*** | ***MF940552*** | **99.7** | **89.8** | **99.4** |
|  | ***I5*** | ***RVA/Pig-tc/KOR/PRG942/2006/G9P[23]*** | ***MF940493*** | **90.0** | **100.0** | **89.9** |
|  | ***I5*** | ***RVA/Pig-tc/KOR/PRG942V-20/2006/G9P[23]*** | ***MF940494*** | **90.0** | **100.0** | **89.9** |
|  | ***I5*** | ***RVA/Pig-tc/KOR/PRG942V-40/2006/G9P[23]*** | ***MF940495*** | **90.0** | **100.0** | **89.9** |
|  | ***I5*** | ***RVA/Pig-tc/KOR/PRG942V-60/2006/G9P[23]*** | ***MF940496*** | **90.0** | **100.0** | **89.9** |
|  | ***I5*** | ***RVA/Pig-tc/KOR/PRG942V-80/2006/G9P[23]*** | ***MF940497*** | **90.0** | **100.0** | **89.9** |
|  | ***I5*** | ***RVA/Pig-tc/KOR/K71/2006/G5P[7]*** | ***MF940438*** | **99.7** | **89.9** | **100.0** |
|  | ***I5*** | ***RVA/Pig-tc/KOR/K71V-20/2006/G5P[7]*** | ***MF940439*** | **99.8** | **90.0** | **99.7** |
|  | ***I5*** | ***RVA/Pig-tc/KOR/K71V-40/2006/G5P[7]*** | ***MF940440*** | **99.9** | **90.1** | **99.7** |
|  | ***I5*** | ***RVA/Pig-tc/KOR/K71V-60/2006/G5P[7]*** | ***MF940441*** | **99.7** | **89.9** | **99.6** |
|  | ***I5*** | ***RVA/Pig-tc/KOR/K71-80/2006/G5P[7]*** | ***MF940442*** | **99.9** | **90.1** | **99.7** |
|  | **I5** | **RVA/Pig-tc/KOR/156-1/2006/G8P[7]** | **KF500200** | **89.9** | **95.7** | **89.8** |
|  | **I5** | **RVA/Pig-tc/KOR/C-1/2006/G8P[7]** | **KF500222** | **99.7** | **90.0** | **99.5** |
|  | **I5** | **RVA/Pig-tc/KOR/PRG9121/2006/G9P[7]** | **JF796738** | **94.6** | **90.4** | **94.4** |
|  | **I5** | **RVA/Pig-tc/KOR/PRG9235/2006/G9P[23]** | **JF796705** | **90.1** | **99.9** | **89.9** |
|  | **I5** | **RVA/Pig-tc/KOR/PRG921/2006/G9P[23]** | **JF796716** | **90.0** | **99.8** | **89.9** |
|  | **I5** | **RVA/Cow-tc/KOR/K5/2004/G5P[7]** | **JX971573** | **99.9** | **90.1** | **99.7** |
|  | **I5** | **RVA/Pig-tc/VEN/A131/1988/G3P9[7]** | **AF317124** | **87.3** | **87.5** | **87.2** |
|  | **I5** | **RVA/Pig-tc/USA/OSU/1977/G5P9[7]** | **AF317123** | **86.6** | **87.5** | **86.5** |
|  | **I5** | **RVA/Horse-tc/GBR/H-1/1975/G5P[7]** | **AF242394** | **87.4** | **88.2** | **87.4** |
|  | **I5** | **RVA/Pig-xx/CHN/JL94/XXXX/G5P[7]** | **AY538664** | **99.6** | **90.1** | **99.4** |
|  | **I5** | **RVA/Cow-tc/KOR/KV0407/2004/G5P[7]** | **EU873010** | **99.9** | **90.1** | **99.7** |
|  | **I5** | **RVA/Panda-tc/CHN/CH-1/2008/G1P[7]** | **GU188283** | **99.1** | **89.9** | **98.9** |
|  | **I5** | **RVA/Cow-tc/KOR/KJ25-1/2006/G8P[7]** | **HM988972** | **90.0** | **86.9** | **89.9** |
|  | **I5** | **RVA/Pig-tc/VEN/A253/1988/G11P9[7]** | **AF317122** | **86.5** | **87.0** | **86.3** |
|  | **I5** | **RVA/Pig-tc/MEX/YM/1983/G11P9[7]** | **X69487** | **89.0** | **90.0** | **88.9** |
|  | **I5** | **RVA/Pig-xx/CHN/GD/2008/GXP[X]** | **FJ617209** | **95.6** | **89.9** | **95.4** |
|  | **I5** | **RVA/Human-xx/IND/mani-97/2006/G9P[19]** | **HM348744** | **89.3** | **89.4** | **89.1** |
|  | **I5** | **RVA/Pig-wt/IND/RU172/2002/G12P[7]** | **DQ204741** | **89.7** | **89.9** | **89.5** |
|  | **I5** | **RVA/Human-wt/BEL/BE2001/2008/G9P[6]** | **JQ993320** | **90.6** | **93.6** | **90.5** |
|  | **I5** | **RVA/Human-wt/THA/BMc323/1989/G9P[19]** | **JN104614** | **90.0** | **94.0** | **89.9** |
|  | **I5** | **RVA/Human-wt/HUN/BP1547/2005/G4P[6]** | **KF835926** | **90.8** | **95.1** | **90.6** |
|  | **I5** | **RVA/Human-wt/JPN/Ryukyu-1120/2011/G5P[6]** | **AB741653** | **89.9** | **94.1** | **89.7** |
|  | **I5** | **RVA/Human-wt/CHN/LL3354/2000/G5P[6]** | **KC139784** | **89.1** | **91.4** | **88.9** |
|  | I6 | RVA/Horse-tc/GBR/L338/1991/G13P[18] | JF712559 | 79.6 | 80.2 | 79.4 |
|  | I7 | RVA/Mouse-tc/USA/ETD_822/XXXX/G16P[16] | GQ479952 | 75.7 | 75.7 | 75.5 |
|  | I8 | RVA/Human-wt/THA/CMH222/2001/G3P[3] | DQ288659 | 78.8 | 79.9 | 78.6 |
|  | I9 | RVA/Rhesus-tc/USA/TUCH/2002/G3P[24] | AY594670 | 79.8 | 79.8 | 79.6 |
|  | I10 | RVA/Sheep-tc/CHN/Lamb-NT/XXXX/G10P[15] | FJ031028 | 78.9 | 79.2 | 78.7 |
|  | I11 | RVA/Chicken-tc/DEU/02V0002G3/2002/G19P[30] | FJ169858 | 70.1 | 70.2 | 70.2 |
|  | I12 | RVA/Human-wt/NPL/KTM368/2004/G11P[25] | GU199496 | 84.5 | 84.9 | 84.3 |
|  | I13 | RVA/Human-wt/ECU/Ecu534/2006/G20P[28] | EU805774 | 76.2 | 76.4 | 76.1 |
|  | I14 | RVA/Pig-wt/CAN/CE-M-06-0003/2005/G2P[27] | GU183245 | 79.1 | 80.6 | 78.9 |
|  | I15 | RVA/Bat-wt/KEN/KE4852/07/2007/G25P[6] | GU983675 | 77.1 | 77.5 | 77.0 |
|  | I16 | RVA/Human-tc/KEN/B10/1987/G3P[2] | HM627557 | 78.7 | 79.1 | 78.6 |
|  | I17 | RVA/Rabbit-tc/CHN/N5/1992/G3P[14] | JQ423906 | 78.3 | 78.8 | 78.2 |
|  | I18 | RVA/Human-wt/BRA/QUI-35-F5/2010/G3P[9] | KF185107 | 78.4 | 78.3 | 78.3 |
|  | I19 | RVA/SugarGlider-tc/JPN/SG385/2012/G27P[36] | AB971764 | 77.7 | 77.6 | 77.7 |
|  | I20 | RVA/Rat-wt/GER/KS-11-573/2011/G3P[3] | KJ879452 | 77.8 | 77.8 | 77.6 |
|  | I21 | RVA/Common_Gull-wt/JPN/Ho374/2013/G28P[39] | LC088222 | 69.2 | 69.3 | 69.2 |
| VP1 | ***R1*** | ***RVA/Pig-tc/KOR/174-1/2006/G8P[7]*** | ***MF940528*** | **100.0** | **85.8** | **99.6** |
|  | ***R1*** | ***RVA/Pig-tc/KOR/174-1V-20/2006/G8P[7]*** | ***MF940529*** | **100.0** | **85.8** | **99.6** |
|  | ***R1*** | ***RVA/Pig-tc/KOR/174-1V-40/2006/G8P[7]*** | ***MF940530*** | **100.0** | **85.8** | **99.6** |
|  | ***R1*** | ***RVA/Pig-tc/KOR/174-1V-60/2006/G8P[7]*** | ***MF940531*** | **100.0** | **85.8** | **99.6** |
|  | ***R1*** | ***RVA/Pig-tc/KOR/174-1V-80/2006/G8P[7]*** | ***MF940532*** | **99.7** | **85.6** | **99.3** |
|  | ***R1*** | ***RVA/Pig-tc/KOR/PRG942/2006/G9P[23]*** | ***MF940473*** | **85.8** | **100.0** | **85.8** |
|  | ***R1*** | ***RVA/Pig-tc/KOR/PRG942V-20/2006/G9P[23]*** | ***MF940474*** | **85.8** | **100.0** | **85.8** |
|  | ***R1*** | ***RVA/Pig-tc/KOR/PRG942V-40/2006/G9P[23]*** | ***MF940475*** | **85.9** | **99.8** | **85.8** |
|  | ***R1*** | ***RVA/Pig-tc/KOR/PRG942V-60/2006/G9P[23]*** | ***MF940476*** | **85.8** | **99.7** | **85.8** |
|  | ***R1*** | ***RVA/Pig-tc/KOR/PRG942V-80/2006/G9P[23]*** | ***MF940477*** | **85.8** | **99.9** | **85.7** |
|  | ***R1*** | ***RVA/Pig-tc/KOR/K71/2006/G5P[7]*** | ***MF940418*** | **99.6** | **85.8** | **100.0** |
|  | ***R1*** | ***RVA/Pig-tc/KOR/K71V-20/2006/G5P[7]*** | ***MF940419*** | **99.6** | **85.8** | **99.8** |
|  | ***R1*** | ***RVA/Pig-tc/KOR/K71V-40/2006/G5P[7]*** | ***MF940420*** | **99.5** | **85.7** | **99.7** |
|  | ***R1*** | ***RVA/Pig-tc/KOR/K71V-60/2006/G5P[7]*** | ***MF940421*** | **99.5** | **85.8** | **99.7** |
|  | ***R1*** | ***RVA/Pig-tc/KOR/K71-80/2006/G5P[7]*** | ***MF940422*** | **99.6** | **85.8** | **99.8** |
|  | **R1** | **RVA/Pig-tc/KOR/156-1/2006/G8P[7]** | **KF500196** | **94.5** | **85.9** | **94.5** |
|  | **R1** | **RVA/Pig-tc/KOR/C-1/2006/G8P[7]** | **KF500218** | **94.1** | **86.0** | **94.1** |
|  | **R1** | **RVA/Pig-tc/KOR/K71/2006/G5P[7]** | **JX971580** | **99.7** | **85.9** | **99.8** |
|  | **R1** | **RVA/Pig-tc/USA/OSU/1977/G5P9[7]** | **GU199514** | **99.4** | **85.8** | **99.6** |
|  | **R1** | **RVA/Pig-tc/VEN/A131/1988/G3P9[7]** | **EF560618** | **85.5** | **84.4** | **85.5** |
|  | **R1** | **RVA/Pig-tc/VEN/A253/1988/G11P9[7]** | **EF560621** | **85.1** | **84.3** | **85.2** |
|  | **R1** | **RVA/Pig-tc/MEX/YM/1983/G11P9[7]** | **X76486** | **87.3** | **86.0** | **87.4** |
|  | **R1** | **RVA/Pig-tc/USA/Gottfried/1983/G4P[6]** | **M32805** | **96.0** | **85.3** | **96.2** |
|  | **R1** | **RVA/Pig-tc/KOR/PRG921/2006/G9P[23]** | **JF796712** | **85.8** | **99.5** | **85.8** |
|  | **R1** | **RVA/Pig-tc/KOR/PRG9235/2006/G9P[23]** | **JF796701** | **85.9** | **99.6** | **85.9** |
|  | **R1** | **RVA/Pig-tc/KOR/PRG9121/2006/G9P[7]** | **JF796734** | **86.1** | **94.3** | **86.1** |
|  | **R1** | **RVA/Pig-wt/IND/RU172/2002/G12P[7]** | **GU199191** | **86.2** | **87.1** | **86.3** |
|  | **R1** | **RVA/Cow-tc/KOR/K5/2004/G5P[7]** | **JX971569** | **99.7** | **85.9** | **99.8** |
|  | **R1** | **RVA/Cow-tc/KOR/KJ25-1/2006/G8P[7]** | **HM988965** | **95.3** | **85.0** | **95.4** |
|  | **R1** | **RVA/Horse-tc/GBR/H-1/1975/G5P[7]** | **JQ309138** | **86.5** | **88.5** | **86.6** |
|  | **R1** | **RVA/Panda-tc/CHN/CH-1/2008/G1P[7]** | **HQ641297** | **99.5** | **86.0** | **99.7** |
|  | **R1** | **RVA/Human-tc/USA/Wa/1974/G1P1A[8]** | **DQ490539** | **86.4** | **87.5** | **86.4** |
|  | **R1** | **RVA/Human-wt/BEL/BE2001/2008/G9P[6]** | **JQ993321** | **94.2** | **86.2** | **94.3** |
|  | **R1** | **RVA/Human-wt/USA/DC1208-Bethesda/1980/G4P[8]** | **HM773854** | **87.0** | **86.3** | **87.1** |
|  | **R1** | **RVA/Human-wt/USA/DC4608-Bethesda/1980/G4P[8]** | **HM773898** | **87.0** | **86.2** | **87.1** |
|  | **R1** | **RVA/Human-wt/BEL/B3458/2003/G9P[8]** | **DQ870501** | **86.4** | **87.4** | **86.5** |
|  | **R1** | **RVA/Human-wt/BEL/BE00038/2008/G1P[8]** | **HQ392339** | **86.4** | **87.4** | **86.6** |
|  | **R1** | **RVA/Human-tc/USA/WI61/1983/G9P1A[8]** | **EF583049** | **86.3** | **87.4** | **86.3** |
|  | **R1** | **RVA/Human-wt/USA/DC1497-Bethesda/1976/G3P[8]** | **FJ947340** | **86.3** | **87.4** | **86.3** |
|  | **R1** | **RVA/Human-wt/USA/DC2106-Bethesda/1976/G3P[8]** | **FJ947837** | **86.3** | **87.3** | **86.3** |
|  | **R1** | **RVA/Human-wt/USA/VU08-09-16/2008/G3P[8]** | **JF790315** | **86.4** | **87.4** | **86.5** |
|  | **R1** | **RVA/Human-wt/ITA/AV21/2010/G9P[8]** | **JX195063** | **86.1** | **87.1** | **86.2** |
|  | **R1** | **RVA/Human-tc/IND/61060/2006/G1P8** | **HQ609554** | **86.3** | **87.4** | **86.5** |
|  | **R1** | **RVA/Human-tc/CHN/Y128/2004/G1P[8]** | **JQ087423** | **86.2** | **87.2** | **86.3** |
|  | **R1** | **RVA/Human-wt/UGA/MRC-DPRU1944/2008/G9P[8]** | **KJ751757** | **86.6** | **87.7** | **86.7** |
|  | **R1** | **RVA/human-wt/USA/DC4320/1988/G4P[8]** | **HM773887** | **86.6** | **87.2** | **86.6** |
|  | **R1** | **RVA/Human-wt/BEL/BE00043/2009/G1P[8]** | **HQ392377** | **86.4** | **87.4** | **86.4** |
|  | **R1** | **RVA/Human-wt/DEU/GER26-08/2008/G12P[8]** | **FJ747613** | **86.6** | **87.5** | **86.6** |
|  | **R1** | **RVA/Human-tc/JPN/YO/1977/G3P1A[8]** | **DQ870497** | **85.7** | **89.6** | **85.7** |
|  | **R1** | **RVA/Human-wt/USA/DC1476/1974/G1P[8]** | **KC579564** | **87.7** | **86.0** | **87.7** |
|  | **R1** | **RVA/Human-wt/USA/DC102/1974/G1P[8]** | **KC80005** | **87.5** | **85.8** | **87.6** |
|  | R2 | RVA/Human-tc/USA/DS-1/1976/G2P1B[4] | EF990691 | 78.9 | 78.6 | 78.9 |
|  | R2 | RVA/Human-wt/COD/DRC88/2003/G8P[8] | DQ005114 | 78.2 | 78.5 | 78.3 |
|  | R2 | RVA/Cow-tc/GBR/UK/1973/G6P7[5] | JF693048 | 78.5 | 78.3 | 78.7 |
|  | R2 | RVA/Human-tc/KEN/B12/1987/G8P[1] | HM627542 | 78.6 | 77.9 | 78.7 |
|  | R2 | RVA/Human-tc/USA/Se584/1998/G6P[9] | EF583041 | 78.1 | 78.3 | 78.1 |
|  | R2 | RVA/Cow-tc/FRA/RF/1982/G6P[1] | J04346 | 78.2 | 78.5 | 78.2 |
|  | R2 | RVA/Cow-tc/VEN/BRV033/1990/G6P6[1] | EF560612 | 77.0 | 77.4 | 77.1 |
|  | R2 | RVA/Cow-tc/KOR/KJ19-2/2004/G8P[7] | HM988966 | 78.2 | 78.4 | 78.3 |
|  | R2 | RVA/Cow-tc/KOR/KJ9-1/2006/G6P[7] | HM988967 | 78.5 | 78.8 | 78.6 |
|  | R3 | RVA/Dog-tc/USA/CU-1/1982/G3P[3] | EU708912 | 100.0 | 85.8 | 99.6 |
|  | R4 | RVA/Pigeon-tc/JPN/PO-13/1983/G18P[17] | AB009629 | 99.8 | 85.9 | 99.8 |
|  | R5 | RVA/Cow-xx/ARG/B383/1998/G15P[11] | FJ347111 | 79.6 | 78.5 | 79.6 |
|  | R6 | RVA/Chicken-tc/DEU/02V0002G3/2002/G19P[30] | FJ169853 | 71.6 | 71.1 | 71.4 |
|  | R7 | RVA/Mouse-tc/USA/ETD_822/XXXX/G16P[16] | GQ479947 | 77.7 | 77.4 | 77.8 |
|  | R8 | RVA/Human-tc/KEN/B10/1987/G3P[2] | HM627553 | 70.4 | 71.1 | 70.3 |
|  | R9 | RVA/Horse-tc/GBR/L338/1991/G13P[18] | JF712555 | 73.8 | 72.3 | 73.6 |
|  | R10 | RVA/SugarGlider-tc/JPN/SG385/2012/G27P[36] | AB971760 | 79.8 | 80.2 | 79.8 |
|  | R11 | RVA/Rat-wt/GER/KS-11-573/2011/G3P[3] | KJ879448 | 78.3 | 77.6 | 78.4 |
|  | R12 | RVA/Hu/ITA/ME848-12/2012/G12P[8] | KR632623 | 77.9 | 77.3 | 78.0 |
|  | R13 | RVA/Human-xx/USA/2014735512/xxxx/G20P[28] | -^d^ | 77.6 | 76.4 | 77.6 |
|  | R14 | RVA/Common_Gull-wt/JPN/Ho374/2013/G28P[39] | LC088218 | 79.9 | 79.3 | 79.8 |
| **VP2** | ***C1*** | ***RVA/Pig-tc/KOR/174-1/2006/G8P[7]*** | ***MF940533*** | **100.0** | **92.8** | **96.9** |
|  | ***C1*** | ***RVA/Pig-tc/KOR/174-1V-20/2006/G8P[7]*** | ***MF940534*** | **100.0** | **92.8** | **96.9** |
|  | ***C1*** | ***RVA/Pig-tc/KOR/174-1V-40/2006/G8P[7]*** | ***MF940535*** | **100.0** | **92.7** | **96.9** |
|  | ***C1*** | ***RVA/Pig-tc/KOR/174-1V-60/2006/G8P[7]*** | ***MF940536*** | **99.9** | **92.7** | **96.9** |
|  | ***C1*** | ***RVA/Pig-tc/KOR/174-1V-80/2006/G8P[7]*** | ***MF940537*** | **99.9** | **92.7** | **96.9** |
|  | ***C1*** | ***RVA/Pig-tc/KOR/PRG942/2006/G9P[23]*** | ***MF940478*** | **92.8** | **100.0** | **91.7** |
|  | ***C1*** | ***RVA/Pig-tc/KOR/PRG942V-20/2006/G9P[23]*** | ***MF940479*** | **92.7** | **99.6** | **91.7** |
|  | ***C1*** | ***RVA/Pig-tc/KOR/PRG942V-40/2006/G9P[23]*** | ***MF940480*** | **92.9** | **99.2** | **91.9** |
|  | ***C1*** | ***RVA/Pig-tc/KOR/PRG942V-60/2006/G9P[23]*** | ***MF940481*** | **92.9** | **99.2** | **91.9** |
|  | ***C1*** | ***RVA/Pig-tc/KOR/PRG942V-80/2006/G9P[23]*** | ***MF940482*** | **92.9** | **99.1** | **91.9** |
|  | ***C1*** | ***RVA/Pig-tc/KOR/K71/2006/G5P[7]*** | ***MF940423*** | **96.9** | **91.7** | **100.0** |
|  | ***C1*** | ***RVA/Pig-tc/KOR/K71V-20/2006/G5P[7]*** | ***MF940424*** | **97.0** | **91.8** | **99.8** |
|  | ***C1*** | ***RVA/Pig-tc/KOR/K71V-40/2006/G5P[7]*** | ***MF940425*** | **97.0** | **91.8** | **99.5** |
|  | ***C1*** | ***RVA/Pig-tc/KOR/K71V-60/2006/G5P[7]*** | ***MF940426*** | **97.0** | **91.8** | **99.5** |
|  | ***C1*** | ***RVA/Pig-tc/KOR/K71-80/2006/G5P[7]*** | ***MF940427*** | **97.0** | **91.8** | **99.6** |
|  | **C1** | **RVA/Pig-tc/KOR/156-1/2006/G8P[7]** | **KF500197** | **97.0** | **91.8** | **99.6** |
|  | **C1** | **RVA/Pig-tc/KOR/C-1/2006/G8P[7]** | **KF500219** | **95.1** | **92.3** | **97.6** |
|  | **C1** | **RVA/Pig-tc/KOR/K71/2006/G5P[7]** | **JX971581** | **97.2** | **91.9** | **99.7** |
|  | **C1** | **RVA/Pig-tc/USA/OSU/1977/G5P[7]** | **GU199515** | **97.1** | **92.0** | **99.7** |
|  | **C1** | **RVA/Pig-tc/USA/Gottfried/1983/G4P[6]** | **GU199487** | **92.8** | **91.2** | **93.5** |
|  | **C1** | **RVA/Pig-tc/MEX/YM/1983/G11P[7]** | **GU199516** | **93.4** | **91.4** | **93.7** |
|  | **C1** | **RVA/Pig-tc/KOR/PRG9121/2006/G9P[7]** | **JF796735** | **92.6** | **91.3** | **93.4** |
|  | **C1** | **RVA/Pig-tc/KOR/PRG9235/2006/G9P[23]** | **JF796702** | **92.6** | **91.2** | **93.4** |
|  | **C1** | **RVA/Pig-wt/IND/RU172/2002/G12P[7]** | **GU199192** | **92.0** | **90.0** | **92.5** |
|  | **C1** | **RVA/Pig-tc/KOR/PRG921/2006/G9P[23]** | **JF796713** | **93.3** | **99.4** | **92.3** |
|  | **C1** | **RVA/Cow-tc/KOR/K5/2004/G5P[7]** | **JX971570** | **97.2** | **91.9** | **99.7** |
|  | **C1** | **RVA/Cow-tc/KOR/KJ9-1/2006/G6P[7]** | **HM988960** | **90.2** | **88.2** | **90.3** |
|  | **C1** | **RVA/Cow-tc/KOR/KJ25-1/2006/G8P[7]** | **HM988959** | **90.5** | **88.0** | **90.4** |
|  | **C1** | **RVA/Horse-tc/GBR/H-1/1975/G5P[7]** | **JQ309139** | **95.4** | **91.8** | **96.5** |
|  | **C1** | **RVA/Panda-tc/CHN/CH-1/2008/G1P[7]** | **HQ641294** | **86.6** | **86.6** | **86.5** |
|  | **C1** | **RVA/Human-wt/USA/DC827/1978/G4P[8]** | **HM773954** | **94.1** | **91.7** | **94.5** |
|  | **C1** | **RVA/Human-wt/USA/DC1476/1974/G1P[8]** | **KC579565** | **94.4** | **92.1** | **94.8** |
|  | **C1** | **RVA/Human-wt/USA/DC1208-Bethesda/1980/G4P[8]** | **HM773855** | **93.9** | **91.8** | **94.3** |
|  | **C1** | **RVA/Human-wt/USA/DC2106-Bethesda/1976/G3P[8]** | **FJ947838** | **87.9** | **87.4** | **88.3** |
|  | **C1** | **RVA/Human-wt/HUN/BP1125/2004/G4P[6]** | **KF835898** | **93.0** | **91.4** | **93.4** |
|  | **C1** | **RVA/Human-wt/THA/Mc323/1989/G9P[19]** | **JN104612** | **92.1** | **90.6** | **92.9** |
|  | **C1** | **RVA/Human-wt/BEL/BE2001/2008/G9P[6]** | **JQ993322** | **92.8** | **90.9** | **93.1** |
|  | **C1** | **RVA/Human-wt/USA/DC102/1974/G1P[8]** | **KC580006** | **88.1** | **87.3** | **88.4** |
|  | **C1** | **RVA/Human-tc/USA/Wa/1974/G1P1A[8]** | **X14942** | **88.2** | **87.4** | **88.3** |
|  | **C1** | **RVA/Human-tc/USA/WI61/1983/G9P1A[8]** | **EF583050** | **87.4** | **87.0** | **87.6** |
|  | **C1** | **RVA/Human-wt/BEL/B3458/2003/G9P[8]** | **DQ870502** | **87.3** | **86.7** | **87.3** |
|  | **C1** | **RVA/Human-wt/BEL/BE00038/2008/G1P[8]** | **HQ392341** | **86.9** | **86.4** | **87.0** |
|  | **C1** | **RVA/Human-wt/HUN/BP1227/2002/G4P[6]** | **KF835899** | **87.9** | **86.8** | **88.1** |
|  | **C1** | **RVA/Human-wt/DEU/GER126-08/2008/G12P[8]** | **FJ747614** | **87.3** | **86.9** | **87.6** |
|  | **C1** | **RVA/Human-wt/HUN/BP1490/1994/G4P[6]** | **KF835901** | **87.6** | **87.0** | **87.6** |
|  | **C1** | **RVA/Human-wt/IND/61060/2006/G1P[8]** | **HQ609557** | **87.4** | **87.3** | **87.4** |
|  | **C1** | **RVA/Human-tc/CHN/Y128/2004/G1P[8]** | **JQ087424** | **87.5** | **87.1** | **87.5** |
|  | **C1** | **RVA/Human-wt/AUS/CK00005/2004/G1P[8]** | **JF490134** | **87.2** | **86.7** | **87.3** |
|  | **C1** | **RVA/Human-wt/BGD/Dhaka16/2003/G1P[8]** | **DQ492670** | **87.1** | **86.8** | **87.3** |
|  | **C1** | **RVA/Human-wt/ZWE/MRC-DPRU1723/2009/G9P[8]** | **JN605416** | **87.0** | **86.8** | **87.1** |
|  | **C1** | **RVA/Human-wt/ITA/AV21/2010/G9P[8]** | **JX195064** | **87.1** | **86.8** | **87.1** |
|  | C2 | RVA/Human-tc/USA/DS-1/1976/G2P1B[4] | HQ650117 | 79.3 | 78.9 | 79.1 |
|  | C3 | RVA/Rhesus-tc/USA/TUCH/2002/G3P[24] | EF583011 | 80.2 | 79.3 | 79.9 |
|  | C4 | RVA/Pigeon-tc/JPN/PO-13/1983/G18P[17] | AB009630 | 68.8 | 69.1 | 68.6 |
|  | C5 | RVA/Human-tc/KEN/B10/1987/G3P[2] | HM627554 | 81.0 | 81.3 | 81.6 |
|  | C6 | RVA/Chicken-tc/DEU/02V0002G3/2002/G19P[30] | FJ169854 | 71.1 | 71.0 | 71.3 |
|  | C7 | RVA/Mouse-tc/USA/ETD_822/XXXX/G16P[16] | GQ479948 | 75.2 | 75.2 | 74.6 |
|  | C8 | RVA/Bat-wt/KEN/KE4852/07/2007/G25P[6] | GU983673 | 78.3 | 78.0 | 78.2 |
|  | C9 | RVA/Horse-tc/GBR/L338/1991/G13P[18] | JF712556 | 79.4 | 78.5 | 79.6 |
|  | C10 | RVA/SugarGlider-tc/JPN/SG385/2012/G27P[36] | AB971761 | 79.1 | 78.8 | 78.9 |
|  | C11 | RVA/Rat-wt/GER/KS-11-573/2011/G3P[3] | KJ879449 | 77.6 | 77.5 | 77.6 |
|  | C12 | RVA/Hu/ITA/ME848-12/2012/G12P[8] | KR632624 | 79.4 | 79.8 | 79.3 |
|  | C14 | RVA/Common_Gull-wt/JPN/Ho374/2013/G28P[39] | LC088219 | 70.8 | 70.6 | 70.6 |
| VP3 | ***M1*** | ***RVA/Pig-tc/KOR/PRG942/2006/G9P[23]*** | ***MF940483*** | 75.8 | **100.0** | **86.6** |
|  | ***M1*** | ***RVA/Pig-tc/KOR/PRG942V-20/2006/G9P[23]*** | ***MF940484*** | 75.7 | **99.4** | **86.5** |
|  | ***M1*** | ***RVA/Pig-tc/KOR/PRG942V-40/2006/G9P[23]*** | ***MF940485*** | 75.8 | **100.0** | **86.6** |
|  | ***M1*** | ***RVA/Pig-tc/KOR/PRG942V-60/2006/G9P[23]*** | ***MF940486*** | 75.8 | **99.5** | **86.3** |
|  | ***M1*** | ***RVA/Pig-tc/KOR/PRG942V-80/2006/G9P[23]*** | ***MF940487*** | 75.4 | **98.9** | **86.2** |
|  | ***M1*** | ***RVA/Pig-tc/KOR/K71/2006/G5P[7]*** | ***MF940428*** | 75.7 | **86.6** | **100.0** |
|  | ***M1*** | ***RVA/Pig-tc/KOR/K71V-20/2006/G5P[7]*** | ***MF940429*** | 75.7 | **86.4** | **99.8** |
|  | ***M1*** | ***RVA/Pig-tc/KOR/K71V-40/2006/G5P[7]*** | ***MF940430*** | 75.5 | **86.4** | **99.5** |
|  | ***M1*** | ***RVA/Pig-tc/KOR/K71V-60/2006/G5P[7]*** | ***MF940431*** | 75.5 | **86.4** | **99.5** |
|  | ***M1*** | ***RVA/Pig-tc/KOR/K71-80/2006/G5P[7]*** | ***MF940432*** | 75.6 | **86.4** | **99.7** |
|  | **M1** | **RVA/Cow-tc/KOR/K5/2004/G5P[7]** | **JX971571** | 75.8 | **86.7** | **99.8** |
|  | **M1** | **RVA/Human-tc/USA/Wa/1974/G1P1A[8]** | **AY267335** | 75.9 | **87.0** | **89.7** |
|  | **M1** | **RVA/Panda-tc/CHN/CH-1/2008/G1P[7]** | **HQ641295** | 75.7 | **95.6** | **86.6** |
|  | **M1** | **RVA/Pig-tc/USA/Gottfried/1983/G4P[6]** | **GU199488** | 75.3 | **86.5** | **89.9** |
|  | **M1** | **RVA/Pig-tc/USA/OSU/1977/G5P9[7]** | **AY277921** | 75.9 | **86.8** | **99.5** |
|  | **M1** | **RVA/Horse-tc/GBR/H-1/1975/G5P[7]** | **JQ309140** | 75.8 | **87.6** | **92.8** |
|  | **M1** | **RVA/Pig-tc/VEN/A131/1988/G3P9[7]** | **EF560620** | 74.4 | **84.5** | **96.5** |
|  | **M1** | **RVA/Pig-tc/VEN/A253/1988/G11P9[7]** | **EF560623** | 74.2 | **84.6** | **96.3** |
|  | **M1** | **RVA/Pig-tc/MEX/YM/1983/G11P9[7]** | **AY300922** | 75.4 | **87.0** | **92.3** |
|  | **M1** | **RVA/Pig-tc/KOR/PRG9121/2006/G9P[7]** | **JF796736** | 75.7 | **86.1** | **88.0** |
|  | **M1** | **RVA/Pig-tc/KOR/PRG9235/2006/G9P[23]** | **JF796703** | 75.9 | **99.6** | **86.8** |
|  | **M1** | **RVA/Pig-tc/KOR/PRG921/2006/G9P[23]** | **JF796714** | 75.8 | **99.5** | **86.7** |
|  | **M1** | **RVA/Pig-wt/IND/RU172/2002/G12P[7]** | **GU199193** | 75.6 | **88.6** | **86.9** |
|  | **M1** | **RVA/Human-wt/USA/DC1208-Bethesda/1980/G4P[8]** | **HM773856** | 75.9 | **85.9** | **88.6** |
|  | **M1** | **RVA/Human-wt/USA/DC4608-Bethesda/1980/G4P[8]** | **HM773900** | 76.0 | **86.0** | **88.6** |
|  | **M1** | **RVA/Human-tc/USA/WI61/1983/G9P1A[8]** | **EF583051** | 75.8 | **85.8** | **88.4** |
|  | **M1** | **RVA/Human-wt/BEL/B3458/2003/G9P[8]** | **DQ870503** | 76.1 | **86.0** | **87.8** |
|  | **M1** | **RVA/Human-wt/BEL/BE00038/2008/G1P[8]** | **HQ392343** | 75.8 | **86.0** | **87.8** |
|  | **M1** | **RVA/Human-wt/USA/DC1497-Bethesda/1976/G3P[8]** | **FJ947342** | 75.7 | **87.0** | **89.5** |
|  | **M1** | **RVA/Human-wt/USA/DC2106-Bethesda/1976/G3P[8]** | **FJ947839** | 75.9 | **87.2** | **89.6** |
|  | **M1** | **RVA/Human-wt/BEL/BE2001/2008/G9P[6]** | **JQ993323** | 76.1 | **87.0** | **90.4** |
|  | ***M2*** | ***RVA/Pig-tc/KOR/174-1/2006/G8P[7]*** | ***MF940538*** | **100.0** | 75.8 | 75.7 |
|  | ***M2*** | ***RVA/Pig-tc/KOR/174-1V-20/2006/G8P[7]*** | ***MF940539*** | **100.0** | 75.8 | 75.7 |
|  | ***M2*** | ***RVA/Pig-tc/KOR/174-1V-40/2006/G8P[7]*** | ***MF940540*** | **100.0** | 75.8 | 75.7 |
|  | ***M2*** | ***RVA/Pig-tc/KOR/174-1V-60/2006/G8P[7]*** | ***MF940541*** | **100.0** | 75.9 | 75.8 |
|  | ***M2*** | ***RVA/Pig-tc/KOR/174-1V-80/2006/G8P[7]*** | ***MF940542*** | **100.0** | 75.8 | 75.7 |
|  | **M2** | **RVA/Pig-tc/KOR/156-1/2006/G8P[7]** | **KF500198** | **98.9** | 75.4 | 75.3 |
|  | **M2** | **RVA/Pig-tc/KOR/C-1/2006/G8P[7]** | **KF500220** | **91.3** | 79.5 | 80.9 |
|  | **M2** | **RVA/Human-tc/USA/DS-1/1976/G2P1B[4]** | **EF990693** | **84.4** | 77.2 | 76.6 |
|  | **M2** | **RVA/Human-tc/KEN/B12/1987/G8P[1]** | **HM627544** | **88.1** | 75.4 | 75.2 |
|  | **M2** | **RVA/Cow-tc/USA/NCDV/1967/G6P6[1]** | **DQ870495** | **95.3** | 75.8 | 75.7 |
|  | **M2** | **RVA/Cow-tc/GBR/UK/1973/G6P7[5]** | **JF693050** | **95.3** | 75.6 | 76.2 |
|  | **M2** | **RVA/Cow-tc/FRA/RF/1982/G6P[1]** | **AY116592** | **95.2** | 75.8 | 75.6 |
|  | **M2** | **RVA/Cow-tc/VEN/BRV033/1990/G6P6[1]** | **EF560614** | **92.9** | 74.5 | 74.6 |
|  | **M2** | **RVA/Cow-tc/USA/WC3/1981/G6P[5]** | **EF560617** | **93.2** | 76.0 | 75.4 |
|  | **M2** | **RVA/Cow-wt/ARG/B383/1998/G15P[11]** | **FJ347113** | **85.7** | 75.4 | 74.2 |
|  | **M2** | **RVA/Cow-wt/JPN/Tottori-SG/2013/G15P[14]** | **AB853892** | **92.3** | 76.2 | 75.3 |
|  | **M2** | **RVA/Cow-tc/KOR/KJ19-2/2004/G8P[7]** | **HM988963** | **93.6** | 75.4 | 75.7 |
|  | **M2** | **RVA/Cow-tc/KOR/KJ9-1/2006/G6P[7]** | **HM988964** | **94.3** | 75.6 | 76.0 |
|  | **M2** | **RVA/Cow-tc/KOR/KJ25-1/2006/G8P[7]** | **HM988962** | **95.1** | 76.0 | 76.3 |
|  | **M2** | **RVA/Vaccine/USA/RotaTeq-WI79-4/1992/G6P1A[8]** | **GU565043** | **94.2** | 75.2 | 75.3 |
|  | **M2** | **RVA/Human-tc/USA/Se584/1998/G6P[9]** | **EF583043** | **83.6** | 76.9 | 76.6 |
|  | **M2** | **RVA/Human-wt/BRA/MS11142/2005/G2P[4]** | **KJ721717** | **83.5** | 77.0 | 76.7 |
|  | **M2** | **RVA/Human-wt/BRA/SE13801/2007/G2P[4]** | **KJ721714** | **90.2** | 75.9 | 75.8 |
|  | **M2** | **RVA/Human-wt/BEL/B1711/2002/G6P[6]** | **EF554084** | **83.4** | 76.7 | 76.5 |
|  | **M2** | **RVA/Human/JPN/S13-45/2013/G3P[4]** | **KJ639025** | **83.6** | 77.0 | 76.5 |
|  | **M2** | **RVA/Human-wt/ITA/PA150/2006/G2P[4]** | **KC178784** | **93.1** | 76.0 | 75.4 |
|  | **M2** | **RVA/Pig-tc/THA/P343/1991/G10P[5]** | **AB972858** | **91.8** | 76.0 | 75.5 |
|  | **M2** | **RVA/Human-wt/USA/VU10-11-19/2011/G2P[4]** | **KF716384** | **83.5** | 76.9 | 75.9 |
|  | **M2** | **RVA/Human-wt/AUS/CK20001/1977/G2P[4]** | **KC443589** | **84.3** | 77.0 | 76.4 |
|  | **M2** | **RVA/Human-tc/JPN/AU64/1989/G1P[4]** | **AB971557** | **83.5** | 76.4 | 76.2 |
|  | **M2** | **RVA/Human-tc/BRA/R49/1997/G1P[9]** | **KJ820838** | **83.3** | 76.4 | 76.4 |
|  | **M2** | **RVA/Guanaco-wt/ARG/Rio_Negro/1998/G8P[1]** | **FJ347124** | **93.1** | 75.9 | 75.6 |
|  | **M2** | **RVA/Cat-wt/ITA/BA222/2005/G3P[9]** | **GU827408** | **90.3** | 75.9 | 75.5 |
|  | M3 | RVA/Horse-wt/ARG/E403/2006/G14P[12] | JF712579 | 76.3 | 78.5 | 78.2 |
|  | M4 | RVA/Pigeon-tc/JPN/PO-13/1983/G18P[17] | AB009631 | 61.8 | 61.2 | 61.2 |
|  | M5 | RVA/Human-tc/KEN/B10/1987/G3P[2] | HM627555 | 76.7 | 76.8 | 76.9 |
|  | M6 | RVA/Horse-tc/GBR/L338/1991/G13P[18] | JF712557 | 75.5 | 78.0 | 77.9 |
|  | M7 | RVA/Chicken-tc/DEU/02V0002G3/2002/G19P[30] | FJ169855 | 62.9 | 62.2 | 62.4 |
|  | M8 | RVA/Mouse-tc/USA/ETD_822/XXXX/G16P[16] | GQ479949 | 67.5 | 67.3 | 66.9 |
|  | M9 | RVA/SugarGlider-tc/JPN/SG385/2012/G27P[36] | AB971762 | 70.0 | 71.1 | 71.3 |
|  | M10 | RVA/Rat-wt/GER/KS-11-573/2011/G3P[3] | KJ879450 | 74.8 | 74.5 | 75.0 |
|  | M11 | RVA/Hu/ITA/ME848-12/2012/G12P[8] | KR632625 | 75.7 | 76.8 | 77.7 |
|  | M13 | RVA/Common_Gull-wt/JPN/Ho374/2013/G28P[39] | LC088220 | 62.4 | 62.5 | 62.3 |
| **NSP1** | ***A1*** | ***RVA/Pig-tc/KOR/174-1/2006/G8P[7]*** | ***MF940558*** | **100.0** | 77.2 | **99.6** |
|  | ***A1*** | ***RVA/Pig-tc/KOR/174-1V-20/2006/G8P[7]*** | ***MF940559*** | **100.0** | 77.2 | **99.6** |
|  | ***A1*** | ***RVA/Pig-tc/KOR/174-1V-40/2006/G8P[7]*** | ***MF940560*** | **100.0** | 77.2 | **99.6** |
|  | ***A1*** | ***RVA/Pig-tc/KOR/174-1V-60/2006/G8P[7]*** | ***MF940561*** | **99.5** | 76.9 | **99.0** |
|  | ***A1*** | ***RVA/Pig-tc/KOR/174-1V-80/2006/G8P[7]*** | ***MF940562*** | **99.5** | 76.9 | **99.0** |
|  | ***A1*** | ***RVA/Pig-tc/KOR/K71/2006/G5P[7]*** | ***MF940448*** | **99.6** | 77.1 | **100.0** |
|  | ***A1*** | ***RVA/Pig-tc/KOR/K71V-20/2006/G5P[7]*** | ***MF940449*** | **99.6** | 77.1 | **100.0** |
|  | ***A1*** | ***RVA/Pig-tc/KOR/K71V-40/2006/G5P[7]*** | ***MF940450*** | **99.6** | 77.1 | **100.0** |
|  | ***A1*** | ***RVA/Pig-tc/KOR/K71V-60/2006/G5P[7]*** | ***MF940451*** | **99.6** | 77.1 | **100.0** |
|  | ***A1*** | ***RVA/Pig-tc/KOR/K71-80/2006/G5P[7]*** | ***MF940452*** | **99.6** | 77.1 | **100.0** |
|  | **A1** | **RVA/Pig-tc/KOR/156-1/2006/G8P[7]** | **KF500202** | **98.8** | 77.3 | **98.7** |
|  | **A1** | **RVA/Pig-tc/USA/OSU/1977/G5P[7]** | **U08432** | **98.8** | 76.7 | **98.5** |
|  | **A1** | **RVA/Pig-tc/VEN/A253/1988/G11P[7]** | **EF990695** | **95.5** | 75.8 | **95.3** |
|  | **A1** | **RVA/Pig-tc/VEN/A131/1988/G3P[7]** | **EF990687** | **93.5** | 74.6 | **93.2** |
|  | **A1** | **RVA/Cow-tc/KOR/K5/2004/G5P[7]** | **JX971575** | **99.9** | 77.2 | **99.7** |
|  | **A1** | **RVA/Cow-tc/KOR/KJ25-1/2006/G8P[7]** | **FJ206198** | **99.9** | 77.2 | **99.7** |
|  | **A1** | **RVA/Cow-tc/KOR/KJ75/2004/G5P[5]** | **DQ494395** | **99.9** | 77.2 | **99.7** |
|  | **A1** | **RVA/Cow-tc/KOR/KJ9-1/2006/G6P[7]** | **FJ206224** | **99.4** | 77.0 | **99.1** |
|  | **A1** | **RVA/Cow-tc/KOR/K8/2005/G5P[7]** | **EU542703** | **99.9** | 77.1 | **99.6** |
|  | **A1** | **RVA/Panda-tc/CHN/CH-1/2008/G1P[7]** | **GU205762** | **99.9** | 77.2 | **99.6** |
|  | **A1** | **RVA/Human-tc/USA/WI61/1983/G9P1A[8]** | **EF672620** | **92.2** | 78.1 | **92.0** |
|  | **A1** | **RVA/Human-wt/USA/DC2106-Bethesda/1976/G3P[8]** | **FJ947841** | **92.5** | 78.0 | **92.2** |
|  | **A1** | **RVA/Human-wt/USA/DC1497-Bethesda/1976/G3P[8]** | **FJ947344** | **92.4** | 77.8 | **92.1** |
|  | **A1** | **RVA/Human-tc/USA/Wa/1974/G1P1A[8]** | **JX406751** | **85.0** | 77.5 | **84.9** |
|  | **A1** | **RVA/Human-wt/BEL/B3458/2003/G9P[8]** | **EF990709** | **84.4** | 78.5 | **84.1** |
|  | **A1** | **RVA/Human-wt/BEL/BE00038/2008/G1P[8]** | **HQ392334** | **83.9** | 78.4 | **83.6** |
|  | **A1** | **RVA/Human-wt/USA/DC1208-Bethesda/1980/G4P[8]** | **HM773858** | **84.6** | 77.6 | **84.3** |
|  | **A1** | **RVA/Human-wt/USA/DC4608-Bethesda/1980/G4P[8]** | **HM773902** | **84.8** | 77.8 | **84.5** |
|  | **A1** | **RVA/Human-wt/IND/61060/2006/G1P[8]** | **HQ609566** | **84.0** | 79.0 | **83.7** |
|  | **A1** | **RVA/Human-wt/USA/DC2314/1976/G1P[8]** | **KC579548** | **84.9** | 77.9 | **84.6** |
|  | **A1** | **RVA/Human-wt/ITA/AV21/2010/G9P[8]** | **JX195069** | **84.4** | 79.1 | **84.2** |
|  | **A1** | **RVA/Human-tc/CHN/Y128/2004/G1P[8]** | **JQ087429** | **85.3** | 77.4 | **85.3** |
|  | **A1** | **RVA/Human-wt/USA/DC1476/1974/G1P[8]** | **KC579559** | **85.0** | 77.7 | **84.8** |
|  | **A1** | **RVA/Human-wt/USA/DC827/1978/G4P[8]** | **HM773957** | **85.0** | 77.7 | **84.7** |
|  | **A1** | **RVA/Human-wt/AUS/CK00005/2004/G1P[8]** | **JF490129** | **84.8** | 78.7 | **84.5** |
|  | **A1** | **RVA/Human-wt/Bel/BE00055/1999/G1P[8]** | **JN258801** | **84.5** | 78.7 | **84.2** |
|  | **A1** | **RVA/Human-wt/DEU/GER126-08/1974/G12P[8]** | **FJ747620** | **84.4** | 79.1 | **84.2** |
|  | **A1** | **RVA/Human-wt/BEL/B4633/2003/G12P[8]** | **DQ146644** | **84.3** | 78.5 | **84.0** |
|  | **A1** | **RVA/Human-wt/ZWE/MRC-DPRU1723/2009/G9P[8]** | **JN605421** | **84.2** | 78.8 | **84.0** |
|  | **A1** | **RVA/Human-wt/BEL/BE00043/2009/G1P[8]** | **HQ392379** | **84.2** | 78.8 | **83.9** |
|  | **A1** | **RVA/Human-wt/USA/DC4320/1988/G4P[8]** | **HM773891** | **84.2** | 77.5 | **84.0** |
|  | **A1** | **RVA/Human-wt/ZAF/MRC-DPRU1262/2004/G1P[8]** | **KP752737** | **84.0** | 78.8 | **83.7** |
|  | A2 | RVA/Human-tc/USA/DS-1/1976/G2P1B[4] | EF672578 | 74.8 | 74.3 | 74.7 |
|  | A2 | RVA/Human-wt/COD/DRC88/2003/G8P8 | DQ005108 | 74.4 | 75.2 | 74.3 |
|  | A3 | RVA/Human-tc/USA/Se584/1998/G6P[9] | EF672606 | 64.4 | 65.5 | 64.5 |
|  | A3 | RVA/Cow-tc/GBR/UK/1973/G6P7[5] | EF990703 | 65.2 | 65.1 | 65.2 |
|  | A3 | RVA/Human-tc/KEN/B12/1987/G8P[1] | HM627548 | 66.0 | 67.5 | 66.0 |
|  | A3 | RVA/Cow-tc/VEN/BRV033/1990/G6P[6] | EF990703 | 64.9 | 67.0 | 64.9 |
|  | A5 | RVA/Human-tc/KEN/B10/1987/G3P[2] | HM627559 | 51.6 | 51.2 | 51.5 |
|  | A6 | RVA/Horse-tc/GBR/L338/1991/G13P[18] | JF712561 | 51.1 | 51.0 | 50.8 |
|  | A7 | RVA/Mouse-tc/XXX/EHP/1981/G16P[20] | U08423 | 49.0 | 49.6 | 48.8 |
|  | ***A8*** | ***RVA/Pig-tc/KOR/PRG942/2006/G9P[23]*** | ***MF940503*** | 77.2 | **100.0** | 77.1 |
|  | **A8** | ***RVA/Pig-tc/KOR/PRG942V-20/2006/G9P[23]*** | ***MF940504*** | 77.3 | **99.7** | 77.2 |
|  | ***A8*** | ***RVA/Pig-tc/KOR/PRG942V-40/2006/G9P[23]*** | ***MF940505*** | 77.1 | **99.5** | 77.0 |
|  | **A8** | ***RVA/Pig-tc/KOR/PRG942V-60/2006/G9P[23]*** | ***MF940506*** | 77.1 | **99.5** | 77.0 |
|  | ***A8*** | ***RVA/Pig-tc/KOR/PRG942V-80/2006/G9P[23]*** | ***MF940507*** | 77.1 | **99.5** | 77.0 |
|  | **A8** | **RVA/Pig-tc/KOR/C-1/2006/G8P[7]** | **KF500224** | 76.9 | **86.8** | 76.9 |
|  | **A8** | **RVA/Pig-tc/MEX/YM/1983/G11P[7]** | **D38154** | 76.9 | **91.8** | 76.7 |
|  | **A8** | **RVA/Pig-tc/KOR/PRG9235/2006/G9P[23]** | **JF796696** | 77.2 | **99.7** | 77.1 |
|  | **A8** | **RVA/Pig-tc/KOR/PRG921/2006/G9P[23]** | **JF796707** | 75.5 | **88.5** | 75.4 |
|  | **A8** | **RVA/Pig-tc/KOR/PRG9121/2006/G9P[7]** | **JF796729** | 75.4 | **88.4** | 75.2 |
|  | **A8** | **RVA/Pig-tc/USA/Gottfried/1983/G4P[6]** | **U08431** | 76.5 | **87.4** | 76.5 |
|  | **A8** | **RVA/Horse-tc/GBR/H-1/1975/G5P[7]** | **JQ309141** | 77.2 | **91.0** | 77.0 |
|  | **A8** | **RVA/Human-wt/HUN/BP1125/2004/G4P[6]** | **KF835938** | 76.8 | **87.2** | 76.8 |
|  | **A8** | **RVA/Human-wt/PRY/1809SR/2009/G4P[6]** | **KJ412559** | 78.9 | **87.8** | 78.7 |
|  | **A8** | **RVA/Human-wt/HUN/BP271/2000/G4P[6]** | **KF835937** | 76.5 | **87.4** | 76.4 |
|  | **A8** | **RVA/Human-xx/IND/mani-253/2007/G4P[4]** | **HM348717** | 76.5 | **85.5** | 76.5 |
|  | **A8** | **RVA/Human-wt/JPN/Ryukyu-1120/2011/G5P[6]** | **AB741655** | 77.3 | **87.0** | 77.3 |
|  | **A8** | **RVA/Human-xx/IND/mcs-10/2007/G9P[6]** | **FJ154080** | 76.2 | **84.7** | 76.2 |
|  | **A8** | **RVA/Human-wt/IND/mcs-13-07/2007/G9P[6]** | **FJ154083** | 76.2 | **84.7** | 76.2 |
|  | **A8** | **RVA/Human-xx/IND/mani-362/2007/G4P[6]** | **HM348719** | 76.5 | **86.3** | 76.5 |
|  | **A8** | **RVA/Human-wt/ARG/Arg4605/2006/G4P[6]** | **KC412033** | 78.8 | **87.8** | 78.8 |
|  | **A8** | **RVA/Human-wt/BEL/BE2001/2008/G9P[6]** | **JQ993324** | 76.3 | **86.6** | 76.2 |
|  | **A8** | **RVA/Human-xx/IND/mani-97/2006/G9P[19]** | **HM348716** | 77.3 | **85.4** | 77.3 |
|  | **A8** | **RVA/Human-wt/THA/Mc323/1989/G9P[19]** | **JN104615** | 77.3 | **87.4** | 77.3 |
|  | A9 | RVA/Dog-tc/USA/CU-1/1982/G3P[3] | EU708918 | 51.6 | 52.5 | 51.6 |
|  | A10 | RVA/Horse-wt/ARG/E403/2006/G14P[12] | JF712583 | 50.6 | 49.9 | 50.5 |
|  | A11 | RVA/Human-wt/HUN/Hun5/1997/G6P[14] | EF554110 | 64.5 | 65.5 | 64.4 |
|  | A12 | RVA/Human-tc/THA/T152/1998/G12P[9] | AB097459 | 59.4 | 59.1 | 59.4 |
|  | A13 | RVA/Cow-xx/ARG/B383/1998/G15P[11] | FJ347117 | 65.2 | 66.2 | 65.1 |
|  | A14 | RVA/Cow-tc/THA/A5-13/XXXX/G8P[1] | D38148 | 65.5 | 67.0 | 65.3 |
|  | A15 | RVA/Human-tc/ITA/260-97/1997/G3P[3] | HQ661118 | 58.3 | 57.2 | 58.3 |
|  | A16 | RVA/Chicken-tc/DEU/02V0002G3/2002/G19P[30] | FJ169857 | 39.9 | 39.9 | 39.8 |
|  | A18 | RVA/Camel-wt/SDN/MRC-DPRU447/2004/G8P[11] | KC257086 | 66.1 | 66.3 | 66.3 |
|  | A19 | RVA/Human-wt/BRA/QUI-35-F5/2010/G3P[9] | KF185099 | 58.9 | 59.3 | 58.9 |
|  | A20 | RVA/SugarGlider-tc/JPN/SG385/2012/G27P[36] | AB971766 | 55.1 | 56.5 | 55.2 |
|  | A21 | RVA/VelvetScoter-tc/JPN/RK1/1989/G18P[17] | LC088102 | 38.3 | 38.6 | 38.3 |
|  | A22 | RVA/Rat-wt/GER/KS-11-573/2011/G3P[3] | KJ879454 | 49.1 | 48.0 | 49.1 |
|  | A24 | RVA/Common_Gull-wt/JPN/Ho374/2013/G28P[39] | LC088224 | 38.7 | 40.6 | 38.7 |
| NSP2 | ***N1*** | ***RVA/Pig-tc/KOR/174-1/2006/G8P[7]*** | ***MF940563*** | **100.0** | **91.8** | **99.4** |
|  | ***N1*** | ***RVA/Pig-tc/KOR/174-1V-20/2006/G8P[7]*** | ***MF940564*** | **99.7** | **91.9** | **99.7** |
|  | ***N1*** | ***RVA/Pig-tc/KOR/174-1V-40/2006/G8P[7]*** | ***MF940565*** | **99.7** | **91.9** | **99.7** |
|  | ***N1*** | ***RVA/Pig-tc/KOR/174-1V-60/2006/G8P[7]*** | ***MF940566*** | **99.7** | **91.9** | **99.7** |
|  | ***N1*** | ***RVA/Pig-tc/KOR/174-1V-80/2006/G8P[7]*** | ***MF940567*** | **99.7** | **91.9** | **99.7** |
|  | ***N1*** | ***RVA/Pig-tc/KOR/PRG942/2006/G9P[23]*** | ***MF940508*** | **91.8** | **100.0** | **91.6** |
|  | ***N1*** | ***RVA/Pig-tc/KOR/PRG942V-20/2006/G9P[23]*** | ***MF940509*** | **93.1** | **98.2** | **92.9** |
|  | ***N1*** | ***RVA/Pig-tc/KOR/PRG942V-40/2006/G9P[23]*** | ***MF940510*** | **92.9** | **98.0** | **92.7** |
|  | ***N1*** | ***RVA/Pig-tc/KOR/PRG942V-60/2006/G9P[23]*** | ***MF940511*** | **92.8** | **97.9** | **92.6** |
|  | ***N1*** | ***RVA/Pig-tc/KOR/PRG942V-80/2006/G9P[23]*** | ***MF940512*** | **92.6** | **98.1** | **92.3** |
|  | ***N1*** | ***RVA/Pig-tc/KOR/K71/2006/G5P[7]*** | ***MF940453*** | **99.4** | **91.6** | **100.0** |
|  | ***N1*** | ***RVA/Pig-tc/KOR/K71V-20/2006/G5P[7]*** | ***MF940454*** | **99.4** | **91.6** | **99.6** |
|  | ***N1*** | ***RVA/Pig-tc/KOR/K71V-40/2006/G5P[7]*** | ***MF940455*** | **93.0** | **98.1** | **92.8** |
|  | ***N1*** | ***RVA/Pig-tc/KOR/K71V-60/2006/G5P[7]*** | ***MF940456*** | **99.3** | **91.5** | **99.5** |
|  | ***N1*** | ***RVA/Pig-tc/KOR/K71-80/2006/G5P[7]*** | ***MF940457*** | **99.3** | **91.5** | **99.5** |
|  | **N1** | **RVA/Pig-tc/KOR/156-1/2006/G8P[7]** | **KF500203** | **93.3** | **94.5** | **93.5** |
|  | **N1** | **RVA/Pig-tc/KOR/C-1/2006/G8P[7]** | **KF500225** | **99.6** | **91.8** | **99.6** |
|  | **N1** | **RVA/Pig-tc/MEX/YM/1983/G11P[7]** | **GU199517** | **93.0** | **93.9** | **93.2** |
|  | **N1** | **RVA/Pig-tc/KOR/PRG921/2006/G9P[23]** | **JF796708** | **93.2** | **98.1** | **93.0** |
|  | **N1** | **RVA/Pig-tc/USA/Gottfried/1983/G4P[6]** | **GU199489** | **97.0** | **92.7** | **97.0** |
|  | **N1** | **RVA/Pig-tc/USA/OSU/1977/G5P[7]** | **X06722** | **99.4** | **91.7** | **99.4** |
|  | **N1** | **RVA/Pig-tc/VEN/A253/1988/G11P[7]** | **EF990696** | **94.5** | **87.8** | **94.5** |
|  | **N1** | **RVA/Pig-tc/VEN/A131/1988/G3P[7]** | **EF990688** | **91.7** | **85.2** | **91.7** |
|  | **N1** | **RVA/Pig-wt/IND/RU172/2002/G12P[7]** | **GU199195** | **87.8** | **86.0** | **87.8** |
|  | **N1** | **RVA/Pig-tc/KOR/PRG9121/2006/G9P[7]** | **JF796730** | **87.3** | **85.7** | **87.4** |
|  | **N1** | **RVA/Pig-tc/KOR/PRG9235/2006/G9P[23]** | **JF796697** | **87.3** | **85.7** | **87.4** |
|  | **N1** | **RVA/Cow-tc/KOR/KJ246/2006/G8P[7]** | **FJ206155** | **99.7** | **91.9** | **99.7** |
|  | **N1** | **RVA/Cow-tc/KOR/KJ330-1/2006/G8P[7]** | **FJ206158** | **99.7** | **91.9** | **99.7** |
|  | **N1** | **RVA/Cow-tc/KOR/KJ338-1/2006/G8P[7]** | **FJ206160** | **99.7** | **91.9** | **99.7** |
|  | **N1** | **RVA/Cow-tc/KOR/K8/2005/G5P[7]** | **EU542709** | **99.7** | **91.9** | **99.7** |
|  | **N1** | **RVA/Cow-tc/KOR/KJ44/2004/G5P[1]** | **DQ494401** | **99.7** | **91.9** | **99.7** |
|  | **N1** | **RVA/Cow-tc/KOR/KJ75/2004/G5P[5]** | **DQ494402** | **99.7** | **91.9** | **99.7** |
|  | **N1** | **RVA/Cow-tc/KOR/KJ25-1/2006/G8P[7]** | **FJ206120** | **99.7** | **91.9** | **99.7** |
|  | **N1** | **RVA/Cow-tc/KOR/K5/2004/G5P[7]** | **JX971576** | **99.6** | **91.8** | **99.6** |
|  | **N1** | **RVA/Panda-tc/CHN/CH-1/2008/G1P[7]** | **GU188281** | **99.6** | **91.8** | **99.6** |
|  | **N1** | **RVA/Horse-tc/GBR/H-1/1975/G5P[7]** | **JQ309142** | **87.8** | **86.3** | **87.8** |
|  | **N1** | **RVA/Human-wt/ECU/EC2184/200x/G11P[6]** | **GQ149101** | **92.3** | **93.6** | **92.1** |
|  | **N1** | **RVA/Human-wt/BEL/B3458/2003/G9P[8]** | **EF990710** | **92.3** | **93.6** | **92.1** |
|  | **N1** | **RVA/Human-wt/PRY/1809SR/2009/G4P[6]** | **KJ412560** | **91.6** | **93.8** | **91.8** |
|  | **N1** | **RVA/Human-wt/BEL/BE00038/2008/G1P[8]** | **HQ392333** | **92.0** | **92.0** | **92.4** |
|  | **N1** | **RVA/Human-wt//USA/DC1730/1979/G3P[8]** | **FJ947325** | **94.4** | **92.6** | **94.4** |
|  | **N1** | **RVA/Human-wt/USA/DC827/1978/G4P[8]** | **HM773960** | **93.6** | **92.0** | **93.8** |
|  | **N1** | **RVA/Human-wt/BEL/BE2001/2008/G9P[6]** | **JQ993325** | **92.8** | **92.0** | **92.8** |
|  | **N1** | **RVA/Human-wt/Bel/BE00055/1999/G1P[8]** | **JN258794** | **93.3** | **92.1** | **93.4** |
|  | **N1** | **RVA/Human-wt/ZAF/MRC-DPRU1262/2004/G1P[8]** | **KP752738** | **93.5** | **91.3** | **93.6** |
|  | **N1** | **RVA/Human-wt/BEL/BE00043/2009/G1P[8]** | **HQ392378** | **92.2** | **91.3** | **92.3** |
|  | **N1** | **RVA/Human-wt/BGD/Dhaka16/2003/G1P[8]** | **DQ492676** | **89.8** | **88.6** | **89.7** |
|  | **N1** | **RVA/Human-tc/CHN/Y128/2004/G1P[8]** | **JQ087430** | **89.5** | **88.7** | **89.4** |
|  | **N1** | **RVA/Human-wt/ZWE/MRC-DPRU1723/2009/G9P[8]** | **JN605422** | **89.3** | **88.2** | **89.2** |
|  | **N1** | **RVA/Human-xx/IND/mani-97/2006/G9P[19]** | **HM348720** | **89.4** | **88.2** | **89.3** |
|  | **N1** | **RVA/Human-wt/DEU/GER126-08/2008/G12P[8]** | **FJ747621** | **89.4** | **88.2** | **89.3** |
|  | **N1** | **RVA/Human-xx/IND/mani-362/2007/G4P[6]** | **HM348723** | **89.5** | **87.5** | **89.4** |
|  | **N1** | **RVA/Human-xx/IND/mani-253/2007/G3P[8]** | **HM348721** | **88.7** | **87.6** | **88.6** |
|  | **N1** | **RVA/Human-wt/IND/61060/2006/G1P[8]** | **HQ609569** | **89.3** | **87.9** | **89.2** |
|  | **N1** | **RVA/Human-wt/ITA/AV21/2010/G9P[8]** | **JX195070** | **89.6** | **88.2** | **89.5** |
|  | **N1** | **RVA/Human-tc/USA/WI61/1983/G9P1A[8]** | **EF672622** | **87.3** | **87.7** | **87.2** |
|  | **N1** | **RVA/Human-wt/AUS/CK00005/2004/G1P[8]** | **JF490128** | **88.7** | **87.4** | **88.6** |
|  | **N1** | **RVA/Human-wt/USA/DC1497-Bethesda/1976/G3P[8]** | **FJ947347** | **87.6** | **87.8** | **87.5** |
|  | **N1** | **RVA/Human-wt/USA/DC102/1974/G1P[8]** | **KC580001** | **89.6** | **87.7** | **89.5** |
|  | **N1** | **RVA/Human-wt/USA/DC1208-Bethesda/1980/G4P[8]** | **HM773861** | **87.7** | **87.9** | **87.6** |
|  | **N1** | **RVA/Human-wt/IND/mcs-13-07/2007/G9P[6]** | **EU753970** | **88.5** | **86.5** | **88.4** |
|  | **N1** | **RVA/Human-wt/USA/DC2314/1976/G1P[8]** | **KC579549** | **89.3** | **87.5** | **89.2** |
|  | **N1** | **RVA/Human-wt/BRA/HST327/1999/G4P[6]** | **JQ898159** | **89.3** | **88.0** | **89.2** |
|  | **N1** | **RVA/Human-tc/USA/Wa/1974/G1P1A[8]** | **L04534** | **87.6** | **87.5** | **87.5** |
|  | N2 | RVA/Human-tc/USA/DS-1/1976/G2P1B[4] | EF672580 | 83.1 | 81.9 | 83.1 |
|  | N2 | RVA/Cow-tc/KOR/KJ9-1/2006/G6P[7] | FJ206108 | 84.2 | 82.9 | 84.2 |
|  | N3 | RVA/Human-tc/THA/T152/1998/G12P[9] | DQ146703 | 80.5 | 78.9 | 81.0 |
|  | N4 | RVA/Pigeon-tc/JPN/PO-13/1983/G18P[17] | AB009625 | 61.0 | 59.3 | 61.0 |
|  | N5 | RVA/Human-tc/KEN/B10/1987/G3P[2] | HM627560 | 81.2 | 79.0 | 81.4 |
|  | N6 | RVA/Chicken-tc/DEU/02V0002G3/2002/G19P[30] | FJ169860 | 61.4 | 60.3 | 61.4 |
|  | N7 | RVA/Mouse-tc/USA/ETD_822/XXXX/G16P[16] | GQ479954 | 74.8 | 73.3 | 74.8 |
|  | N8 | RVA/Bat-wt/KEN/KE4852/07/2007/G25P[6] | GU983677 | 76.0 | 73.9 | 76.1 |
|  | N9 | RVA/Horse-tc/GBR/L338/1991/G13P[18] | JF712562 | 81.2 | 78.1 | 81.3 |
|  | N10 | RVA/Pheasant-tc/GER/10V0112H5/2010/G23P[37] | JX204818 | 62.9 | 61.8 | 62.7 |
|  | N11 | RVA/SugarGlider-tc/JPN/SG385/2012/G27P[36] | AB971767 | 78.5 | 77.5 | 78.3 |
|  | N12 | RVA/Hu/ITA/ME848-12/2012/G12P[8] | KR632627 | 81.4 | 79.2 | 81.3 |
|  | N14 | RVA/Common_Gull-wt/JPN/Ho374/2013/G28P[39] | LC088225 | 62.3 | 60.9 | 62.4 |
| NSP3 | ***T1*** | ***RVA/Pig-tc/KOR/174-1/2006/G8P[7]*** | ***MF940568*** | **100.0** | **87.3** | **99.7** |
|  | ***T1*** | ***RVA/Pig-tc/KOR/174-1V-20/2006/G8P[7]*** | ***MF940569*** | **100.0** | **87.3** | **99.7** |
|  | ***T1*** | ***RVA/Pig-tc/KOR/174-1V-40/2006/G8P[7]*** | ***MF940570*** | **100.0** | **87.3** | **99.7** |
|  | ***T1*** | ***RVA/Pig-tc/KOR/174-1V-60/2006/G8P[7]*** | ***MF940571*** | **100.0** | **87.3** | **99.7** |
|  | ***T1*** | ***RVA/Pig-tc/KOR/174-1V-80/2006/G8P[7]*** | ***MF940572*** | **100.0** | **87.3** | **99.7** |
|  | ***T1*** | ***RVA/Pig-tc/KOR/PRG942/2006/G9P[23]*** | ***MF940513*** | **87.3** | **100.0** | **87.1** |
|  | ***T1*** | ***RVA/Pig-tc/KOR/PRG942V-20/2006/G9P[23]*** | ***MF940514*** | **87.5** | **99.4** | **87.3** |
|  | ***T1*** | ***RVA/Pig-tc/KOR/PRG942V-40/2006/G9P[23]*** | ***MF940515*** | **87.5** | **99.4** | **87.3** |
|  | ***T1*** | ***RVA/Pig-tc/KOR/PRG942V-60/2006/G9P[23]*** | ***MF940516*** | **87.5** | **99.3** | **87.3** |
|  | ***T1*** | ***RVA/Pig-tc/KOR/PRG942V-80/2006/G9P[23]*** | ***MF940517*** | **87.3** | **99.2** | **87.1** |
|  | ***T1*** | ***RVA/Pig-tc/KOR/K71/2006/G5P[7]*** | ***MF940458*** | **99.7** | **87.1** | **100.0** |
|  | ***T1*** | ***RVA/Pig-tc/KOR/K71V-20/2006/G5P[7]*** | ***MF940459*** | **99.7** | **87.1** | **100.0** |
|  | ***T1*** | ***RVA/Pig-tc/KOR/K71V-40/2006/G5P[7]*** | ***MF940460*** | **99.7** | **87.1** | **100.0** |
|  | ***T1*** | ***RVA/Pig-tc/KOR/K71V-60/2006/G5P[7]*** | ***MF940461*** | **87.4** | **99.0** | **87.2** |
|  | ***T1*** | ***RVA/Pig-tc/KOR/K71-80/2006/G5P[7]*** | ***MF940462*** | **87.4** | **99.0** | **87.2** |
|  | ***T1*** | ***RVA/Pig-tc/KOR/156-1/2006/G8P[7]*** | ***KF500204*** | **88.5** | **93.0** | **88.3** |
|  | ***T1*** | ***RVA/Pig-tc/KOR/C-1/2006/G8P[7]*** | ***KF500226*** | **99.7** | **87.3** | **99.4** |
|  | **T1** | **RVA/Pig-tc/USA/OSU/1977/G5P[7]** | **X81431** | **100.0** | **87.4** | **99.7** |
|  | **T1** | **RVA/Pig-tc/USA/Gottfried/1983/G4P[6]** | **X81430** | **95.6** | **88.5** | **95.3** |
|  | **T1** | **RVA/Pig-tc/VEN/A253/1988/G11P[7]** | **EF990697** | **95.2** | **84.2** | **94.9** |
|  | **T1** | **RVA/Pig-tc/VEN/A131/1988/G3P[7]** | **EF990689** | **93.3** | **82.7** | **93.1** |
|  | **T1** | **RVA/Pig-tc/KOR/PRG9121/2006/G9P[7]** | **JF796731** | **87.5** | **93.6** | **87.3** |
|  | **T1** | **RVA/Pig-tc/KOR/PRG921/2006/G9P[23]** | **JF796709** | **87.6** | **99.5** | **87.4** |
|  | **T1** | **RVA/Pig-tc/KOR/PRG9235/2006/G9P[23]** | **JF796698** | **87.6** | **99.5** | **87.4** |
|  | **T1** | **RVA/Pig-wt/IND/RU172/2002/G12P[7]** | **GU199196** | **88.0** | **92.9** | **87.8** |
|  | **T1** | **RVA/Pig-tc/MEX/YM/1983/G11P[7]** | **GU199518** | **88.5** | **88.7** | **88.3** |
|  | **T1** | **RVA/Cow-tc/KOR/KJ330-1/2006/G8P[7]** | **FJ206192** | **100.0** | **87.4** | **99.7** |
|  | **T1** | **RVA/Cow-tc/KOR/K8/2005/G5P[7]** | **EU542715** | **100.0** | **87.4** | **99.7** |
|  | **T1** | **RVA/Cow-tc/KOR/KJ338-1/2006/G8P[7]** | **FJ206193** | **100.0** | **87.4** | **99.7** |
|  | **T1** | **RVA/Cow-tc/KOR/K5/2004/G5P[7]** | **JX971577** | **99.9** | **87.3** | **99.6** |
|  | **T1** | **RVA/Cow-tc/KOR/KJ44/2004/G5P[1]** | **DQ494403** | **99.9** | **87.3** | **99.6** |
|  | **T1** | **RVA/Cow-tc/KOR/KJ75/2004/G5P[5]** | **DQ494404** | **99.9** | **87.3** | **99.6** |
|  | **T1** | **RVA/Cow-tc/KOR/KJ246/2006/G8P[7]** | **FJ206190** | **99.8** | **87.2** | **99.5** |
|  | **T1** | **RVA/Cow-tc/KOR/KJ9-1/2006/G6P[7]** | **FJ206226** | **99.6** | **87.0** | **99.3** |
|  | **T1** | **RVA/Cow-tc/KOR/KJ25-1/2006/G8P[7]** | **FJ206169** | **99.0** | **86.5** | **98.7** |
|  | **T1** | **RVA/Panda-tc/CHN/CH-1/2008/G1P[7]** | **GU329525** | **99.9** | **87.3** | **99.6** |
|  | **T1** | **RVA/Horse-tc/GBR/H-1/1975/G5P[7]** | **JQ309143** | **97.2** | **87.9** | **96.9** |
|  | **T1** | **RVA/Human-wt/BEL/Mc323/199/G9P[19]** | **JN104617** | **89.1** | **93.8** | **88.9** |
|  | **T1** | **RVA/Human-wt/HUN/BP1227/2002/G4P[6]** | **KF835955** | **89.2** | **89.4** | **89.0** |
|  | **T1** | **RVA/Human-xx/IND/mani-362/2007/G4P[6]** | **HM348727** | **89.4** | **88.1** | **89.1** |
|  | **T1** | **RVA/Human-tc/USA/Wa/1974/G1P1A[8]** | **X81434** | **89.0** | **87.1** | **88.8** |
|  | **T1** | **RVA/Human-wt/BEL/BE00038/2008/G1P[8]** | **HQ392342** | **88.2** | **87.8** | **87.9** |
|  | **T1** | **RVA/Human-tc/USA/WI61/1983/G9P1A[8]** | **EF672621** | **88.3** | **86.7** | **88.0** |
|  | **T1** | **RVA/Human-wt/BEL/B3458/2003/G9P[8]** | **EF990711** | **88.3** | **85.9** | **88.0** |
|  | **T1** | **RVA/Human-wt/USA/DC1208-Bethesda/1980/G4P[8]** | **HM773860** | **88.8** | **89.0** | **88.5** |
|  | **T1** | **RVA/Human-wt/USA/DC4608-Bethesda/1980/G4P[8]** | **HM773904** | **88.9** | **88.9** | **88.6** |
|  | **T1** | **RVA/Human-wt/USA/DC2106-Bethesda/1976/G3P[8]** | **FJ947843** | **88.6** | **88.9** | **88.4** |
|  | **T1** | **RVA/Human-wt/USA/DC1497-Bethesda/1976/G3P[8]** | **FJ947346** | **88.5** | **87.6** | **88.3** |
|  | **T1** | **RVA/Human-xx/IND/mani-253/2007/G3P[8]** | **HM348725** | **87.7** | **86.8** | **87.5** |
|  | **T1** | **RVA/Human-wt/IND/mcs-13-07/2007/G9P[6]** | **EU753969** | **88.7** | **88.9** | **88.7** |
|  | **T1** | **RVA/Human-wt/ECU/EC2184/200x/G11P[6]** | **GQ149098** | **88.2** | **86.8** | **88.1** |
|  | **T1** | **RVA/Human-wt/BEL/BE00043/2009/G1P[8]** | **HQ392387** | **88.4** | **86.3** | **88.1** |
|  | **T1** | **RVA/Human-wt/IND/61060/2006/G1P[8]** | **HQ609572** | **88.6** | **86.5** | **88.3** |
|  | **T1** | **RVA/Human-wt/BEL/B4633/2003/G12P[8]** | **DQ146646** | **88.2** | **86.2** | **87.9** |
|  | **T1** | **RVA/Human-tc/CHN/Y128/2004/G1P[8]** | **JQ087431** | **88.6** | **86.8** | **88.3** |
|  | **T1** | **RVA/Human-wt/DEU/GER126-08/2008/G12P[8]** | **FJ747622** | **88.5** | **87.8** | **88.2** |
|  | **T1** | **RVA/Human-wt/USA/DC827/1978/G4P[8]** | **HM773959** | **88.2** | **87.6** | **87.8** |
|  | **T1** | **RVA/Human-wt/AUS/CK00005/2004/G1P[8]** | **JF490132** | **88.7** | **87.6** | **88.4** |
|  | **T1** | **RVA/Human-wt/ZAF/MRC-DPRU1262/2004/G1P[8]** | **KP752739** | **87.0** | **87.0** | **86.7** |
|  | **T1** | **RVA/Human-wt/CHN/GX54/G4P[6]** | **KF041437** | **88.8** | **88.2** | **88.6** |
|  | **T1** | **RVA/Human-tc/KOR/CAU09-376/2009/G9P[x]** | **JF766597** | **87.8** | **88.2** | **87.6** |
|  | **T1** | **RVA/Human-wt/BEL/BE2001/2009/G9P[6]** | **JQ993326** | **83.0** | **83.7** | **82.9** |
|  | **T1** | **RVA/Human/NCA/OL/2010/G4P[6]** | **JN129013** | 84.2 | 85.3 | 84.1 |
|  | T2 | RVA/Human-tc/USA/DS-1/1976/G2P1B[4] | EF672579 | 76.7 | 77.5 | 76.8 |
|  | T3 | RVA/Horse-wt/ARG/E403/2006/G14P[12] | JF712585 | 76.2 | 77.0 | 76.0 |
|  | T4 | RVA/Pigeon-tc/JPN/PO-13/1983/G18P[17] | AB009626 | 54.4 | 54.8 | 54.0 |
|  | T5 | RVA/Human-tc/KEN/B10/1987/G3P[2] | HM627561 | 74.2 | 74.3 | 74.1 |
|  | T6 | RVA/Human-tc/KEN/B12/1987/G8P[1] | HM627550 | 55.3 | 54.7 | 55.1 |
|  | T7 | RVA/Cow-tc/FRA/RF/1982/G6P[1] | Z21639 | 81.6 | 82.6 | 81.5 |
|  | T7 | RVA/Cow-tc/VEN/BRV033/1990/G6P6[1] | EF990705 | 81.9 | 82.7 | 81.8 |
|  | T8 | RVA/Chicken-tc/DEU/02V0002G3/2002/G19P[30] | FJ169859 | 80.2 | 81.3 | 80.1 |
|  | T9 | RVA/Cow-tc/JPN/Dai-10/2007/G24P[33] | AB573076 | 74.4 | 75.9 | 74.5 |
|  | T10 | RVA/Mouse-tc/USA/ETD_822/XXXX/G16P[16] | GQ479953 | 70.2 | 69.3 | 70.3 |
|  | T11 | RVA/Bat-wt/KEN/KE4852/07/2007/G25P[6] | GU983678 | 70.2 | 69.6 | 69.9 |
|  | T12 | RVA/Horse-tc/GBR/L338/1991/G13P[18] | JF712563 | 71.4 | 72.2 | 71.2 |
|  | T13 | RVA/SugarGlider-tc/JPN/SG385/2012/G27P[36] | AB971768 | 74.3 | 75.6 | 74.2 |
|  | T14 | RVA/Rat-wt/GER/KS-11-573/2011/G3P[3] | KJ879456 | 76.3 | 76.1 | 76.3 |
|  | T16 | RVA/Common_Gull-wt/JPN/Ho374/2013/G28P[39] | LC088226 | 55.1 | 53.8 | 54.8 |
| NSP4 | ***E1*** | ***RVA/Pig-tc/KOR/174-1/2006/G8P[7]*** | ***MF940573*** | **100.0** | **93.9** | **99.6** |
|  | ***E1*** | ***RVA/Pig-tc/KOR/174-1V-20/2006/G8P[7]*** | ***MF940574*** | **100.0** | **93.9** | **99.6** |
|  | ***E1*** | ***RVA/Pig-tc/KOR/174-1V-40/2006/G8P[7]*** | ***MF940575*** | **100.0** | **93.9** | **99.6** |
|  | ***E1*** | ***RVA/Pig-tc/KOR/174-1V-60/2006/G8P[7]*** | ***MF940576*** | **99.6** | **93.6** | **99.2** |
|  | ***E1*** | ***RVA/Pig-tc/KOR/174-1V-80/2006/G8P[7]*** | ***MF940577*** | **99.6** | **93.6** | **99.2** |
|  | ***E1*** | **RVA/Pig-tc/KOR/PRG942/2006/G9P[23]** | ***MF940518*** | **93.9** | **100.0** | **93.6** |
|  | ***E1*** | **RVA/Pig-tc/KOR/PRG942V-20/2006/G9P[23]** | ***MF940519*** | **93.9** | **100.0** | **93.6** |
|  | ***E1*** | **RVA/Pig-tc/KOR/PRG942V-40/2006/G9P[23]** | ***MF940520*** | **93.9** | **100.0** | **93.6** |
|  | ***E1*** | **RVA/Pig-tc/KOR/PRG942V-60/2006/G9P[23]** | ***MF940521*** | **93.9** | **100.0** | **93.6** |
|  | ***E1*** | **RVA/Pig-tc/KOR/PRG942V-80/2006/G9P[23]** | ***MF940522*** | **93.7** | **99.8** | **93.4** |
|  | ***E1*** | ***RVA/Pig-tc/KOR/K71/2006/G5P[7]*** | ***MF940463*** | **99.6** | **93.6** | **100.0** |
|  | ***E1*** | ***RVA/Pig-tc/KOR/K71V-20/2006/G5P[7]*** | ***MF940464*** | **99.6** | **93.6** | **100.0** |
|  | ***E1*** | ***RVA/Pig-tc/KOR/K71V-40/2006/G5P[7]*** | ***MF940465*** | **100.0** | **93.9** | **99.6** |
|  | ***E1*** | ***RVA/Pig-tc/KOR/K71V-60/2006/G5P[7]*** | ***MF940466*** | **99.6** | **93.6** | **99.2** |
|  | ***E1*** | ***RVA/Pig-tc/KOR/K71-80/2006/G5P[7]*** | ***MF940467*** | **99.6** | **93.6** | **99.2** |
|  | **E1** | **RVA/Pig-tc/KOR/156-1/2006/G8P[7]** | **KF500205** | **99.8** | **93.7** | **99.4** |
|  | **E1** | **RVA/Pig-tc/KOR/C-1/2006/G8P[7]** | **KF500227** | **100.0** | **93.9** | **99.6** |
|  | **E1** | **RVA/Pig-tc/KOR/PRG921/2006/G9P[23]** | **JF796710** | **93.9** | **100.0** | **93.5** |
|  | **E1** | **RVA/Pig-tc/KOR/PRG9121/2006/G9P[7]** | **JF796732** | **92.8** | **95.0** | **92.4** |
|  | **E1** | **RVA/Pig-tc/KOR/PRG9235/2006/G9P[23]** | **JF796699** | **92.8** | **95.0** | **92.4** |
|  | **E1** | **RVA/Pig-tc/USA/OSU/1977/G5P[7]** | **D88831** | **93.0** | **92.2** | **92.6** |
|  | **E1** | **RVA/Pig-tc/USA/Gottfried/1983/G4P[6]** | **GU199490** | **93.0** | **93.0** | **92.6** |
|  | **E1** | **RVA/Pig-tc/VEN/A253/1988/G11P[7]** | **AF144797** | **88.6** | **86.9** | **88.2** |
|  | **E1** | **RVA/Pig-tc/VEN/A131/1988/G3P[7]** | **AF144798** | **85.9** | **85.9** | **85.5** |
|  | **E1** | **RVA/Pig-tc/MEX/YM/1983/G11P9[7]** | **X69485** | **89.5** | **89.1** | **89.1** |
|  | **E1** | **RVA/Cow-tc/KOR/K5/2004/G5P[7]** | **JX971578** | **100.0** | **93.9** | **99.6** |
|  | **E1** | **RVA/Cow-tc/KOR/KJ25-1/2006/G8P[7]** | **FJ206109** | **100.0** | **93.9** | **99.6** |
|  | **E1** | **RVA/Cow-tc/KOR/KJ246/2006/G8P[7]** | **FJ206159** | **100.0** | **93.9** | **99.6** |
|  | **E1** | **RVA/Cow-tc/KOR/KJ330-1/2006/G8P[7]** | **FJ206162** | **100.0** | **93.9** | **99.6** |
|  | **E1** | **RVA/Cow-tc/KOR/KJ338-1/2006/G8P[7]** | **FJ206163** | **100.0** | **93.9** | **99.6** |
|  | **E1** | **RVA/Cow-tc/KOR/KJ44/2004/G5P[1]** | **DQ494397** | **99.6** | **93.9** | **99.2** |
|  | **E1** | **RVA/Cow-tc/KOR/KJ75/2004/G5P[5]** | **DQ494398** | **99.6** | **93.9** | **99.2** |
|  | **E1** | **RVA/Cow-tc/KOR/KV0407/2004/G5P[7]** | **EU873006** | **99.0** | **93.0** | **98.7** |
|  | **E1** | **RVA/Panda-tc/CHN/CH-1/2008/G1P[7]** | **GU188282** | **93.0** | **92.2** | **92.6** |
|  | **E1** | **RVA/Horse-tc/GBR/H-1/1975/G5P[7]** | **AF144800** | **91.4** | **90.5** | **91.0** |
|  | **E1** | **RVA/Human-xx/IND/mani-97/2006/G9P[19]** | **GQ240623** | **93.1** | **92.8** | **92.8** |
|  | **E1** | **RVA/Human-wt/ECU/EC2184/200x/G11P[6]** | **GQ149099** | **92.0** | **92.0** | **91.6** |
|  | **E1** | **RVA/Human-wt/BEL/Mc323/199/G9P[19]** | **JN104618** | **89.5** | **90.1** | **89.1** |
|  | **E1** | **RVA/Human-wt/BEL/BE2001/2008/G9P[6]** | **JQ993327** | **87.8** | **87.8** | **87.4** |
|  | **E1** | **RVA/human-xx/USA/DC4608-Bethesda/1980/G4P[8]** | **HM773907** | **88.0** | **87.8** | **87.6** |
|  | **E1** | **RVA/human-xx/USA/DC1208-Bethesda/1980/G4P[8]** | **HM773863** | **87.8** | **88.0** | **87.4** |
|  | **E1** | **RVA/Human-tc/USA/Wa/1974/G1P1A[8]** | **AF093199** | **87.6** | **87.8** | **87.2** |
|  | **E1** | **RVA/Human-wt/AUS/CK00005/2004/G1P[8]** | **JF490131** | **88.0** | **88.0** | **87.6** |
|  | **E1** | **RVA/Human-tc/USA/WI61/1983/G9P1A[8]** | **EF672624** | **87.4** | **87.4** | **87.0** |
|  | **E1** | **RVA/Human-wt/USA/DC104/1974/G1P[8]** | **KC579689** | **87.8** | **88.0** | **87.4** |
|  | **E1** | **RVA/Human-wt/USA/DC2314/1976/G1P[8]** | **KC579551** | **87.6** | **87.6** | **87.2** |
|  | **E1** | **RVA/Human-xx/IND/mani-253/2007/G3P[8]** | **GQ240625** | **87.0** | **88.0** | **86.7** |
|  | **E1** | **RVA/Human-wt/IND/mcs-13-07/2007/G9P[6]** | **EU753968** | **86.9** | **87.4** | **86.5** |
|  | **E1** | **RVA/Human-wt/ZAF/MRC-DPRU1262/2004/G1P[8]** | **KP752740** | **87.4** | **87.0** | **87.0** |
|  | **E1** | **RVA/Human-wt/ITA/AV21/2010/G9P[8]** | **JX195072** | **87.4** | **87.4** | **87.0** |
|  | **E1** | **RVA/Human-wt/DEU/GER126-08/2008/G12P[8]** | **FJ747623** | **87.4** | **87.8** | **87.0** |
|  | **E1** | **RVA/Human-wt/USA/DC1476/1974/G1P[8]** | **KC579562** | **87.4** | **87.4** | **87.0** |
|  | **E1** | **RVA/Human-wt/BEL/BE00043/2009/G1P[8]** | **HQ392382** | **87.2** | **87.2** | **86.9** |
|  | **E1** | **RVA/Human-wt/USA/DC1497-Bethesda/1976/G3P[8]** | **FJ947349** | **86.9** | **87.6** | **86.5** |
|  | **E1** | **RVA/Human-tc/CHN/Y128/2004/G1P[8]** | **JQ087432** | **87.0** | **86.7** | **86.7** |
|  | **E1** | **RVA/Human-wt/BEL/B3458/2003/G9P[8]** | **EF990712** | **86.9** | **86.5** | **86.5** |
|  | **E1** | **RVA/Human-wt/IND/61060/2006/G1P[8]** | **HQ609575** | **86.9** | **87.0** | **86.5** |
|  | **E1** | **RVA/Human-wt/BGD/Dhaka16/2003/G1P[8]** | **DQ492678** | **86.9** | **86.5** | **86.5** |
|  | **E1** | **RVA/Human-wt/ZWE/MRC-DPRU1723/2009/G9P[8]** | **JN605424** | **86.6** | **87.4** | **86.3** |
|  | **E1** | **RVA/Human-wt/USA/DC827/1978/G4P[8]** | **HM773962** | **86.7** | **87.4** | **86.3** |
|  | E2 | RVA/Human-tc/USA/DS-1/1976/G2P1B[4] | EF672582 | 80.2 | 80.2 | 79.8 |
|  | E3 | RVA/Dog-tc/USA/CU-1/1982/G3P[3] | EU708921 | 77.1 | 76.8 | 77.0 |
|  | E4 | RVA/Turkey-tc/IRL/Ty-1/1979/G17P[17] | AB065285 | 48.4 | 48.4 | 48.2 |
|  | E5 | RVA/Human-wt/BEL/B4106/2000/G3P[14] | AY740732 | 78.9 | 78.5 | 78.5 |
|  | E6 | RVA/Human-wt/BGD/RV176-00/2000/G12P[6] | DQ490560 | 83.6 | 82.3 | 83.4 |
|  | E7 | RVA/Mouse-tc/XXX/EHP/1981/G16P[20] | U96336 | 65.3 | 66.5 | 65.3 |
|  | E8 | RVA/Cow-lab/GBR/PP-1/1976/G3P[7] | AF427521 | 75.6 | 75.6 | 75.2 |
|  | E9 | RVA/Pig-wt/THA/CMP034/2000/G2P[27] | DQ534017 | 81.7 | 81.5 | 81.3 |
|  | E10 | RVA/Chicken-tc/DEU/02V0002G3/2002/G19P[30] | FJ169862 | 48.8 | 48.1 | 49.0 |
|  | E11 | RVA/Turkey-tc/IRL/Ty-3/1979/G7P[17] | AB065286 | 46.8 | 45.9 | 46.7 |
|  | E12 | RVA/Cow-xx/ARG/B383/1998/G15P[11] | FJ347120 | 81.0 | 80.6 | 80.6 |
|  | E13 | RVA/Human-tc/KEN/B10/1987/G3P[2] | HM627562 | 74.1 | 73.7 | 73.7 |
|  | E14 | RVA/Horse-tc/GBR/L338/1991/G13P[18] | JF712564 | 77.3 | 77.1 | 77.0 |
|  | E15 | RVA/Camel-wt/KUW/21s/2010/G10P[15] | JX968472 | 66.1 | 66.3 | 65.7 |
|  | E16 | RVA/vicugna-wt/ARG/C75/2010/G8P[14] | JX070055 | 73.0 | 73.0 | 72.8 |
|  | E17 | RVA/SugarGlider-tc/JPN/SG385/2012/G27P[36] | AB971769 | 74.7 | 73.9 | 74.5 |
|  | E18 | RVA/Rat-wt/GER/KS-11-573/2011/G3P[3] | KJ879457 | 74.1 | 73.5 | 73.9 |
|  | E21 | RVA/Common_Gull-wt/JPN/Ho374/2013/G28P[39] | LC088227 | 47.7 | 47.9 | 47.5 |
| NSP5 | ***H1*** | ***RVA/Pig-tc/KOR/174-1/2006/G8P[7]*** | ***MF940578*** | **100.0** | **97.0** | **99.8** |
|  | ***H1*** | ***RVA/Pig-tc/KOR/174-1V-20/2006/G8P[7]*** | ***MF940579*** | **100.0** | **97.0** | **99.8** |
|  | ***H1*** | ***RVA/Pig-tc/KOR/174-1V-40/2006/G8P[7]*** | ***MF940580*** | **100.0** | **97.0** | **99.8** |
|  | ***H1*** | ***RVA/Pig-tc/KOR/174-1V-60/2006/G8P[7]*** | ***MF940581*** | **100.0** | **97.0** | **99.8** |
|  | ***H1*** | ***RVA/Pig-tc/KOR/174-1V-80/2006/G8P[7]*** | ***MF940582*** | **100.0** | **97.0** | **99.8** |
|  | ***H1*** | ***RVA/Pig-tc/KOR/PRG942/2006/G9P[23]*** | ***MF940523*** | **97.0** | **100.0** | **96.8** |
|  | ***H1*** | ***RVA/Pig-tc/KOR/PRG942V-20/2006/G9P[23]*** | ***MF940524*** | **97.1** | **99.8** | **97.0** |
|  | ***H1*** | ***RVA/Pig-tc/KOR/PRG942V-40/2006/G9P[23]*** | ***MF940525*** | **97.1** | **99.2** | **97.0** |
|  | ***H1*** | ***RVA/Pig-tc/KOR/PRG942V-60/2006/G9P[23]*** | ***MF940526*** | **97.1** | **99.5** | **97.0** |
|  | ***H1*** | ***RVA/Pig-tc/KOR/PRG942V-80/2006/G9P[23]*** | ***MF940527*** | **97.1** | **99.5** | **97.0** |
|  | ***H1*** | ***RVA/Pig-tc/KOR/K71/2006/G5P[7]*** | ***MF940468*** | **99.8** | **96.8** | **100.0** |
|  | ***H1*** | ***RVA/Pig-tc/KOR/K71V-20/2006/G5P[7]*** | ***MF940469*** | **100.0** | **97.0** | **99.8** |
|  | ***H1*** | ***RVA/Pig-tc/KOR/K71V-40/2006/G5P[7]*** | ***MF940470*** | **100.0** | **97.0** | **99.8** |
|  | ***H1*** | ***RVA/Pig-tc/KOR/K71V-60/2006/G5P[7]*** | ***MF940471*** | **99.7** | **96.6** | **99.5** |
|  | ***H1*** | ***RVA/Pig-tc/KOR/K71-80/2006/G5P[7]*** | ***MF940472*** | **99.5** | **96.4** | **99.3** |
|  | **H1** | **RVA/Pig-tc/KOR/156-1/2006/G8P[7]** | **KF500206** | **97.6** | **97.0** | **97.5** |
|  | **H1** | **RVA/Pig-tc/KOR/C-1/2006/G8P[7]** | **KF500228** | **99.8** | **96.8** | **99.7** |
|  | **H1** | **RVA/Pig-tc/MEX/YM/1983/G11P[7]** | **X69486** | **97.8** | **97.6** | **97.6** |
|  | **H1** | **RVA/Pig-tc/KOR/PRG9121/2006/G9P[7]** | **JF796733** | **98.0** | **97.6** | **97.8** |
|  | **H1** | **RVA/Pig-tc/USA/Gottfried/1983/G4P[6]** | **GU199491** | **97.8** | **97.1** | **97.6** |
|  | **H1** | **RVA/Pig-tc/KOR/PRG921/2006/G9P[23]** | **JF796711** | **97.3** | **99.7** | **97.1** |
|  | **H1** | **RVA/Pig-wt/IND/RU172/2002/G12P[7]** | **DQ204739** | **97.3** | **97.0** | **97.1** |
|  | **H1** | **RVA/Pig-tc/USA/OSU/1977/G5P[7]** | **X15519** | **99.0** | **96.4** | **98.8** |
|  | **H1** | **RVA/Pig-tc/KOR/PRG9235/2006/G9P[23]** | **JF796700** | **97.1** | **99.5** | **97.0** |
|  | **H1** | **RVA/Pig-tc/VEN/A253/1988/G11P[7]** | **EF990698** | **94.1** | **91.7** | **93.9** |
|  | **H1** | **RVA/Pig-tc/VEN/A131/1988/G3P[7]** | **EF990690** | **92.2** | **90.2** | **92.0** |
|  | **H1** | **RVA/Cow-tc/KOR/K5/2004/G5P[7]** | **JX971579** | **100.0** | **97.0** | **99.8** |
|  | **H1** | **RVA/Cow-tc/KOR/KJ25-1/2006/G8P[7]** | **FJ206059** | **100.0** | **97.0** | **99.8** |
|  | **H1** | **RVA/Cow-tc/KOR/KJ246/2006/G8P[7]** | **FJ206097** | **100.0** | **97.0** | **99.8** |
|  | **H1** | **RVA/Cow-tc/KOR/KJ330-1/2006/G8P[7]** | **FJ206099** | **100.0** | **97.0** | **99.8** |
|  | **H1** | **RVA/Cow-tc/KOR/KJ338-1/2006/G8P[7]** | **FJ206100** | **100.0** | **97.0** | **99.8** |
|  | **H1** | **RVA/Cow-tc/KOR/KJ44/2004/G5P[1]** | **DQ494399** | **100.0** | **97.0** | **99.8** |
|  | **H1** | **RVA/Cow-tc/KOR/KJ75/2004/G5P[5]** | **DQ494400** | **100.0** | **97.0** | **99.8** |
|  | **H1** | **RVA/Cow-tc/KOR/K8/2005/G5P[7]** | **EU542727** | **100.0** | **97.0** | **99.8** |
|  | **H1** | **RVA/Cow-tc/KOR/KJ9-1/2006/G6P[7]** | **FJ206045** | **99.5** | **96.4** | **99.3** |
|  | **H1** | **RVA/Horse-tc/GBR/H-1/1975/G5P[7]** | **JQ309144** | **97.5** | **97.1** | **97.3** |
|  | **H1** | **RVA/Panda-tc/CHN/CH-1/2008/G1P[7]** | **GU329526** | **99.5** | **96.8** | **99.3** |
|  | **H1** | **RVA/Human-wt/HUN/BP271/2000/G4P[6]** | **KF835969** | **98.3** | **98.0** | **98.1** |
|  | **H1** | **RVA/Human-wt/JPN/Ryukyu-1120/2011/G5P[6]** | **AB741659** | **98.1** | **97.6** | **98.0** |
|  | **H1** | **RVA/Human-wt/BEL/BE00055/1999/G1P[8]** | **JN258796** | **97.3** | **97.0** | **97.1** |
|  | **H1** | **RVA/Human-xx/IND/mani-362/2007/G4P[6]** | **HM348731** | **97.5** | **97.1** | **97.3** |
|  | **H1** | **RVA/Human-wt/USA/DC1117/1977/G1P[8]** | **KC580345** | **97.6** | **97.0** | **97.5** |
|  | **H1** | **RVA/Human-wt/USA/DC827/1978/G4P[8]** | **HM773963** | **97.3** | **97.0** | **97.1** |
|  | **H1** | **RVA/Human-wt/DEU/GER126-08/2008/G12P[8]** | **FJ747624** | **97.0** | **97.0** | **96.8** |
|  | **H1** | **RVA/Human-tc/CHN/Y128/2004/G1P[8]** | **JQ087433** | **96.8** | **96.4** | **96.6** |
|  | **H1** | **RVA/Human-wt/BEL/BE2001/2008/G9P[6]** | **JQ993328** | **96.8** | **97.0** | **96.6** |
|  | **H1** | **RVA/Human-tc/USA/WI61/1983/G9P1A[8]** | **EF672625** | **97.3** | **96.6** | **97.1** |
|  | **H1** | **RVA/Human-wt/ECU/EC2184/200x/G11P[6]** | **GQ149100** | **96.8** | **96.1** | **96.6** |
|  | **H1** | **RVA/Human-wt/BEL/BE00038/2008/G1P[8]** | **HQ392335** | **96.4** | **96.1** | **96.3** |
|  | **H1** | **RVA/Human-wt/BEL/B3458/2003/G9P[8]** | **EF990713** | **96.3** | **95.9** | **96.1** |
|  | **H1** | **RVA/Human-wt/IND/61060/2006/G1P[8]** | **HQ609578** | **96.6** | **96.3** | **96.4** |
|  | **H1** | **RVA/Human-wt/BEL/B3458/2003/G9P[8]** | **EF990713** | **96.3** | **95.9** | **96.1** |
|  | **H1** | **RVA/Human-wt/BGD/Dhaka16/2003/G1P[8]** | **DQ492679** | **95.9** | **95.6** | **95.8** |
|  | **H1** | **RVA/Human-wt/ZAE/MRC-DPRU1723/2009/G9P[8]** | **JN605425** | **96.1** | **95.8** | **95.9** |
|  | **H1** | **RVA/Human-xx/IND/mcs-10/2007/G9P[6]** | **EU753974** | **95.4** | **95.1** | **95.3** |
|  | **H1** | **RVA/Human-xx/IND/mani-97/2006/G9P[19]** | **HM348728** | **95.1** | **95.9** | **94.9** |
|  | **H1** | **RVA/Human-xx/IND/mani-253/2007/G3P[8]** | **HM348729** | **94.9** | **95.1** | **94.8** |
|  | **H1** | **RVA/Human-wt/USA/DC4320/1988/G4P[8]** | **HM773897** | **93.9** | **93.4** | **93.7** |
|  | **H1** | **RVA/Human-wt/USA/DC4608-Bethesda/1980/G4P[8]** | **HM773908** | **93.9** | **93.4** | **93.7** |
|  | **H1** | **RVA/Human-wt/USA/DC1476/1974/G1P[8]** | **KC579563** | **94.1** | **93.6** | **93.9** |
|  | **H1** | **RVA/Human-wt/USA/DC2106-Bethesda/1976/G3P[8]** | **FJ947847** | **94.1** | **93.6** | **93.9** |
|  | **H1** | **RVA/Human-wt/USA/DC2314/1976/G1P[8]** | **KC579552** | **93.9** | **93.4** | **93.7** |
|  | **H1** | **RVA/Human-wt/USA/DC102/1974/G1P[8]** | **KC580004** | **93.9** | **93.4** | **93.7** |
|  | **H1** | **RVA/Human-tc/USA/Wa/1974/G1P1A[8]** | **AF306494** | **93.9** | **93.4** | **93.7** |
|  | **H1** | **RVA/Human-wt/AUS/CK00005/2004/G1P[8]** | **JF490130** | **93.6** | **93.1** | **93.4** |
|  | H2 | RVA/Human-tc/USA/DS-1/1976/G2P1B[4] | EF672583 | 83.1 | 83.6 | 82.9 |
|  | H3 | RVA/Human-tc/KEN/B12/1987/G8P[1] | HM627552 | 88.5 | 88.2 | 88.3 |
|  | H4 | RVA/Pigeon-tc/JPN/PO-13/1983/G18P[17] | AB009628 | 62.3 | 62.2 | 62.2 |
|  | H5 | RVA/Human-tc/KEN/B10/1987/G3P[2] | HM627563 | 87.5 | 86.8 | 87.3 |
|  | H6 | RVA/Dog-tc/USA/CU-1/1982/G3P[3] | EU708922 | 89.0 | 88.7 | 88.8 |
|  | H7 | RVA/Horse-wt/ARG/E403/2006/G14P[12] | JF712587 | 82.5 | 82.6 | 82.3 |
|  | H8 | RVA/Chicken-tc/DEU/02V0002G3/2002/G19P[30] | FJ169863 | 58.6 | 59.0 | 58.6 |
|  | H9 | RVA/Mouse-tc/USA/ETD_822/XXXX/G16P[16] | GQ479957 | 78.6 | 78.8 | 78.5 |
|  | H10 | RVA/Bat-wt/KEN/KE4852/07/2007/G25P[6] | GU983680 | 83.9 | 83.2 | 83.8 |
|  | H11 | RVA/Horse-tc/GBR/L338/1991/G13P[18] | JF712565 | 81.7 | 82.4 | 81.6 |
|  | H12 | RVA/SugarGlider-tc/JPN/SG385/2012/G27P[36] | AB971770 | 83.4 | 82.1 | 83.2 |
|  | H13 | RVA/Rat-wt/GER/KS-11-573/2011/G3P[3] | KJ879458 | 84.6 | 83.6 | 84.4 |
|  | H14 | RVA/Turkey-tc/IRL/Ty-3/1979/G7P[17] | LC088124 | 61.9 | 60.9 | 61.7 |
|  | H16 | RVA/Common_Gull-wt/JPN/Ho374/2013/G28P[39] | LC088228 | 60.6 | 60.2 | 60.4 |

^a^ ORF: open reading frame.

^b^ Bold letters indicate the genotype of the strains used in this experiment and other strains belonging to the same genotype.

^c^ The cut-off values of nucleotide percentage identities of each gene segment is as follows: VP7: 80%, VP4: 80%, VP6: 85%, VP1: 83%, VP2: 84%,VP3: 81%, NSP1: 79%, NSP2: 85%, NSP3: 85%, NSP4: 85%, and NSP5: 91%.

^d^ Nucleotide sequences are not yet available in GenBank.
