# Supplementary material for: Development of a live attenuated trivalent porcine rotavirus A vaccine against disease caused by recent strains most prevalent in South Korea
Source: Vet Res. 2019 Jan 7;50:2. doi: 10.1186/s13567-018-0619-6 (PMC6323864; doi:10.1186/s13567-018-0619-6)
Supplement: Supplementary file 4 — Additional file 4. Summary of the histopathological findings in the small intestine of the colostrums-deprived neonatal piglets inoculated with each virulent strains (174-1, PRG942, and K71), or immunized with each live attenuated monovalent or trivalent vaccines and then challenged with each corresponding original virulent strain(s). [file 13567_2018_619_MOESM4_ESM.docx]

**Additional file 4** **Summary of the histopathological findings in the small intestine of the colostrums-deprived neonatal piglets inoculated with each virulent strains (174-1, PRG942, and K71), or immunized with each live attenuated monovalent or trivalent vaccines and then challenged with its each corresponding original virulent strain(s).**

| Virulent or attenuated strains | Piglet  No. | dpi^a^ at euthanasia | Duodenum | Jejunum | Ileum |
| --- | --- | --- | --- | --- | --- |
|  |  |  | Lesion score^b^ | Lesion score^b^ | Lesion score^b^ |
| Virulent 174-1 | 1 | 7 | 3.4 | 3.4 | 3.6 |
|  | 2 | 7 | 3.6 | 3.8 | 3.8 |
|  | 3 | 7 | 3.4 | 3.4 | 3.8 |
| Virulent PRG942 | 4 | 7 | 3.2 | 3.6 | 3.6 |
|  | 5 | 7 | 3.4 | 3.8 | 3.8 |
|  | 6 | 7 | 3.4 | 3.8 | 3.4 |
| Virulent K71 | 7 | 7 | 3.6 | 3.8 | 3.8 |
|  | 8 | 7 | 3.8 | 3.4 | 4.0 |
|  | 9 | 7 | 3.8 | 3.4 | 4.0 |
| 174-1V-80 vaccination followed by 174-1 challenge | 10 | 28 | 1.4 | 1.6 | 1.6 |
|  | 11 | 28 | 1.0 | 1.2 | 1.4 |
|  | 12 | 28 | 1.4 | 1.2 | 1.6 |
|  | 13 | 28 | 1.2 | 1.6 | 1.8 |
|  | 14 | 28 | 1.6 | 1.4 | 1.4 |
| PRG942V-80 vaccination followed by PRG942 challenge | 15 | 28 | 1.0 | 1.4 | 1.2 |
|  | 16 | 28 | 1.2 | 1.6 | 1.6 |
|  | 17 | 28 | 1.4 | 1.6 | 1.2 |
|  | 18 | 28 | 1.4 | 1.6 | 1.8 |
|  | 19 | 28 | 1.6 | 1.2 | 1.6 |
| K71V-80 vaccination followed by K71 challenge | 20 | 28 | 1.2 | 1.6 | 1.6 |
|  | 21 | 28 | 1.0 | 1.2 | 1.8 |
|  | 22 | 28 | 1.4 | 1.2 | 1.2 |
|  | 23 | 28 | 1.6 | 1.8 | 1.4 |
|  | 24 | 28 | 2.0 | 1.6 | 1.8 |
| Trivalent vaccination followed by challenge with a mixture of virulent 174-1, PRG942, and K71 | 25 | 28 | 1.8 | 1.8 | 1.6 |
|  | 26 | 28 | 1.4 | 1.8 | 2.0 |
|  | 27 | 28 | 1.6 | 1.6 | 1.2 |
|  | 28 | 28 | 1.2 | 1.4 | 1.6 |
|  | 29 | 28 | 1.6 | 1.6 | 1.4 |
| Mock-inoculated^c^ | 30 | 28 | 0.4 | 0.2 | 0.2 |
|  | 31 | 28 | 0.2 | 0.2 | 0.2 |
|  | 32 | 28 | 0.2 | 0.2 | 0.4 |
|  | 33 | 28 | 0.4 | 0.2 | 0.4 |
|  | 34 | 28 | 0.2 | 0.2 | 0.4 |

^a^ dpi: days post-inoculation.

^b^ The small intestinal changes were scored according to the average villi/crypt (V/C) ratio plus the grade of epithelial cell desquamation, which was measured as follows: V/C ratio, 0 = normal (V/C≧6:1), 1 = mild (V/C = 5.0 to 5.9:1), 2 = moderate (V/C = 4.0 to 4.9:1), 3 = marked (V/C = 3.0 to 3.9:1), 4 = severe (V/C ≦3.0:1) and desquamation grade, 0 = normal (no desquamation), 1 = mild (cuboidal attenuation of tip villous epithelium), 2 = moderate (desquamation of upper villous epithelium), 3 = marked (desquamation of lower villous epithelium), 4 = severe (desquamation of crypt epithelium).

^c^ Inoculated with serum-free α-MEM.
