# Supplementary material for: Development of a live attenuated trivalent porcine rotavirus A vaccine against disease caused by recent strains most prevalent in South Korea
Source: Vet Res. 2019 Jan 7;50:2. doi: 10.1186/s13567-018-0619-6 (PMC6323864; doi:10.1186/s13567-018-0619-6)
Supplement: Supplementary file 5 — Additional file 5. Summary of safety test results for a live attenuated porcine rotavirus monovalent vaccine strains 174-1V-80, PRG942V-80, and K71V-80 in mice, guinea pigs, and pigs. [file 13567_2018_619_MOESM5_ESM.docx]

**Additional file 5** **Summary of safety test results for a live attenuated porcine rotavirus monovalent vaccine strains 174-1V-80, PRG942V-80, and K71V-80 in mice, guinea pigs, and pigs.**

| Species | Strain | Route of administration | Age of animals | No. of animals | Amount of vaccine  (1.0 × 10^6^ ffu/mL) | Clinical signs |
| --- | --- | --- | --- | --- | --- | --- |
| Mice | 174-1V-80 | Intra-peritoneum | 7 weeks | 24 | 0.5 mL | None |
|  | PRG942V-80 | Intra-peritoneum | 7 weeks | 24 | 0.5 mL | None |
|  | K71V-80 | Intra-peritoneum | 7 weeks | 24 | 0.5 mL | None |
| Guinea pigs | 174-1V-80 | Intra-muscle | 7 weeks | 6 | 2 mL | None |
|  | PRG942V-80 | Intra-muscle | 7 weeks | 6 | 2 mL | None |
|  | K71V-80 | Intra-muscle | 7 weeks | 6 | 2 mL | None |
|  | 174-1V-80 | Intra-peritoneum | 7 weeks | 6 | 2 mL | None |
|  | PRG942V-80 | Intra-peritoneum | 7 weeks | 6 | 2 mL | None |
|  | K71V-80 | Intra-peritoneum | 7 weeks | 6 | 2 mL | None |
| Pigs | 174-1V-80 | Intra-muscle | 4 weeks | 6 | 10 mL | None |
|  | PRG942V-80 | Intra-muscle | 4 weeks | 6 | 10 mL | None |
|  | K71V-80 | Intra-muscle | 4 weeks | 6 | 10 mL | None |
