# Supplementary material for: Development of a live attenuated trivalent porcine rotavirus A vaccine against disease caused by recent strains most prevalent in South Korea
Source: Vet Res. 2019 Jan 7;50:2. doi: 10.1186/s13567-018-0619-6 (PMC6323864; doi:10.1186/s13567-018-0619-6)
Supplement: Supplementary file 6 — Additional file 6. Virus titers of each strain in different passages. [file 13567_2018_619_MOESM6_ESM.docx]

**Additional file 6 Virus titers of each strain in different passages**

| **Strains** | **G and P genotypes** | **Passage number** | | | | | | | | | | | | | | |
| --- | --- | --- | --- | --- | --- | --- | --- | --- | --- | --- | --- | --- | --- | --- | --- | --- |
|  |  | **1^st^** | | | **20^th^** | | | **40^th^** | | | **60^th^** | | | **80^th^** | | |
|  |  | CCIF^a^ | qRT-PCR^b^ | Ratio of B/A^c^ | CCIF | qRT-PCR | Ratio of B/A | CCIF | qRT-PCR | Ratio of B/A | CCIF | qRT-PCR | Ratio of B/A | CCIF | qRT-PCR | Ratio of B/A |
| 174-1 | G8P[7] | 6.77 | 7.81 | 1.15 | 10.81 | 11.86 | 1.10 | 11.89 | 13.07 | 1.10 | 12.14 | 14.39 | 1.18 | 13.17 | 15.55 | 1.18 |
| PRG942 | G9P[23] | 6.49 | 7.51 | 1.16 | 9.51 | 10.71 | 1.13 | 11.14 | 12.32 | 1.11 | 11.92 | 12.93 | 1.08 | 12.74 | 13.14 | 1.03 |
| K71 | G5P[7] | 9.55 | 10.07 | 1.05 | 10.94 | 11.04 | 1.01 | 12.99 | 13.56 | 1.04 | 13.06 | 14.62 | 1.12 | 13.39 | 15.89 | 1.19 |

^a^ CCIF: Cell culture immunofluorescence and calculated as fluorescence focus unit (FFU)/mL (Log_10_).

^b^ qRT-PCR: Real-time reverse transcriptase polymerase chain reaction calculated as genome copy numbers/mL (Log_10)_.

^c^ Ratio of genome copy number/infectivity.
