# Supplementary figures and images for: Development of a live attenuated trivalent porcine rotavirus A vaccine against disease caused by recent strains most prevalent in South Korea
Source: Vet Res. 2019 Jan 7;50:2. doi: 10.1186/s13567-018-0619-6 (PMC6323864; doi:10.1186/s13567-018-0619-6)

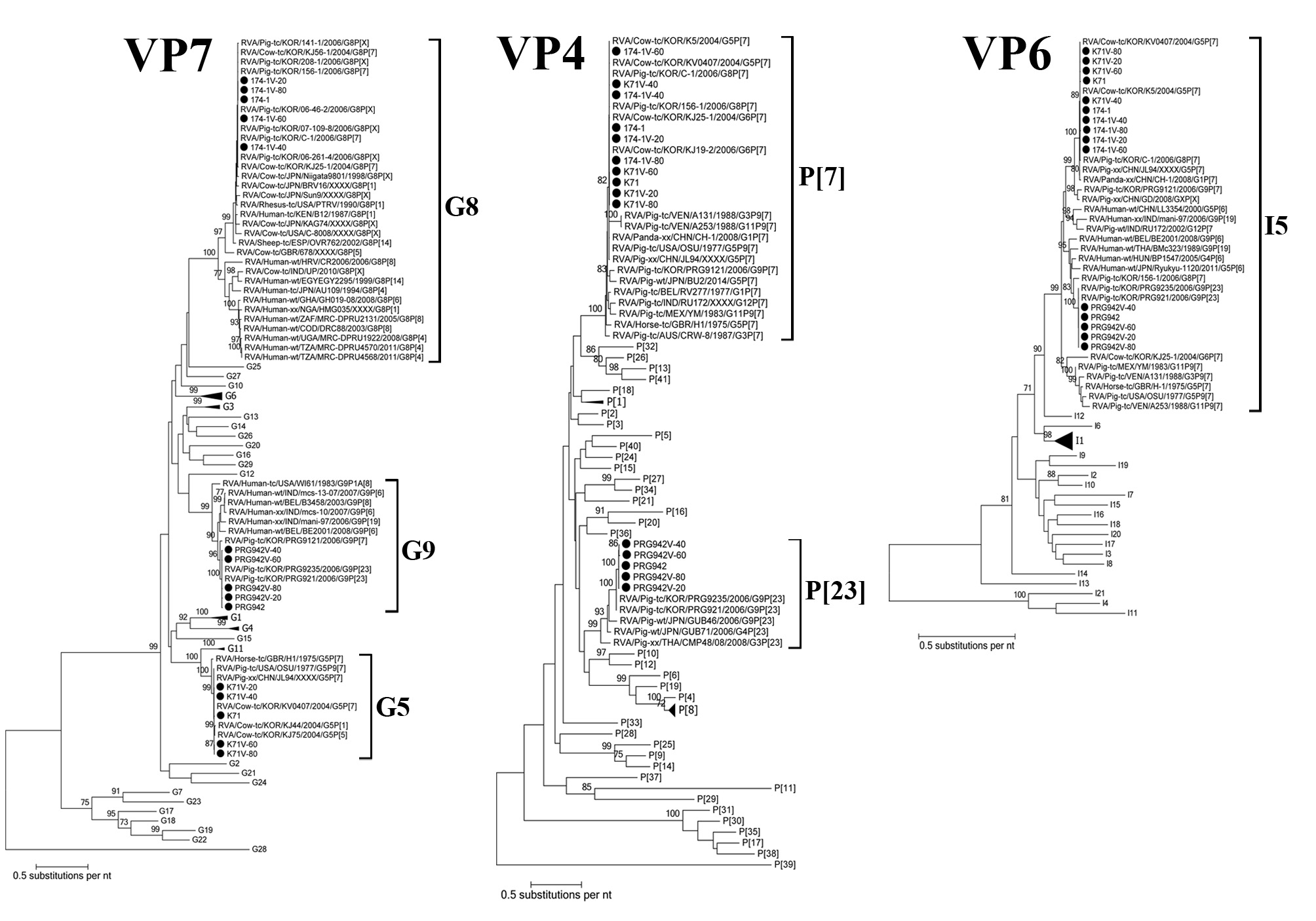

Supplement: Supplementary file 7 — Additional file 7. Phylogenetic trees based on full-length ORF nucleotide sequences of the VP7, VP4, and VP6 gene segments of RVA strains 174-1, K71, and PRG942. Phylogenetic trees were constructed using the maximum likelihood method based on General Time Reversible (GTR) with gamma distributed substitution model with 500 bootstrap replicates by MEGA 6 software [26]. The GenBank accession numbers for each of the reference genes are listed in Additional file 3. The following data are provided to explain each strain: Serotype of rotavirus/species of origin-virus type/country/strain name/isolation year/G and P genotype is indicated. The serial passage of the porcine vaccine strains is represented by closed circles. [file 13567_2018_619_MOESM7_ESM.jpg]

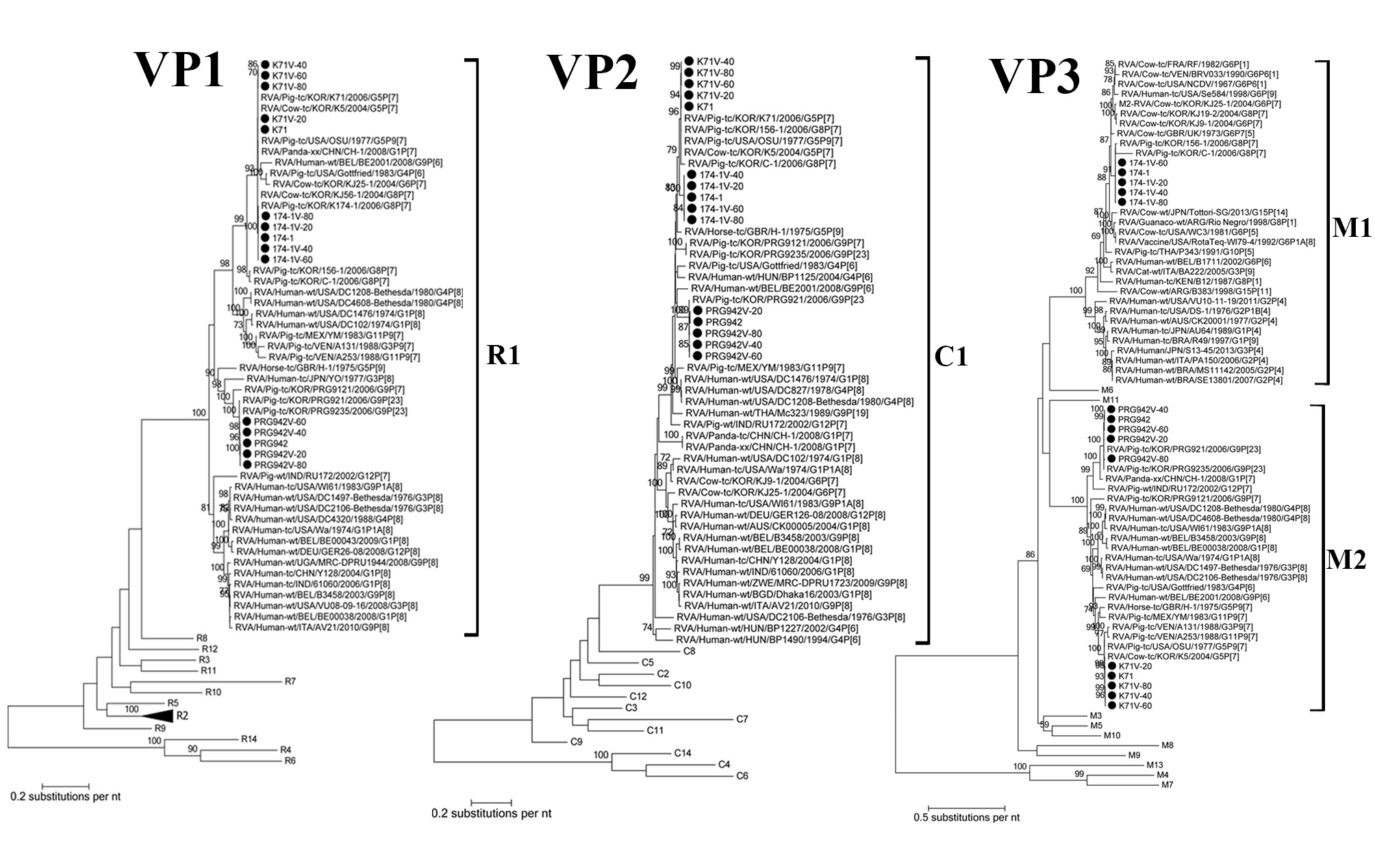

Supplement: Supplementary file 8 — Additional file 8. Phylogenetic trees based on full-length ORF nucleotide sequences of the VP1, VP2, and VP3 gene segments of RVA strains 174-1, K71, and PRG942. Phylogenetic trees were constructed using the maximum likelihood method based on General Time Reversible (GTR) with gamma distributed substitution model with 500 bootstrap replicates by MEGA 6 software [26]. The GenBank accession numbers for each of the reference genes are listed in Additional file 3. The following data are provided to explain each strain: Serotype of rotavirus/species of origin-virus type/country/strain name/isolation year/G- and P-genotype is indicated. The serial passage of the porcine vaccine strains is represented by closed circles. [file 13567_2018_619_MOESM8_ESM.jpg]

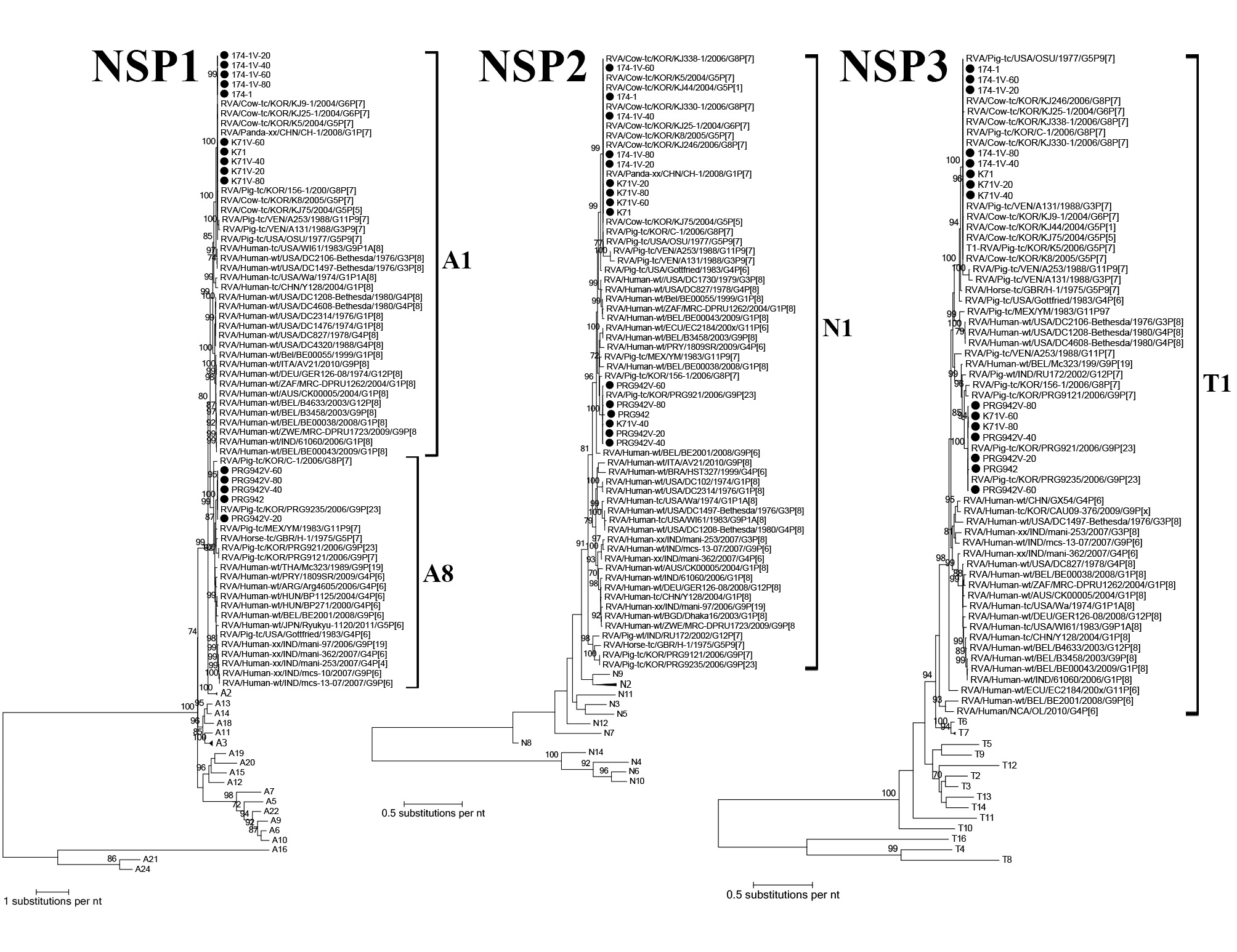

Supplement: Supplementary file 9 — Additional file 9. Phylogenetic trees based on full-length ORF nucleotide sequences of the NSP1, NSP2 and NSP3 gene segments of RVA strains 174-1, K71, and PRG942. Phylogenetic trees were constructed using the maximum likelihood method based on General Time Reversible (GTR) with gamma distributed substitution model with 500 bootstrap replicates by MEGA 6 software [26]. The GenBank accession number for each of the reference genes are listed in Additional file 3. The following data are provided to explain each strain: Serotype of rotavirus/species of origin-virus type/country/strain name/isolation year/G- and P-genotype is indicated. The serial passage of the porcine vaccine strains is represented by closed circles. [file 13567_2018_619_MOESM9_ESM.jpg]

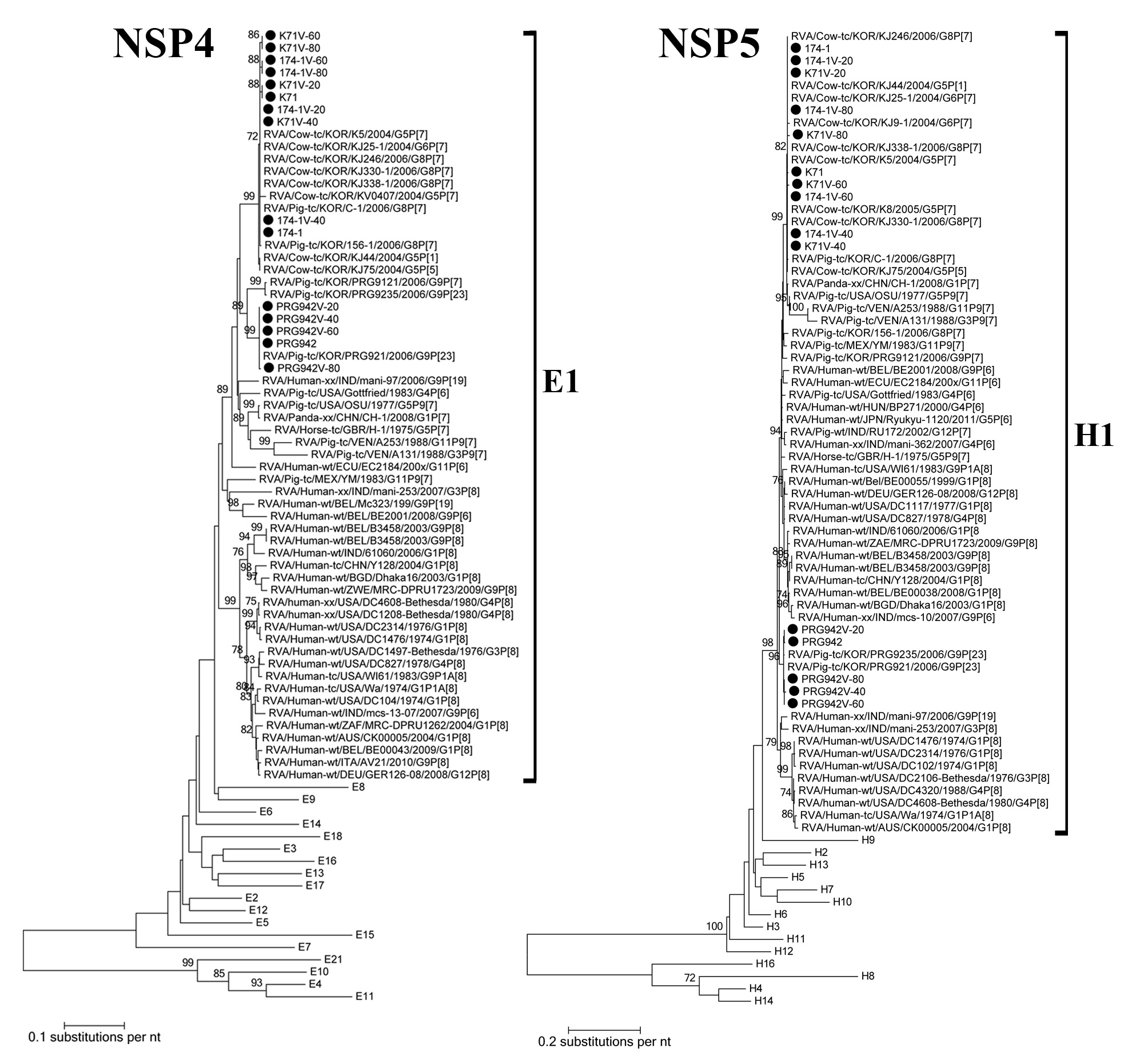

Supplement: Supplementary file 10 — Additional file 10. Phylogenetic trees based on full-length ORF nucleotide sequences of the NSP4 and NSP5 gene segments of RVAA strains 174-1, K71, and PRG942. Phylogenetic trees were constructed using the neighbor-joining method based on Kimura-2 (NSP4) or maximum likelihood method based General Time Reversible (GTR) (NSP5) with gamma distributed substitution model with 500 bootstrap replicates by MEGA 6 software [26]. The GenBank accession number for each of the reference genes are listed in Additional file 3. The following data are provided to explain each strain: Serotype of rotavirus/species of origin-virus type/country/strain name/isolation year/G- and P-genotype is indicated. The serial passage of the porcine vaccine strains is represented by closed circles. [file 13567_2018_619_MOESM10_ESM.jpg]

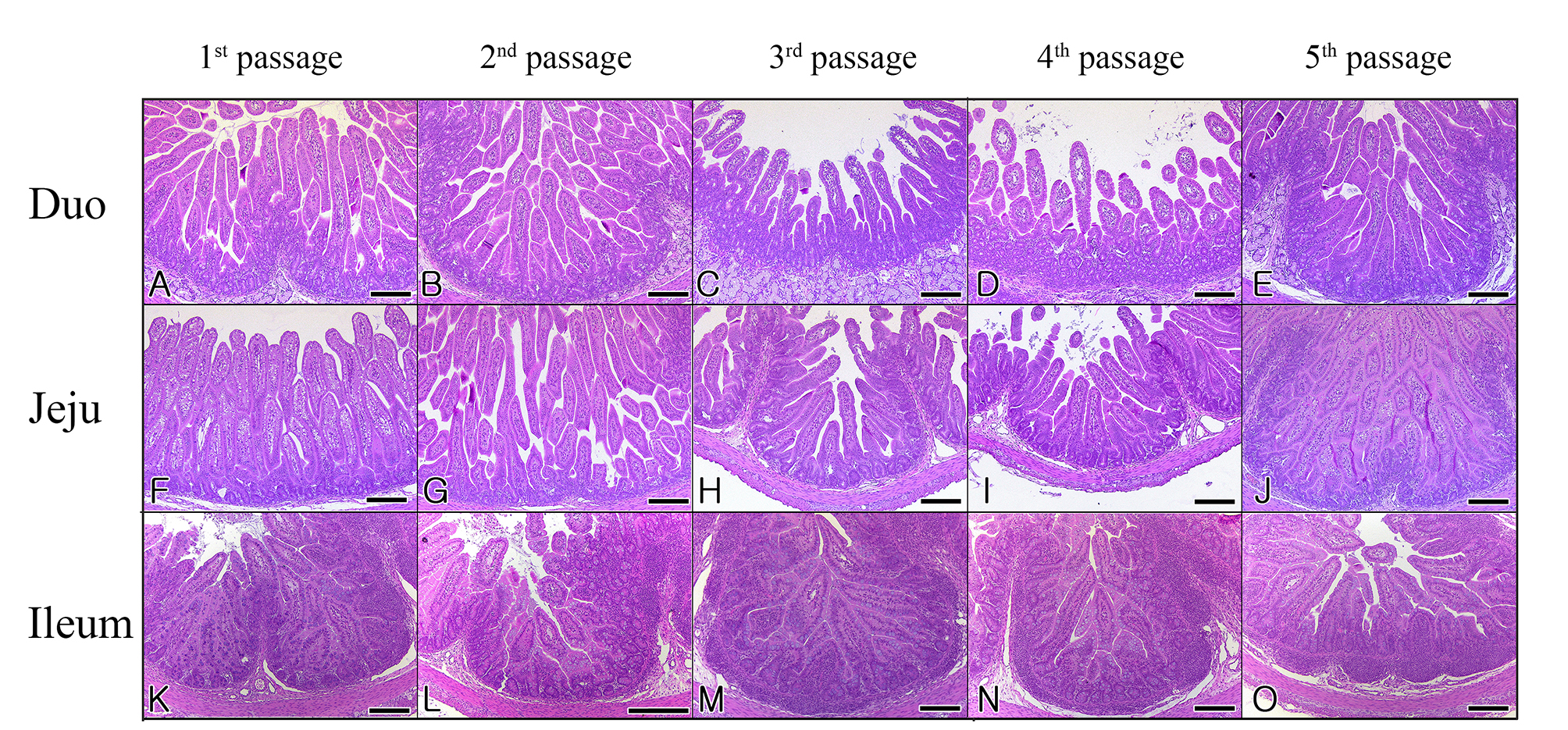

Supplement: Supplementary file 17 — Additional file 17. Histopathological changes in the small intestines of piglets inoculated with a porcine live attenuated monovalent rotavirus strain (174-1V-80) and its serial passages. (A–E) Duodenum sampled from piglets inoculated with each of the serial passaged viruses demonstrated normal long slender villi and short crypts in the mucosal membrane. (F–J) Jejunum sampled from piglets inoculated with each of the serial passaged viruses demonstrated normal long slender villi and short crypts in the mucosal membrane. (K–O) Ileum sampled from piglets inoculated with each of the serial passaged viruses demonstrated normal long slender villi and short crypts in the mucosal membrane. Bar = 200 μm. [file 13567_2018_619_MOESM17_ESM.jpg]

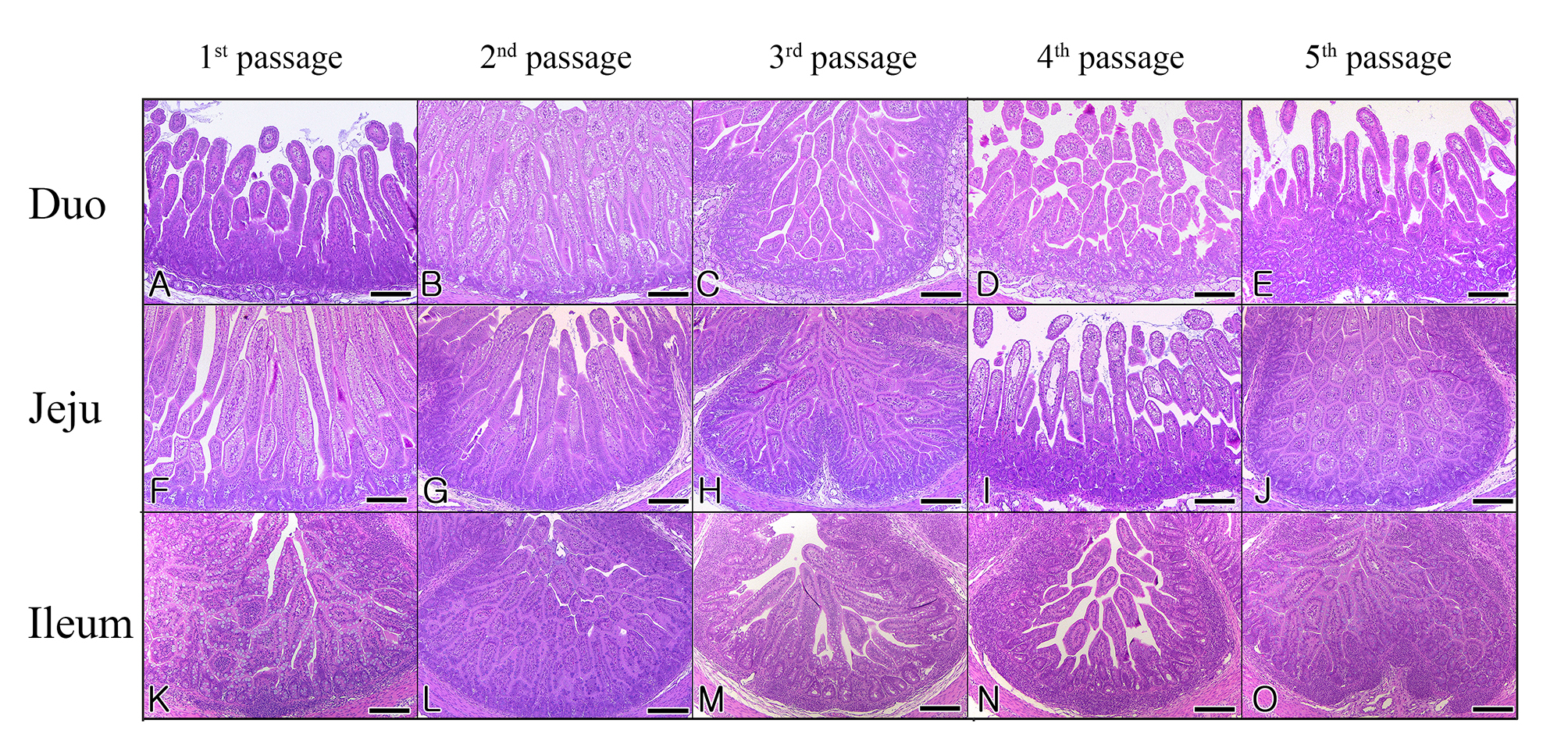

Supplement: Supplementary file 18 — Additional file 18. Histopathological changes in the small intestine of piglets inoculated with a porcine live attenuated monovalent rotavirus strain (PRG942V-80) and its serial passages. (A–E) Duodenum sampled from piglets inoculated with each of the serial passaged viruses demonstrated normal long slender villi and short crypts in the mucosal membrane. (F–J) Jejunum sampled from piglets inoculated with each of the serial passaged viruses demonstrated normal long slender villi and short crypts in the mucosal membrane. (K–O) Ileum sampled from piglets inoculated with each of the serial passaged viruses demonstrated normal long slender villi and short crypts in the mucosal membrane. Bar = 200 μm. [file 13567_2018_619_MOESM18_ESM.jpg]

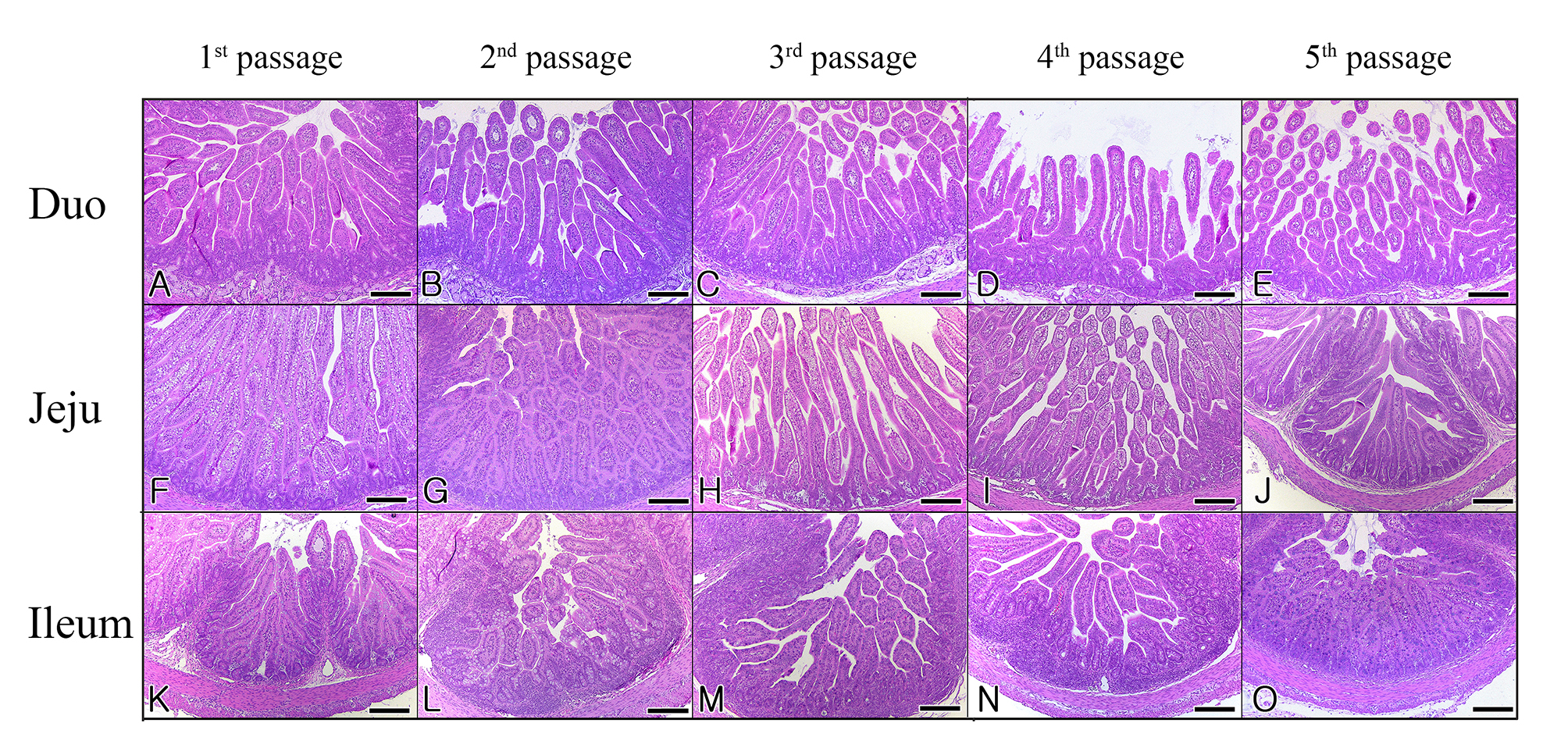

Supplement: Supplementary file 19 — Additional file 19. Histopathological changes in the small intestine of piglets inoculated with a porcine live attenuated monovalent rotavirus strain (K71V-80) and its serial passages. (A–E) Duodenum sampled from piglets inoculated with each of the serial passaged viruses demonstrated normal long slender villi and short crypts in the mucosal membrane. (F–J) Jejunum sampled from piglets inoculated with each of the serial passaged viruses demonstrated normal long slender villi and short crypts in the mucosal membrane. (K–O) Ileum sampled from piglets inoculated with each of the serial passaged viruses demonstrated normal long slender villi and short crypts in the mucosal membrane. Bar = 200 μm. [file 13567_2018_619_MOESM19_ESM.jpg]
