# Supplementary material for: Development of a live attenuated trivalent porcine rotavirus A vaccine against disease caused by recent strains most prevalent in South Korea
Source: Vet Res. 2019 Jan 7;50:2. doi: 10.1186/s13567-018-0619-6 (PMC6323864; doi:10.1186/s13567-018-0619-6)
Supplement: Supplementary file 11 — Additional file 11. Comparison of full-length amino acid sequences of 11 genomic segments of 174-1V-80 (G8P[7]) vaccine strain with its different passages. The full-length amino acid sequences of the 11 genomic segments of the 80th-passage attenuated 174-1V-80 vaccine strain was compared with those of the 20th-, 40th-, and 60th-passage attenuated strains and the original virulent strain. [file 13567_2018_619_MOESM11_ESM.docx]

**Additional file 11 Comparison of full-length amino acid sequences of 11 genomic segments of 174-1V-80 (G8P[7]) vaccine strain with its different passages**

| Gene segment | | | Amino acid position | Passage No. | | | | |
| --- | --- | --- | --- | --- | --- | --- | --- | --- |
|  |  |  |  | 174-1  (1^st^) | 174-1V-20  (20^th^) | 174-1V-40  (40^th^) | 174-1V-60  (60^th^) | 174-1V-80  (80^th^) |
|  | | VP1 | 328 | W | W | W | W | **A** |
|  |  | | 630 | G | G | G | G | **T** |
|  |  |  | 820 | L | L | L | L | **N** |
|  |  |  | 986 | Y | Y | Y | Y | **W** |
|  | VP2 | | 43 | Q | Q | Q | Q | **P** |
|  |  |  | 98 | I | I | I | **L** | **L** |
|  |  |  | 470 | E | E | E | **K** | **K** |
|  | VP3 | | 19 | I | **R** | I | I | I |
|  |  |  | 153 | X | **A** | **A** | **A** | **A** |
|  |  |  | 279 | K | K | **N** | K | **N** |
|  |  |  | 300 | P | P | P | **S** | P |
|  | VP4 | | 123 | G | G | G | G | **R** |
|  |  |  | 323 | V | V | V | **D** | V |
|  |  |  | 711 | L | L | L | L | **I** |
|  | VP6 | | 137 | E | E | E | E | **K** |
|  |  |  | 201 | A | A | A | A | **G** |
|  |  |  | 325 | R | R | R | R | **P** |
|  | VP7 | | 73 | R | R | **P** | **P** | R |
|  | NSP1 | | 61 | H | H | H | **W** | **W** |
|  |  |  | 240 | S | S | S | **T** | **T** |
|  |  |  | 301 | L | L | L | **I** | **I** |
|  |  |  | 360 | G | G | G | **I** | **I** |
|  | NSP2 | | 178 | A | **P** | **P** | **P** | **P** |
|  | NSP3 | | 88 | M | M | M | M | **X** |
|  | NSP4 | | 83 | A | A | A | **T** | **T** |
|  |  |  | 114 | D | D | D | **G** | **G** |
