# Supplementary material for: Development of a live attenuated trivalent porcine rotavirus A vaccine against disease caused by recent strains most prevalent in South Korea
Source: Vet Res. 2019 Jan 7;50:2. doi: 10.1186/s13567-018-0619-6 (PMC6323864; doi:10.1186/s13567-018-0619-6)
Supplement: Supplementary file 12 — Additional file 12. Comparison of full-length amino acid sequences of 11 genomic segments of PRG942V-80 (G9P[23]) vaccine strain with its different passages. The full-length amino acid sequences of the 11 genomic segments of the 80th-passage attenuated PRG942V-80 vaccine strain was compared with those of the 20th-, 40th-, and 60th-passage attenuated strains and the original virulent strain. [file 13567_2018_619_MOESM12_ESM.docx]

**Additional file 12 Comparison of full-length amino acid sequences of 11 genomic segments of PRG942V-80 (G9P[23]) vaccine strain with its different passages**

| Gene segment | | | Amino acid position | | Passage No. | | | | |
| --- | --- | --- | --- | --- | --- | --- | --- | --- | --- |
|  |  |  |  |  | PRG942  (1^st^) | PRG942V-20  (20^th^) | PRG942V-40  (40^th^) | PRG942V-60  (60^th^) | PRG942V-80  (80^th^) |
|  | | VP1 | | 135 | L | L | L | **P** | L |
|  |  | | 202 | | Y | Y | Y | Y | **H** |
|  |  |  | 228 | | E | E | E | E | **Y** |
|  |  |  | 238 | | S | S | **P** | S | S |
|  |  |  | 277 | | K | K | K | K | **Q** |
|  |  |  | 322 | | I | I | **M** | **M** | **I** |
|  |  |  | 323 | | V | V | **S** | **S** | V |
|  |  |  | 328 | | L | L | L | **W** | L |
|  |  |  | 330 | | R | R | R | **G** | R |
|  |  |  | 333 | | Q | Q | **P** | Q | Q |
|  | VP2 | | | 36 | K | K | **N** | K | K |
|  |  | | 39 | | I | **N** | **N** | I | **N** |
|  |  |  | 97 | | E | E | **K** | E | E |
|  |  |  | 110 | | E | E | E | **G** | E |
|  |  |  | 210 | | S | S | S | **P** | S |
|  |  |  | 216 | | C | C | **R** | **R** | **R** |
|  |  |  | 251 | | S | S | **L** | S | S |
|  |  |  | 278 | | I | I | I | I | **F** |
|  |  |  | 287 | | R | R | R | R | **G** |
|  |  |  | 288 | | N | **H** | N | N | N |
|  |  |  | 291 | | N | N | N | N | **I** |
|  |  |  | 292 | | Y | Y | Y | Y | **T** |
|  |  |  | 322 | | F | **L** | F | F | F |
|  |  |  | 324 | | Q | Q | **S** | **S** | **S** |
|  |  |  | 330 | | R | **T** | **T** | **T** | **T** |
|  |  |  | 334 | | F | **Y** | **L** | F | **Y** |
|  |  |  | 335 | | K | K | **L** | **L** | **I** |
|  |  |  | 340 | | V | V | V | **G** | V |
|  |  |  | 341 | | D | D | **V** | **V** | **V** |
|  |  |  | 342 | | S | S | **P** | **P** | **P** |
|  |  |  | 343 | | G | G | **D** | **D** | **D** |
|  |  |  | 347 | | E | E | **G** | E | E |
|  |  |  | 349 | | E | E | **K** | E | E |
|  | VP3 | | | 33 | L | **F** | L | L | **F** |
|  |  | | 210 | | G | **D** | G | G | G |
|  |  |  | 212 | | R | **G** | R | R | R |
|  |  |  | 237 | | H | H | H | H | **R** |
|  |  |  | 254 | | F | **L** | F | F | F |
|  |  |  | 285 | | V | **L** | V | V | **L** |
|  |  |  | 498 | | P | **L** | P | P | P |
|  |  |  | 519 | | D | D | **G** | D | D |
|  |  |  | 528 | | C | C | C | **W** | C |
|  |  |  | 531 | | I | I | I | I | **M** |
|  |  |  | 541 | | N | **K** | N | **K** | **K** |
|  |  |  | 557 | | Y | Y | Y | Y | **C** |
|  |  |  | 562 | | Q | Q | Q | **P** | Q |
|  |  |  | 571 | | R | R | R | R | **G** |
|  |  |  | 590 | | F | F | F | **L** | F |
|  |  |  | 607 | | N | N | N | N | **I** |
|  |  |  | 608 | | S | S | S | S | **L** |
|  |  |  | 613 | | V | V | V | V | **D** |
|  |  |  | 615 | | N | N | N | N | **I** |
|  |  |  | 654 | | E | E | E | **G** | **G** |
|  |  |  | 696 | | G | **D** | G | **D** | **D** |
|  |  |  | 753 | | Y | Y | Y | Y | **C** |
|  |  |  | 777 | | F | **L** | F | **L** | **L** |
|  |  |  | 799 | | R | **T** | R | **T** | **A** |
|  |  |  | 803 | | S | S | S | S | **G** |
|  |  |  | 807 | | T | T | T | **A** | **A** |
|  |  |  | 823 | | R | R | R | R | **G** |
|  | VP4 | | | 65 | Y | Y | Y | **C** | Y |
|  |  | | 80 | | Y | Y | Y | **C** | Y |
|  |  |  | 148 | | K | K | K | **R** | K |
|  |  |  | 171 | | T | T | **A** | T | T |
|  |  |  | 179 | | Y | Y | **C** | **C** | Y |
|  |  |  | 197 | | E | **G** | E | E | E |
|  |  |  | 212 | | I | I | **M** | I | I |
|  |  |  | 232 | | R | R | **G** | **G** | R |
|  |  |  | 250 | | T | **M** | **M** | **M** | **M** |
|  |  |  | 273 | | G | G | G | G | **R** |
|  |  |  | 280 | | R | **S** | R | R | R |
|  |  |  | 281 | | A | **F** | A | A | A |
|  |  |  | 282 | | N | **K** | N | N | N |
|  |  |  | 295 | | N | N | **H** | **H** | **H** |
|  |  |  | 296 | | Y | Y | **W** | **W** | **W** |
|  |  |  | 297 | | Q | Q | **R** | **R** | Q |
|  | VP7 | | | 7 | T | **Y** | T | T | T |
|  |  |  |  | 69 | N | N | N | N | **I** |
|  |  | | 97 | | E | E | **G** | E | **V** |
|  |  |  | 106 | | F | F | F | **L** | F |
|  |  |  | 115 | | A | **V** | **V** | **V** | **V** |
|  |  |  | 180 | | E | E | **G** | E | E |
|  | NSP1 | | | 46 | C | C | **R** | **R** | **R** |
|  |  | | 57 | | C | C | **W** | **W** | **W** |
|  |  |  | 126 | | R | R | **S** | **S** | **S** |
|  |  |  | 146 | | S | L | **L** | **L** | **L** |
|  |  |  | 229 | | S | **P** | S | S | S |
|  | NSP2 | | | 12 | L | L | L | **W** | L |
|  |  | | 13 | | E | E | E | **R** | E |
|  |  |  | 38 | | R | **K** | **K** | **K** | **K** |
|  |  |  | 61 | | S | **N** | **N** | **N** | **N** |
|  |  |  | 66 | | N | N | N | N | **S** |
|  |  |  | 113 | | P | **L** | **L** | **L** | **L** |
|  |  |  | 125 | | P | **L** | **L** | **L** | **L** |
|  |  |  | 277 | | K | K | K | K | **R** |
|  |  |  | 292 | | R | **K** | **K** | **K** | **K** |
|  |  |  | 312 | | F | **V** | **V** | **V** | **V** |
|  |  |  | 313 | | H | **S** | **S** | **S** | **S** |
|  |  |  | 314 | | R | **H** | **H** | **H** | **H** |
|  | NSP3 | | | 28 | G | **V** | **V** | **V** | **V** |
|  |  | | 93 | | T | T | T | T | **A** |
|  |  |  | 144 | | A | **T** | **T** | **T** | **T** |
|  |  |  | 188 | | S | **L** | **L** | **L** | **L** |
|  |  |  | 220 | | Q | Q | **R** | **R** | Q |
|  |  |  | 301 | | S | **L** | **L** | **L** | **L** |
|  | NSP4 | | | 88 | Q | Q | Q | Q | **R** |
|  | NSP5 | | | 20 | N | N | **S** | **S** | **S** |
|  |  | | 94 | | L | L | **S** | **S** | **S** |
|  |  |  | 176 | | K | K | **R** | K | K |
