# Supplementary material for: Development of a live attenuated trivalent porcine rotavirus A vaccine against disease caused by recent strains most prevalent in South Korea
Source: Vet Res. 2019 Jan 7;50:2. doi: 10.1186/s13567-018-0619-6 (PMC6323864; doi:10.1186/s13567-018-0619-6)
Supplement: Supplementary file 13 — Additional file 13. Comparison of full-length amino acid sequences of 11 genomic segments of K71V-80 (G9P[23]) vaccine strain with its different passages. The full-length amino acid sequences of the 11 genomic segments of the 80th-passage attenuated K71V-80 vaccine strain was compared with those of the 20th-, 40th-, and 60th-passage attenuated strains and the original virulent strain. [file 13567_2018_619_MOESM13_ESM.docx]

**Additional file 13 Comparison of full-length amino acid sequences of 11 genomic segments of K71V-80 (G5P[7]) vaccine strain with its different passage**

| Gene segment | | | Amino acid position | Passage No. | | | | |
| --- | --- | --- | --- | --- | --- | --- | --- | --- |
|  |  |  |  | K71  (1^st^) | K71V-20  (20^th^) | K71V-40  (40^th^) | K71V-60  (60^th^) | K71V-80  (80^th^) |
|  | | VP1 | 126 | A | **T** | **T** | **T** | **T** |
|  |  |  | 329 | S | **P** | S | S | S |
|  |  |  | 361 | N | N | N | N | **S** |
|  |  |  | 370 | E | E | E | E | **G** |
|  |  |  | 432 | R | R | **G** | R | R |
|  |  | | 445 | G | **R** | **R** | **R** | **R** |
|  |  |  | 818 | R | R | **Q** | **Q** | **Q** |
|  | VP2 | | 240 | V | V | **A** | V | V |
|  |  |  | 255 | P | **S** | P | P | P |
|  |  |  | 260 | F | F | F | **S** | F |
|  |  |  | 278 | I | I | I | I | **T** |
|  |  |  | 324 | S | S | S | **P** | S |
|  |  |  | 374 | M | **T** | **T** | **T** | **T** |
|  |  |  | 466 | S | S | **F** | **F** | **F** |
|  |  |  | 523 | P | P | **L** | **L** | **L** |
|  |  |  | 568 | M | M | **T** | **T** | **T** |
|  |  |  | 801 | K | K | **N** | **N** | **N** |
|  |  |  | 862 | S | S | **P** | **P** | **P** |
|  | VP3 | | 28 | E | E | **G** | **G** | E |
|  |  |  | 49 | S | **C** | S | S | S |
|  |  |  | 175 | E | **G** | E | E | E |
|  |  |  | 347 | S | S | **N** | **N** | **N** |
|  |  |  | 532 | F | F | **L** | **L** | **L** |
|  |  |  | 590 | F | F | **L** | **L** | **L** |
|  |  |  | 724 | Q | Q | **L** | **L** | **L** |
|  | VP4 | | 98 | S | S | **N** | S | S |
|  |  |  | 230 | A | A | **T** | **T** | **T** |
|  |  |  | 315 | H | H | H | H | **L** |
|  |  |  | 532 | P | P | **S** | **S** | **S** |
|  |  |  | 559 | V | V | **A** | **A** | **A** |
|  |  |  | 687 | G | G | **D** | **D** | **D** |
|  | VP6 | | 8 | P | **S** | **S** | **S** | **S** |
|  |  |  | 120 | T | **S** | **S** | **S** | **S** |
|  |  |  | 248 | Y | Y | Y | **C** | Y |
|  | VP7 | | 24 | R | **K** | **K** | **K** | **K** |
|  |  |  | 201 | Q | Q | Q | **R** | **R** |
|  | NSP1 | | 3 | G | G | **S** | **S** | **S** |
|  | NSP2 | | 24 | S | S | **N** | S | S |
|  |  |  | 59 | K | K | K | K | **R** |
|  |  |  | 75 | P | **S** | **S** | **S** | **S** |
|  |  |  | 153 | E | **K** | E | E | E |
|  |  |  | 161 | T | **N** | **N** | **N** | **N** |
|  |  |  | 162 | A | A | **T** | A | A |
|  |  |  | 177 | M | M | M | M | **T** |
|  |  |  | 200 | A | A | **V** | A | A |
|  |  |  | 207 | A | A | A | A | **T** |
|  |  |  | 271 | A | A | A | **T** | A |
|  |  |  | 314 | H | H | **R** | H | H |
|  | NSP3 | | 12 | S | S | **S** | **G** | **G** |
|  |  |  | 68 | A | A | A | **M** | **M** |
|  |  |  | 81 | T | T | T | **V** | **V** |
|  |  |  | 107 | I | I | I | **M** | **M** |
|  |  |  | 146 | R | R | R | **K** | **K** |
|  |  |  | 172 | A | A | A | **S** | **S** |
|  |  |  | 189 | N | N | N | **S** | **S** |
|  |  |  | 236 | S | S | S | **N** | **N** |
|  |  |  | 266 | S | S | S | **L** | **L** |
|  |  |  | 268 | S | S | S | **P** | **P** |
|  |  |  | 296 | F | F | F | **S** | **S** |
|  |  |  | 310 | C | C | C | **Y** | **Y** |
|  |  |  | 311 | T | T | T | **A** | **A** |
|  | NSP4 | | 123 | Q | Q | Q | L | L |
|  |  |  | 146 | K | K | K | R | R |
|  |  |  | 160 | G | G | E | E | E |
|  | NSP5 | | 4 | S | S | S | S | **G** |
|  |  |  | 124 | S | S | S | S | **P** |
|  |  |  | 128 | I | I | I | **T** | I |
|  |  |  | 166 | V | **D** | **D** | **D** | **D** |
|  |  |  | 190 | A | A | A | **V** | A |
