# Supplementary material for: Development of a live attenuated trivalent porcine rotavirus A vaccine against disease caused by recent strains most prevalent in South Korea
Source: Vet Res. 2019 Jan 7;50:2. doi: 10.1186/s13567-018-0619-6 (PMC6323864; doi:10.1186/s13567-018-0619-6)
Supplement: Supplementary file 15 — Additional file 15. Summary of the histopathological findings in the small intestine of the colostrums-deprived neonatal piglets vaccinated with a live attenuated monovalent vaccine strain (PRG942V-80) or filtered supernatant of a mixture of homogenized small intestine and feces sampled from each passage. [file 13567_2018_619_MOESM15_ESM.docx]

**Additional file 15** **Summary of the histopathological findings in the small intestine of the colostrums-deprived neonatal piglets vaccinated with a live attenuated monovalent vaccine strain (PRG942V-80) or filtered supernatant of a mixture of homogenized small intestine and feces sampled from each passage**

| Passage | Piglet  No. | dpi^a^ at euthanasia | Duodenum | Jejunum | Ileum |
| --- | --- | --- | --- | --- | --- |
|  |  |  | Lesion score^b^ | Lesion score^b^ | Lesion score^b^ |
| 1^st^ passage | 1 | 5 | 0.1 | 0.2 | 0.2 |
|  | 2 | 5 | 0 | 0.4 | 0.4 |
| 2^nd^ passage | 3 | 5 | 0.4 | 0.4 | 0.6 |
|  | 4 | 5 | 0.2 | 0.2 | 0.2 |
| 3^rd^ passage | 5 | 5 | 0.2 | 0 | 0.2 |
|  | 6 | 5 | 0.4 | 0.4 | 0.4 |
| 4^th^ passage | 7 | 5 | 0.6 | 0.2 | 0 |
|  | 8 | 5 | 0.2 | 0.2 | 0.4 |
| 5^th^ passage | 9 | 5 | 0.3 | 0.4 | 0.1 |
|  | 10 | 5 | 0.2 | 0.4 | 0.2 |

^a^ dpi: Days post-inoculation.

^b^ The small intestinal changes were scored according to the average villi/crypt (V/C) ratio plus the grade of epithelial cell desquamation, which was measured as follows: V/C ratio, 0 = normal (V/C≧6:1), 1 = mild (V/C = 5.0 to 5.9:1), 2 = moderate (V/C = 4.0 to 4.9:1), 3 = marked (V/C = 3.0 to 3.9:1), 4 = severe (V/C ≦3.0:1) and desquamation grade, 0 = normal (no desquamation), 1 = mild (cuboidal attenuation of tip villous epithelium), 2 = moderate (desquamation of upper villous epithelium), 3 = marked (desquamation of lower villous epithelium), 4 = severe (desquamation of crypt epithelium).
